# A genetic chronology for the Indian Subcontinent points to heavily sex-biased dispersals

**DOI:** 10.1186/s12862-017-0936-9

**Published:** 2017-03-23

**Authors:** Marina Silva, Marisa Oliveira, Daniel Vieira, Andreia Brandão, Teresa Rito, Joana B. Pereira, Ross M. Fraser, Bob Hudson, Francesca Gandini, Ceiridwen Edwards, Maria Pala, John Koch, James F. Wilson, Luísa Pereira, Martin B. Richards, Pedro Soares

**Affiliations:** 1 Department of Biological Sciences, School of Applied Sciences, University of Huddersfield, Queensgate, Huddersfield, HD1 3DH UK; 2 i3S (Instituto de Investigação e Inovação em Saúde, Universidade do Porto), R. Alfredo Allen 208, 4200-135 Porto, Portugal; 3 IPATIMUP (Instituto de Patologia e Imunologia Molecular da Universidade do Porto), Rua Júlio Amaral de Carvalho 45, 4200-135 Porto, Portugal; 4 Department of Informatics, University of Minho, Campus de Gualtar, 4710-057 Braga, Portugal; 5 CBMA (Centre of Molecular and Environmental Biology), Department of Biology, University of Minho, Campus de Gualtar, 4710-057 Braga, Portugal; 6 Life and Health Sciences Research Institute (ICVS), School of Health Sciences, University of Minho, Campus de Gualtar, 4710-057 Braga, Portugal; 7 ICVS/3B’s—PT Government Associate Laboratory, Braga/Guimarães, Portugal; 8 Centre for Global Health Research, Usher Institute of Population Health Sciences and Informatics, University of Edinburgh, Teviot Place, Edinburgh, EH8 9AG Scotland UK; 9 Synpromics Ltd, Nine Edinburgh Bioquarter, Edinburgh, EH16 4UX UK; 10 Archaeology Department, University of Sydney, Sydney, NSW 2006 Australia; 11 University of Wales Centre for Advanced Welsh and Celtic Studies, National Library of Wales, Aberystwyth, SY23 3HH Wales, UK; 12 MRC Human Genetics Unit, Institute of Genetics and Molecular Medicine, University of Edinburgh, Western General Hospital, Edinburgh, EH4 2XU Scotland, UK

**Keywords:** Mitochondrial DNA, Indian Subcontinent, Genome-wide, Y chromosome, Neolithic, Indo-European

## Abstract

**Background:**

India is a patchwork of tribal and non-tribal populations that speak many different languages from various language families. Indo-European, spoken across northern and central India, and also in Pakistan and Bangladesh, has been frequently connected to the so-called “Indo-Aryan invasions” from Central Asia ~3.5 ka and the establishment of the caste system, but the extent of immigration at this time remains extremely controversial. South India, on the other hand, is dominated by Dravidian languages. India displays a high level of endogamy due to its strict social boundaries, and high genetic drift as a result of long-term isolation which, together with a very complex history, makes the genetic study of Indian populations challenging.

**Results:**

We have combined a detailed, high-resolution mitogenome analysis with summaries of autosomal data and Y-chromosome lineages to establish a settlement chronology for the Indian Subcontinent. Maternal lineages document the earliest settlement ~55–65 ka (thousand years ago), and major population shifts in the later Pleistocene that explain previous dating discrepancies and neutrality violation. Whilst current genome-wide analyses conflate all dispersals from Southwest and Central Asia, we were able to tease out from the mitogenome data distinct dispersal episodes dating from between the Last Glacial Maximum to the Bronze Age. Moreover, we found an extremely marked sex bias by comparing the different genetic systems.

**Conclusions:**

Maternal lineages primarily reflect earlier, pre-Holocene processes, and paternal lineages predominantly episodes within the last 10 ka. In particular, genetic influx from Central Asia in the Bronze Age was strongly male-driven, consistent with the patriarchal, patrilocal and patrilineal social structure attributed to the inferred pastoralist early Indo-European society. This was part of a much wider process of Indo-European expansion, with an ultimate source in the Pontic-Caspian region, which carried closely related Y-chromosome lineages, a smaller fraction of autosomal genome-wide variation and an even smaller fraction of mitogenomes across a vast swathe of Eurasia between 5 and 3.5 ka.

**Electronic supplementary material:**

The online version of this article (doi:10.1186/s12862-017-0936-9) contains supplementary material, which is available to authorized users.

## Background

Following the out-of-Africa (OOA) migration, South Asia (or the Indian Subcontinent, here comprising India, Pakistan, Bangladesh, Sri Lanka, Nepal and Bhutan) was probably one of the earliest corridors of dispersal taken by anatomically modern humans (AMH) [1–3]. A remarkable genetic diversity, probably the second highest after sub-Saharan populations [1, 4] supports this view. Although the oldest modern human fossils in South Asia (in Sri Lanka) date to only ~36–28 thousand years ago (ka) [5, 6], genetic and archaeological evidence suggest an arrival of AMH over 50 ka (discussed extensively in Mellars et al. [2]) but after the eruption of Mount Toba in Sumatra ~74 ka, contrary to some suggestions [7]. Whilst some argue for a hint of an earlier dispersal [8], the trace is restricted to Australia/New Guinea, where it amounts to only ~2% of the data, and its significance remains unclear [9, 10].

India, the second most populous country worldwide, includes a patchwork of different religions and languages, including tribal groups (~8% of the population, speaking over 700 different dialects of the Austro-Asiatic, Dravidian and Tibeto-Burman families) and non-tribal populations, who mostly practice Hinduism, grounded in a strictly hierarchical caste system, and speak Indo-European or Dravidian languages. Indo-European is often associated with northern Indian populations, Pakistan and Bangladesh, and a putative arrival in South Asia from Southwest Asia ~3.5 ka (the so-called “Indo-Aryan invasions”) has been frequently connected with the origins of the caste system [11, 12]. Although some studies suggested a greater affinity of upper castes to European and Southwest Asian populations than lower castes [13, 14], genetic data have provided no clear evidence for the “Indo-Aryan invasions” so far [15], and their very existence is challenged by many archaeologists [16].

South India, on the other hand, is dominated by Dravidian languages, which have been connected to Neolithic dispersals from Southwest Asia [1, 12, 17], although the South Asian situation is complex and others have argued for indigenous development of agriculture within the Dravidian heartland [18, 19]. Generally, India displays a high level of endogamy, a result of its strict social boundaries, and high genetic drift due to long-term isolation [20] which, combined with a very complex history, makes the genetic study of Indian populations challenging. Many recent genetic studies explored different layers of South Asian genetic diversity and population structure [2, 13–15, 17, 21–26], but they have tended to focus on one or other marker system and, as a result, decisive results on the details of the settlement process are still lacking.

In the last few years, genome-wide (GW) studies have been employed [27–29]. However, it remains difficult to make inferences concerning the timing and direction of migrations from GW results, without including ancient DNA (aDNA) data (still lacking for South Asia), and for India the results have been contradictory, especially for differentiating amongst various migration waves at greater time depths.

There is a way forward, despite the current lack of aDNA. The maternally inherited mitochondrial DNA (mtDNA) allows researchers to identify specific lineage clusters (clades or haplogroups) and to correlate them with geography. By applying a reliable mitogenome molecular clock [30], it is then possible to date migration events and uncover fine demographic patterns that would otherwise be missed. Previous studies [2, 31, 32] revealed that South Asian mtDNA diversity consists largely of basal autochthonous lineages of the OOA founder haplogroups M and N (the latter mostly from the derived haplogroup R) [20]. Moreover, similar analyses can be carried out for the paternally inherited Y-chromosome variation, and comparisons of the two systems can detect sex bias in dispersal patterns.

To assess the phylogeographic patterns of South Asian mtDNA lineages, we compiled mitogenomes from South Asia and neighbouring regions available in the literature, complemented with samples from the 1000 Genomes Project (1KGP) [33] and the Human Genome Diversity Project (HGDP) [34], including understudied populations from Pakistan, Sri Lanka and Bangladesh, combined with several newly sequenced samples. We aimed to provide a refined mtDNA phylogeographic portrait of South Asia, including most crucially an assessment of the extent of genetic influx from other regions (primarily Southwest and Central Asia), in order to assess the impact of immigration during the Late Glacial, postglacial, Neolithic and Bronze Age periods in shaping genetic diversity and structure in South Asia. For a comprehensive overview across the genome, we have also carried out several fresh analyses of GW patterns across the regions of Southwest, Central and South Asia, and assessed sex-biased gene flow in the region by direct comparison across the same sample sets, using the 1KGP data now available for GW, mtDNA and Y-chromosome diversity.

## Methods

### Mitogenome dataset

In order to clarify the phylogeny of haplogroups M, N and R in South Asia, we focused our study on the lineages with recognized or potential likely origin in the Subcontinent, belonging to macrohaplogroups M (M2, M3, M4’67, M5, M6, M13’46’61, M31, M32’56, M33, M34’57, M35, M36, M39, M40, M41, M42b, M44, M48, M49, M50, M52, M53, M58, M62), R (R5, R6, R7, R8, R30 and R31) and N (N1’5). We also studied U2 (excluding U2e due to its West Eurasian origin) in a complementary analysis. We obtained 381 whole-mtDNA sequences from the 1KGP [33] (although we note that these were collected from caste families from India and lack tribal groups) and 51 from the HGDP [34]. In addition, we generated 13 new sequences (accession numbers: KY686204 -KY686216) belonging to the aforementioned haplogroups from Southeast Asia: seven from Myanmar, one from Vietnam, one from Thailand and four from Indonesia. We combined these with other published data from South Asia and neighbouring areas, including a total of 1478 samples (Additional file 1: Table S1). The additional sequences increased substantially the sample size particularly in the West of the Indian Subcontinent, necessitating a re-evaluation of previously inferred phylogeographic patterns [2, 35].

In order to discern migrations into the Subcontinent at different time periods, we also performed a complementary analysis of several “non-autochthonous” N lineages present in South Asia (H2b, H7b, H13, H15a, H29, HV, I1, J1b, J1d, K1a, K2a, N1a, R0a, R1a, R2, T1a, T2, U1, U7, V2a, W and *X*2—all subclades of West Eurasian haplogroups), amounting to a total of 635 mtDNA sequences (Additional file 1: Table S2). We assigned haplogroups using HaploGrep [36], in accordance with the nomenclature in PhyloTree (Build 17, February 2016) [37].

### Phylogenetic reconstruction and statistical analyses of mtDNA

We reconstructed the mitogenome phylogenetic tree manually, based on a preliminary reduced-median network analysis [38] with Network v.4.611, checked considering the frequency of each mutation [30] and the nomenclature of PhyloTree (Build 17) [37]. We estimated coalescence ages within haplogroups M and N using both the ρ statistic [39] and maximum likelihood (ML). We calculated ρ estimates with standard errors estimated as in Saillard et al. [40] using a synonymous clock of one substitution in every 7884 years and a mitogenome clock of one substitution every 3624 years further corrected for purifying selection [30]. We assessed ML estimations using PAML 4 and the same mitogenome clock assuming the REV mutation model with gamma-distributed rates (discrete distribution of 32 categories) and two partitions, in order to distinguish hypervariable segments I and II (HVS–I and HVS–II) from the rest of the molecule. We performed runs both assuming and not assuming a molecular clock, in order to perform likelihood ratio tests (LRT) [41].

Since haplogroup M displays a peculiar phylogeographic pattern in South Asia [2], we additionally estimated node ages in different sub-regions of the Subcontinent (west, south, central and east) with two different approaches: (1) considering all samples from a given region, regardless of the putative geographical origin of the clade and (2) considering the most probable origin of each major haplogroup (by considering branching structure, number of main branches, and centre of gravity) and including only basal lineages of each region [2]. To evaluate the effective population size (*N*_*e*_) of haplogroup M in each region, we computed Bayesian Skyline Plots (BSPs) [42] using BEAST 1.8.0 [43]. Although haplogroups do not equate to populations, BSPs applied to specific lineages can provide insights into the size variations of the populations that include them [44–47]. We used a relaxed molecular clock (lognormal in distribution across branches and uncorrected between them), a two-parameter nucleotide evolution model and a mutation rate of 2.514 x 10^-8^ mutations per site per year [48].

### GW dataset and analysis

We filtered a dataset comprising 1440 samples with 500,123 SNPs, combining data from the 1KGP and 8 independent studies (Additional file 1: Table S3) for linkage disequilibrium (LD) using PLINK v1.07 [49] (r2 > 0.25, with a window size of 100 SNPs and step size of 1), yielding a subset containing 164,149 SNPs. We subjected these to principal component analysis (PCA) using the standard PCA tool provided in EIGENSOFT v6.0.1 [50], with which we calculated the first 10 principal components (PCs), from which we calculated the fraction of variance. We included three additional 1KGP populations—Han Chinese from Beijing, China (CHB), Tuscans from Italy (TSI) and Yoruba from Nigeria (YRI)—for ADMIXTURE v1.23 [51] and sNMF [52] analyses for cross-checking. We performed runs for values of *K* between 2 and 10, with 5-fold cross-validation in ADMIXTURE, and complementary analyses including Yamnaya aDNA samples [53]. The filtered datasets used (r2 > 0.25, window size of 100 SNPs and step size of 1) included 66,245 SNPs, for ADMIXTURE analysis, and 64,926 SNPs for the PCA.

In order to assess potential sex-biased gene flow into the region, we compared uniparental (mtDNA and Y-chromosome) and autosomal ancestry in the five 1KGP South Asian populations: Bengali from Bangladesh (BEB), Gujarati Indian from Houston (GIH), Indian Telugu from the UK (ITU), Punjabi from Lahore, Pakistan (PJL) and Sri Lankan Tamil from the UK (STU). For the autosomal ancestry variation, we considered the mean of each component for the highest likelihood value. The putative origin of the uniparental lineages present in the populations is shown in Additional file 1: Table S4. Y-chromosome phylogeny was based on Yfull tree v4.10 (https://www.yfull.com/tree/) [54]. We considered as South Asian the Y-chromosome lineages that most likely entered the Subcontinent before the Last Glacial Maximum (LGM): H [55–57], K2a1* [58] (this attribution on the basis of the early-branching lineage, and therefore uncertain, but only concerns a single sample and does not affect the results in any way), and C5 [58]. Y-chromosome haplogroups G, J, L1, L3, Q, R1 and R2 seem to have entered South Asia more recently in the early to mid-Holocene from a West Eurasian source [17, 55–59]. C(xC5), O and N probably had a Holocene Eastern origin [55, 58, 60, 61].

## Results

### Indigenous South Asian mtDNA lineages: An explanation for the anomalous age of haplogroup M

**Fig. 1:**
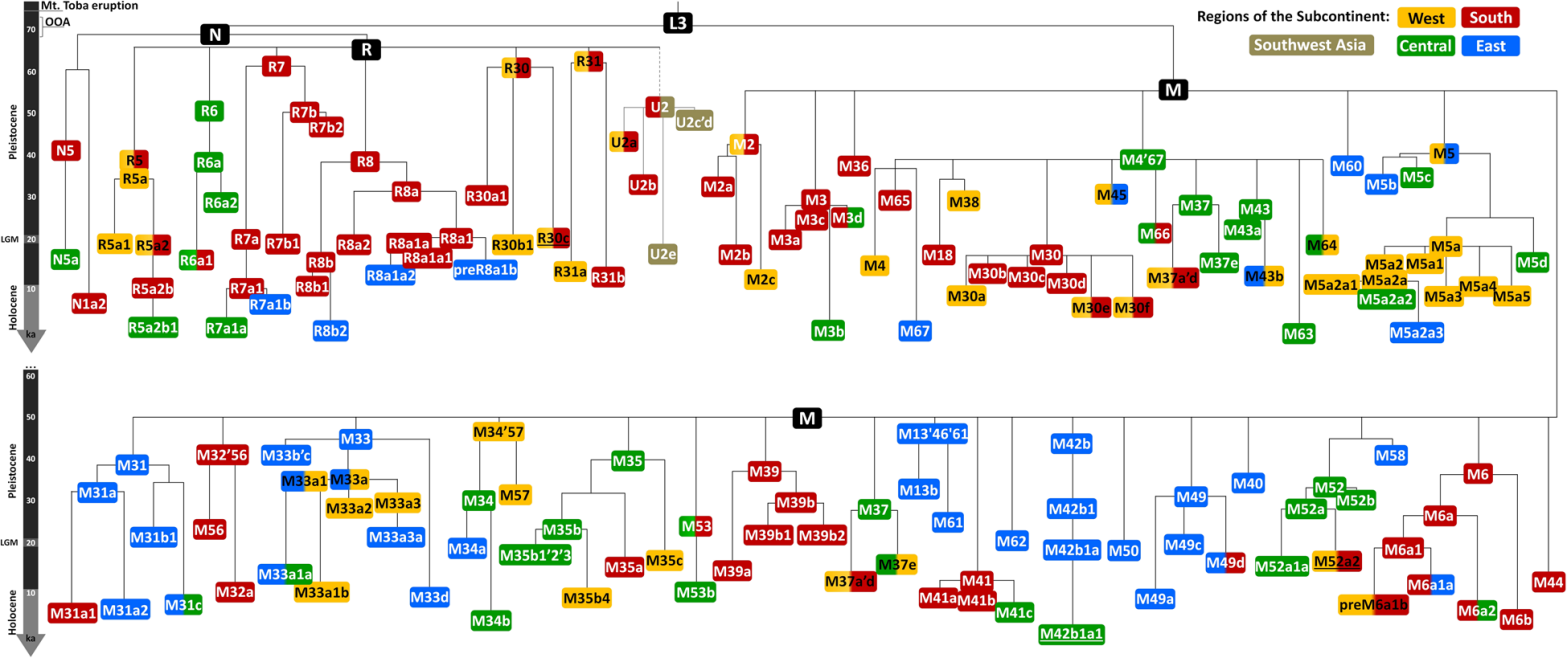
Schematic phylogeny of South Asian autochthonous mtDNA haplogroups, based on ML age estimates. Node ages for haplogroup U2 were estimated in an independent analysis. Colours correspond to the putative origin of each branch

The complete phylogeny for autochthonous South Asian M, N and R lineages is shown in Additional file 2 including age estimates for the main nodes (using ρ and ML age estimates). Age estimates for clades mentioned in the text are shown in Table 1 and a schematic phylogenetic tree scaled by ML age estimates is shown in Fig. 1.

**Table 1:** Age estimates (in ka) of the clades mentioned in the text. Node ages for haplogroup U2 were estimated in an independent analysis

Clade	ML	ρ whole mtDNA	ρ synonymous clock
N	67.7 [58.4–77.1]	63.5 [51.7–75.7]	71.5 [51.3–91.8]
R	64.5 [55.9–73.2]	57.0 [48.6–65.5]	63.5 [49.1–77.8]
R7	62.2 [52.9–71.7]	62.0 [43.0–81.6]	76.0 [42.2–109.8]
R8b1	12.0 [7.0–17.1]	11.1 [5.8–16.5]	5.1 [2.1–8.1]
R30	60.9 [49.6–72.5]	53.0 [40.6–65.8]	61.5 [40.5–82.6]
R30c + 373	8.6 [0.0–48.1]	9.0 [3.5–14.6]	6.3 [0.5–12.1]
R31	62.5 [53.0–72.1]	70.8 [50.4–92.0]	75.2 [43.3–107.1]
M	50.1 [44.8–55.5]	41.2 [37.0–45.4]	41.3 [34.6–48.0]
M2	43.2 [34.7–52.0]	51.2 [35.8–67.3]	44.5 [23.2–65.8]
M2a1a1b	22.0 [0.0–6.0]	3.3 [0.0–7.7]	3.4 [0.0–10.0]
M2a1b	0.7 [0.0–2.5]	0.6 [0.0–1.5]	1.0 [0.0–2.9]
M2a3a + 4314	0.9 [0.0–2.8]	0.9 [0.0–2.5]	–
M2c + 1888 + 146	2.5 [0.0–19.9]	3.5 [0.0–8.4]	10.5 [0.0–25.1]
M3a1 + 204 + 14476	1.2 [0.0–2.7]	1.0 [0.0–2.0]	2.4 [0.0–5.0]
M3a1 + 204 + 10845 + 13105	0.9 [0.0–3.3]	0.9 [0.0–2.6]	0.0
M3b	1.8 [0.0–4.5]	2.2 [0.0–5.7]	5.5 [0.0–15.6]
M4’67	38.0 [30.1–46.0]	27.8 [23.4–32.3]	22.7 [18.3–27.0]
M5a1b1a1 (M5a1b + 3954 + 9833 + 16298)	3.0 [1.0–5.0]	2.7 [1.4–4.1]	2.3 [0.0–4.7]
M5a2a + 8158 + 199	1.9 [0.7–3.2]	1.8 [0.7–2.8]	3.0 [0.6–5.3]
M5a2a2 + 234	1.5 [0.0–4.2]	1.4 [0.2–2.7]	2.6 [0.0–5.6]
M5a3a	0.7 [0.0–3.3]	–	–
M5a3b	1.6 [0.0–3.5]	1.5 [0.1–3.0]	1.6 [0.0–3.8]
M5b	33.0 [23.6–42.9]	30.7 [20.9–40.9]	36.9 [17.7–56.2]
M5c	35.2 [24.2–46.6]	41.5 [28.2–55.3]	49.3 [25.0–73.6]
M6	35.6 [25.9–45.7]	37.9 [23.4–53.2]	48.7 [19.6–77.9]
M6a1 + 5585 + 146 + 1508	1.3 [0.0–3.2]	1.1 [0.0–2.3]	0.9 [0.0–2.6]
M6a1a	11.4 [4.0–19.2]	10.6 [6.6–14.7]	10.3 [4.9–15.8]
M13b	32.8 [21.5–44.5]	30.7 [17.1–45.2]	33.8 [12.2–55.4]
M18a	9.2 [6.0–12.4]	8.1 [5.6–10.5]	6.0 [2.1–10.0]
M30a2	2.3 [0.0–8.5]	1.9 [0.0–4.8]	–
M30d	11.4 [4.6–18.5]	9.2 [4.1–14.3]	10.0 [2.8–17.2]
M31	38.0 [27.9–48.4]	38.4 [25.9–51.4]	43.6 [20.6–66.7]
M32’56	42.4 [25.8–60.0]	33.0 [16.7–50.4]	14.5 [0.5–28.4]
M33a	35.2 [24.5–46.3]	29.1 [21.2–37.2]	32.3 [19.3–45.3]
M34	29.7 [19.4–40.4]	28.1 [17.6–39.1]	39.4 [17.9–60.9]
M35	40.1 [25.4–55.5]	26.9 [18.5–35.6]	26.4 [15.5–37.3]
M36	36.4 [25.8–47.4]	26.9 [16.2–38.2]	30.6 [11.6–49.6]
M38	29.4 [20.4–38.7]	32.5 [23.6–41.7]	33.8 [19.4–48.2]
M39	36.8 [27.3–46.6]	23.7 [15.3–32.5]	21.2 [9.1–33.2]
M42b	42.5 [33.8–51.4]	43.5 [27.1–60.8]	49.7 [22.4–77.1]
M45	30.6 [19.0–42.8]	30.7 [18.5–43.6]	33.8 [14.1–53.5]
M49	31.0 [21.2–41.2]	26.3 [18.1–34.8]	25.6 [13.6–37.5]
M50	43.3 [30.6–56.6]	47.4 [32.3–63.3]	52.0 [26.4–77.7]
M52	33.4 [23.4–43.9]	31.0 [22.1–40.2]	33.4 [19.0–47.9]
M57	32.4 [18.2–47.3]	28.8 [19.0–38.9]	24.5 [11.5–37.6]
M60	36.5 [23.3–50.4]	24.8 [15.8–34.2]	21.0 [8.9–33.2]
M61	24.6 [13.6–36.2]	11.8 [6.0–17.8]	12.4 [1.4–23.4]
M61 + 5294	1.6 [0.0–5.1]	1.9 [0.0–4.8]	2.0 [0.0–5.8]
M63	1.4 [0.0–3.8]	1.3 [0.0–2.8]	1.3 [0.0–3.9]
M65	29.3 [14.7–44.8]	20.6 [12.6–29.0]	21.3 [8.4–34.1]
N1a2	12.5 [2.9–22.6]	6.5 [2.1–11.2]	7.9 [0.2–15.6]
N1a1b1	20.9 [11.4–30.8]	19.0 [10.4–27.9]	22.1 [7.6–36.6]
H2b	6.2 [3.8–8.7]	5.2 [3.4–7.1]	4.8 [1.7–7.9]
H13a2a + 8952	6.6 [1.3–12.1]	7.2 [1.0–13.6]	2.0 [0.0–5.8]
H29 + 9156 + 4689	1.6 [0.0–4.7]	1.3 [0.0–3.8]	3.9 [0.0–11.7]
HV + 73	23.7 [17.1–30.4]	30.1 [19.6–41.0]	29.8 [12.1–47.5]
HV + 146	23.9 [10.3–38.4]	19.0 [8.8–29.8]	11.8 [0.0–25.2]
HV + 9716	19.6 [8.1–31.8]	13.4 [5.0–22.2]	3.9 [0.0–11.7]
HV + 16311	15.6 [9.9–21.5]	15.5 [7.6–23.8]	19.3 [3.4–35.1]
HV2	21.9 [15.1–28.9]	30.7 [17.9–44.2]	38.1 [12.2–64.0]
HV12b	13.3 [5.3–21.6]	12.6 [5.7–19.8]	5.6 [0.7–10.6]
HV14 + 150	6.9 [2.9–11.0]	6.7 [1.0–12.6]	11.4 [0.0–25.7]
I1	13.8 [8.5–19.2]	10.6 [6.3–15.0]	11.8 [4.1–19.6]
J1b1b1	13.9 [8.6–19.3]	12.6 [7.9–17.4]	12.4 [5.1–19.7]
J1d	24.1 [14.9–33.7]	16.2 [10.2–22.3]	17.3 [7.1–27.6]
K1a1b2a	10.4 [4.0–17.0]	12.0 [4.1–20.3]	7.9 [0.0–18.8]
K2a5	7.6 [3.6–11.7]	8.2 [3.9–12.6]	5.3 [1.1–9.5]
K2a5 + 2831	6.8 [2.9–10.7]	8.4 [3.5–13.5]	4.7 [0.0–10.1]
K2a5 + 2831 + 189	5.9 [2.1–9.8]	10.6 [3.2–18.4]	7.9 [0.0–18.8]
R0a2 + 11152	7.1 [1.1–13.3]	6.5 [0.8–12.5]	7.9 [0.0–18.8]
R2a + 7142	3.2 [0.0–6.9]	2.9 [0.0–5.9]	1.8 [0.0–4.2]
T2 + 195 + 4225	9.7 [2.9–16.8]	6.8 [2.3–11.5]	3.2 [0.0–7.5]
T2b	10.6 [5.3–16.0]	7.1 [3.6–10.8]	3.4 [0.0–7.2]
T2d1a	12.0 [5.0–19.3]	10.6 [4.5–16.9]	7.9 [0.0–16.8]
T2e2	10.6 [3.4–18.1]	12.0 [4.1–20.3]	11.8 [0.0–25.2]
U1a1	20.0 [14.4–25.7]	15.2 [10.4–20.1]	15.2 [6.2–24.3]
U1a1a2a	2.5 [0.0–7.3]	1.9 [0.0–4.8]	5.9 [0.0–14.6]
U1a3 + 10253	10.3 [4.6–16.2]	8.9 [4.6–13.3]	10.8 [2.9–18.8]
U1a3a	5.2 [0.0–11.0]	3.9 [0.0–8.4]	3.9 [0.0–11.7]
Pre-U1c	21.4 [9.1–34.5]	14.3 [6.7–22.2]	13.1 [1.6–24.7]
U2	52.3 [41.6–63.3]	53.8 [41.8–66.2]	54.1 [36.6–71.6]
U2b2	9.2 [6.3–12.2]	8.6 [6.1–11.1]	9.9 [5.3–14.4
U2c1 + 146	1.4 [0.0–24.8]	1.7 [0.0–5.1]	–
U7a	18.1 [14.4–22.0]	18.8 [14.5–23.2]	19.7 [11.5–27.9]
U7a + 12373	10.2 [3.0–17.6]	8.8 [2.8–15.0]	10.5 [0.0–23.1]
U7a3a + 6150	9.8 [4.4–15.4]	8.6 [3.5–13.8]	2.0 [0.0–5.8]
U7b + 16309!	10.9 [6.1–15.9]	8.6 [3.6–13.8]	8.4 [0.0–18.1]
W3a1 + 143	9.8 [3.0–16.8]	7.9 [1.5–14.5]	19.7 [2.4–37.0]
W3a1 + 1709	8.1 [1.6–15.0]	6.5 [0.8–12.5]	–
W3a1b	11.4 [6.3–16.6]	11.2 [6.1–16.3]	7.1 [1.1–13.1]
W4	15.8 [9.5–22.3]	15.5 [8.7–22.5]	11.8 [2.4–21.3]
W6	11.5 [5.0–18.3]	10.9 [5.7–16.3]	13.1 [6.5–19.8]
*X*2 + 153 + 7109	7.7 [0.0–17.0]	4.3 [0.0–9.0]	2.6 [0.0–7.8]

Although haplogroup M in Asia has been shown to depart from a strict molecular clock [62], we found no evidence for a clock violation when performing a LRT (*p* > 0.05). Curiously, however, we found violations to the molecular clock for South Asian R lineages (*p* < 0.00001). Since ML analysis is partly based on the tree structure, it averages the branch lengths and provides similar estimates to a previous relaxed clock [63]. The values indicated throughout the text are therefore ML estimates (corrected for purifying selection). This is not observed in the global mtDNA tree [30, 64] and seems peculiar to the haplogroups in South Asia, due to demographic effects, as we argue below.

There are two major founder clades detected in South Asia (haplogroup N is very rare and its age does not correspond to a founder age). As previously, the age of haplogroup M, at 50.1 [44.8; 55.5] ka, and R, at 64.5 ka [55.9; 73.2] are younger than the Mount Toba eruption (~74 ka), suggesting a later arrival [2]. Haplogroup R and several of its subclades (R7, R30, R31) appear older than M, but this may be illusory—see below. The older clades in R predominate in the west and south of the Subcontinent, supporting a southern coastal route of primary colonization [1–3].

The phylogeography of haplogroup M is complex. While some older lineages (e.g. M2, M6, M32’56, M36, M39) originated in the western or southern regions of the Subcontinent (similarly to R), others trace to central India (M4’67, M35, M52) or the east (M13b, M31, M42b, M61, M49, M50 and M60). We need to tease out these more detailed patterns to explain the discrepancy in the age estimates.

If we perform regional estimates simply by considering all samples of each region, no discernible patterns are apparent, with M age estimates in the south and east showing similar ages (Table 2). However, when we take into account the inferred source for each clade and repartition the data on that basis, the re-estimated age for M in the west becomes 55.3 [45.1; 65.9] ka—higher than across the rest of the Subcontinent (Table 2). This suggests an early expansion in the west, similar to R, and a common origin and spread of both M and R along the southern coastal route, as also suggested recently from analyses of ancient DNA (aDNA) [65]. Although M has previously been dated to an earlier age in East Asia [30, 66], the lower age of M in the east of the Subcontinent *versus* the west argues against an eastern origin of M as recently proposed [35].

**Table 2:** Age estimates (in ka) of haplogroup M in different regions of South Asia: (1) using the raw modern geographic distribution and (2) considering the most probable origin of each major haplogroup and including only basal lineages of each region

		ML	ρ whole mtDNA	ρ synonymous clock
(1)	West	47.7 [41.3–54.2]	37.4 [31.6–43.2]	39.0 [28.8–49.2]
	South	47.2 [41.5–53.1]	42.4 [36.7–48.3]	40.0 [31.4–48.6]
	East	47.7 [42.5–53.0]	42.4 [38.4–46.6]	43.9 [37.1–50.8]
	Central	43.6 [38.1–49.1]	40.8 [35.4–46.3]	41.4 [33.0–49.7]
(2)	West	55.3 [45.1–65.9]	44.5 [32.5–57.0]	50.6 [29.7–71.4]
	South	48.9 [42.1–55.8]	47.5 [39.2–56.0]	41.1 [29.6–52.6]
	East	45.2 [38.8–51.8]	40.8 [34.6–47.0]	40.1 [31.3–48.9]
	Central	39.5 [31.9–47.2]	33.0 [26.8–39.3]	34.80 [23.2–46.5]

This result suggests that an ancient western ancestry may have been disguised by further re-expansions of haplogroup M in South Asia. Several branches of M (M38, M65, M45, M5b, M5c, M34, M57, M33a) display signals of dispersals from the east and the centre dating to ~45–35 ka, and M4’67 (which is only separated by a single mutation from the root of M), with a possible origin in central India, displays an extraordinary multi-branching structure dating to 38.0 [30.1; 46.0] ka, suggesting a major expansion at that time. If we consider that a root type of M could have survived for ~10,000 years after it arose (as is evident from modern clades within that age range), it is plausible that re-expansion created a secondary founder effect within M that decreased the overall age estimates. Such a scenario would impact even more on ρ than ML estimates, which is indeed what we see (Table 1). An expansion 45–35 ka would also fit well with the palaeoenvironmental and archaeological evidence [2, 67, 68], and is further supported by an increment in *N*_*e*_ associated with M across South Asia from ~40 ka (Additional file 1: Figure S1).

The next major discernible signal in indigenous lineages begins ~12 ka, at the Pleistocene/Holocene transition. Various star-like clades dating 12–9 ka suggest a rapid expansion across the Subcontinent, namely M6a1a (11.4 ka), M18a (9.2 ka), M30d (12.1 ka), R8b1 (11.6 ka) and U2b2 (9.2 ka), all from a southern source; and R30c + 373 (12.4 ka), from the west. An increment in *N*_*e*_ is also observed at this time in the BSP for haplogroup M in the west and south (Additional file 1: Figure S1).

We also see a further increment in the last few millennia. BSPs for M in the west and centre show an increment in the last 2.5 ka (Additional file 1: Figure S1), associated with the emergence of several subclades in the west (M2a3a + 4314, M2a1b, M2c + 1888 + 146, M30a2, M5a3b, M6a1 + 5585 + 146 + 1508) and centre (M2a1a1b, M3b, M3a1a, M63, M5a2a2 + 234, M5a3a and M61a + 5294).

### West Eurasian mtDNA lineages in South Asia: Multiple dispersals from the northwest since the LGM

Prehistoric West Eurasian lineages make up almost 20% of the South Asian genetic pool overall.

#### LGM and Late Glacial arrivals

The earliest genetic evidence of movements into the Subcontinent after the first settlement is seen in haplogroup N1a1b1, which dates to ~21 ka (Additional file 1: Figure S2), with a probable source in the Near East [69]. Other haplogroups with similar age estimates and a Near Eastern source (pre-HV2, HV + 146!, HV + 9716, HV + 73!, pre-U1c, U1a1, J1d and a basal clade within T2) may have moved eastwards in the same time frame (Table 1, Additional file 1: Figure S2), corresponding to 2.6% in the overall South Asian 1KGP data. Further Near Eastern clades (W4, HV + 16311!, HV12b, I1, U7a and J1b1b1) spread to South Asia in the Late Glacial period, 16–13 ka (Table 1, Additional file 1: Figure S2), with frequencies of 4.5% in the South Asian 1KGP data.

#### Early postglacial arrivals

At ~12 ka, when various indigenous lineages show signals of expansion, we also observe further lineages arriving from Southwest Asia with exclusively South Asian branches (T2e2, T2 + 195 + 4225, W3a1 + 143, W3a1b, U1a3 + 10253, N1a2, U7a + 12373 and U7a3a + 6150) (Table 1, Additional file 1: Figure S2). Furthermore, South Asian lineages are nested within numerous other branches with similar node age estimates (W6, T2b, T2d1a, U7b + 16309! and K1a1b2a), allowing us to circumscribe the arrival times (Table 1, Additional file 1: Figure S2). These lineages represent a frequency of 4.7% in the South Asian 1KGP dataset.

#### Neolithic arrivals

More lineages entered the Subcontinent ~9–5 ka, representing putative Neolithic markers with a distinct origin in Anatolia, the Caucasus and Iran, again harbouring distinctive nested South Asian subclades (K2a5 + 2831 + 189, HV14 + 150, H13a2a + 8952, K2a5 + 2831, *X*2 + 153! + 7109 and U1a3a) (Table 1, Additional file 1: Figure S2) (3.4%). There is also evidence of movements from the Arabian Peninsula/Near East; the branch R0a2 + 11152 (~7.1 ka) is the most striking example. One case, H2b, might trace its source to Eastern Europe and may have entered South Asia through Central Asia a little later, as we discuss below.

#### Bronze Age arrivals

**Fig. 2:**
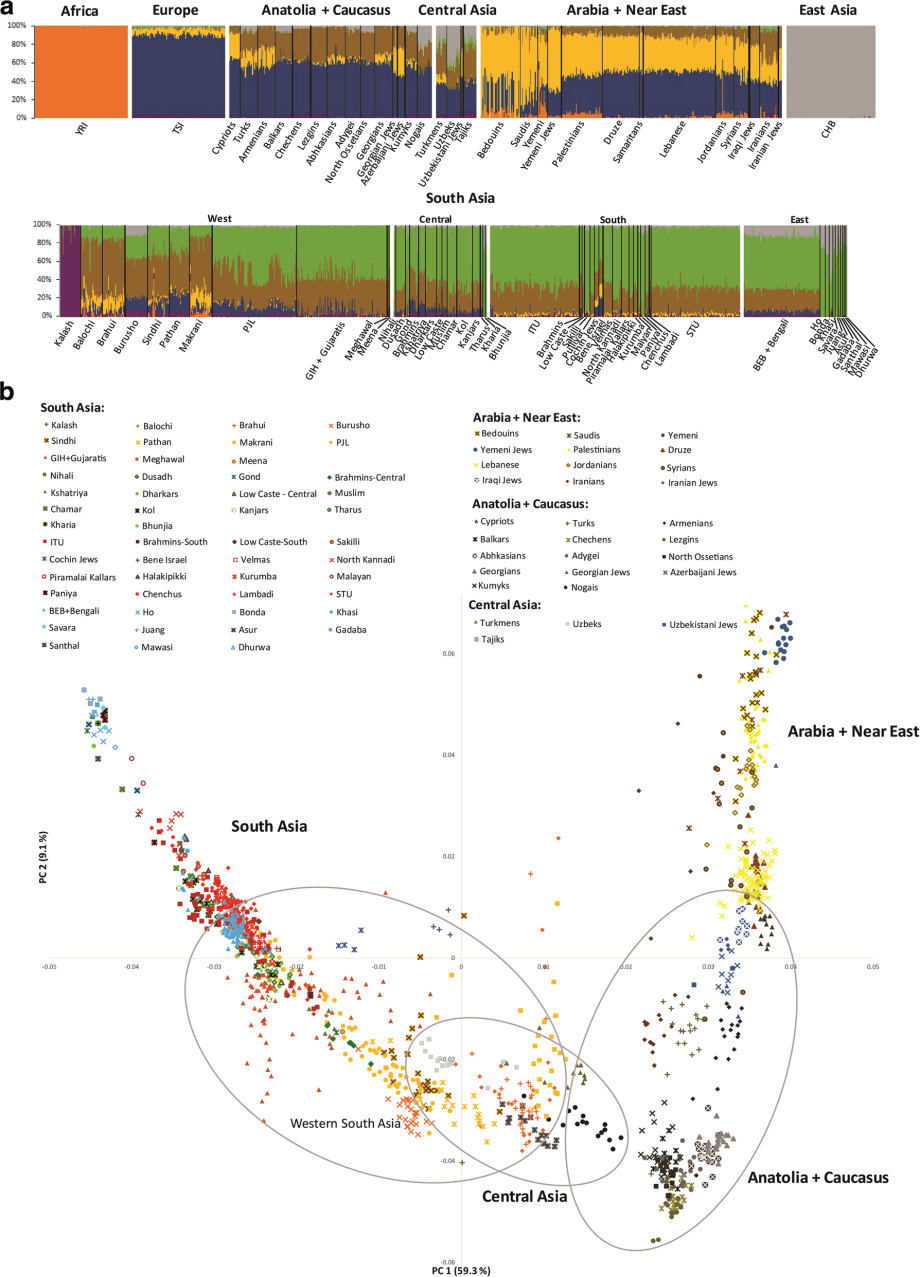
**a** ADMIXTURE analysis for *K* = 7. **b** PCA of South Asian populations. Detailed information on the populations included in the Additional file 1: Table S3. Note that the three typical European components are not detected here in the Tuscans, probably due to the small overall European representation in the analysis

In the last 4 ka, most genetic influx on the maternal line was restricted to Pakistan and traces mostly to Iran (H29 + 9156 + 4689, R2a + 7142 and U1a1a2a) (2.4% in South Asia, reaching 5.4% in the western populations). Gene flow at this time was clearly bi-directional, as seen in the expansion west of lineages M5a2a4, U2c1b + 146 and M3a1b + 13105). This is reflected in the genome-wide ADMIXTURE analysis (below), where the autochthonous South Asian component (green in Fig. 2a) appears at low levels in Iran. As an aside, the bulk of Romani lineages belongs to the branch M5a1b1a1 [70] at 3.0 ka, supporting previous linguistic and genetic evidence for a South Asian origin for the Romani diaspora [70, 71] in the west of the Subcontinent.

### GW overview of South Asia

South Asian populations can be distinguished in both the ADMIXTURE and sNMF analyses from *K* = 3 (Additional file 1: Figure S3 and Figure S4), highlighting the distinctive genetic diversity of the region. At the highest likelihood value of *K* = 7 (Fig. 2a and Additional file 1: Figure S5*a*), the overall pattern is straightforward and clinal [72], with a substantial autochthonous component (shown in green) across the region, apart from the Kalash, which display a virtually exclusive component probably caused by localised genetic drift in a small, isolated population [72, 73].

A striking feature in both the ADMXTURE and sNMF analyses (for *K* = 7) is the much higher fraction of West Eurasian components (brown, yellow and dark blue) in the western (especially Pakistani) South Asian populations. The main non-autochthonous component in the Subcontinent, the Iran/Caucasus/Steppe component (brown), exceeds 35% in Pakistan and Gujarat [23–25], although it reaches most of the Subcontinent. This component approaches ~100% in Late Palaeolithic and Mesolithic remains from the Caucasus, and was therefore dubbed the “Caucasus hunter–gatherer” (CHG) component [74], but it is seen at similarly high frequencies in remains from Mesolithic and Neolithic Iran [75] and at ~50% in Early Bronze Age Yamnaya pastoralist remains from the Pontic-Caspian steppe [53, 76], as shown in Additional file 1: Figure S6 for *K* = 7 (lowest cross-validation error, Additional file 1: Figure S5*b*).

The Pakistani Muslim Balochi, Brahui and Makrani carry ~15% of the Near Eastern/Arabian component (yellow), which is carried across Europe with the spread of the Early Neolithic [75, 77]. However, this component is virtually absent in other South Asians (including Muslims) except for Jewish groups (supporting previous mtDNA evidence for little genetic input from Arabia into Indian Muslim populations [78]).

The PCA (Fig. 2b) portrays a complex gradient of affinities, but with South Asians closer to Central Asian and Caucasus groups than to those from the Near East or Arabia. Pakistani populations occupy an intermediate position, particularly close to the currently Turkic-speaking peoples of Central Asia (the Turkmens, the Nogais and the Uzbeks) and the Indo-Iranian-speaking Tajiks in PC1 (which accounts for 59.3% of the variation). Genetically, Turkic-speaking groups resemble their geographic neighbours, indicating deep local ancestry and recent language shift [79].

The current paradigm for explaining modern Indian population structure suggests that they derive from admixture between two main ancestral populations, Ancient North Indians (ANI) and Ancient South Indians (ASI) [25], with the proximity of Pakistani groups and Gujaratis to Southwest Asians due to high levels of ANI ancestry [25], which my have arrived in two waves [24]. However, our mtDNA results (and the current GW analysis) suggest that the process is likely to have been much more complex. The profile for Pakistani populations is likely the result of at least four waves of dispersal into the region, involving all three of the inferred ancestral West Eurasian components, from at least as far back as the LGM through into the Bronze Age.

The Yamnaya aDNA samples are scattered around the Central Asian and Pakistani groups (Additional file 1: Figure S8), confirming the ADMIXTURE results (Additional file 1: Figure S6), and suggesting links between the Bronze Age Steppe and today’s Central Asia and Indian Subcontinent. Pakistanis and Gujaratis appear much more scattered in PC1 than other South Asians, which only show substantial divergence in the lower-weight PC2 (9.1%) and PC3 (6.3%) (Fig. 2b, Additional file 1: Figure S7).

### Comparing marker systems: Massively different ancestry on the male and female lines of descent

The mtDNA patterns suggest much higher levels of autochthonous variation on the maternal line (~70–90%) compared to the overall GW estimate (about a half to two-thirds), the implications of which we further explored by studying Y-chromosome lineages. We used the five South Asian 1KGP populations, which comprise unbiased population data, and are the only available datasets that can be simultaneously analysed for GW, mtDNA and Y-chromosome variation.

**Fig. 3:**
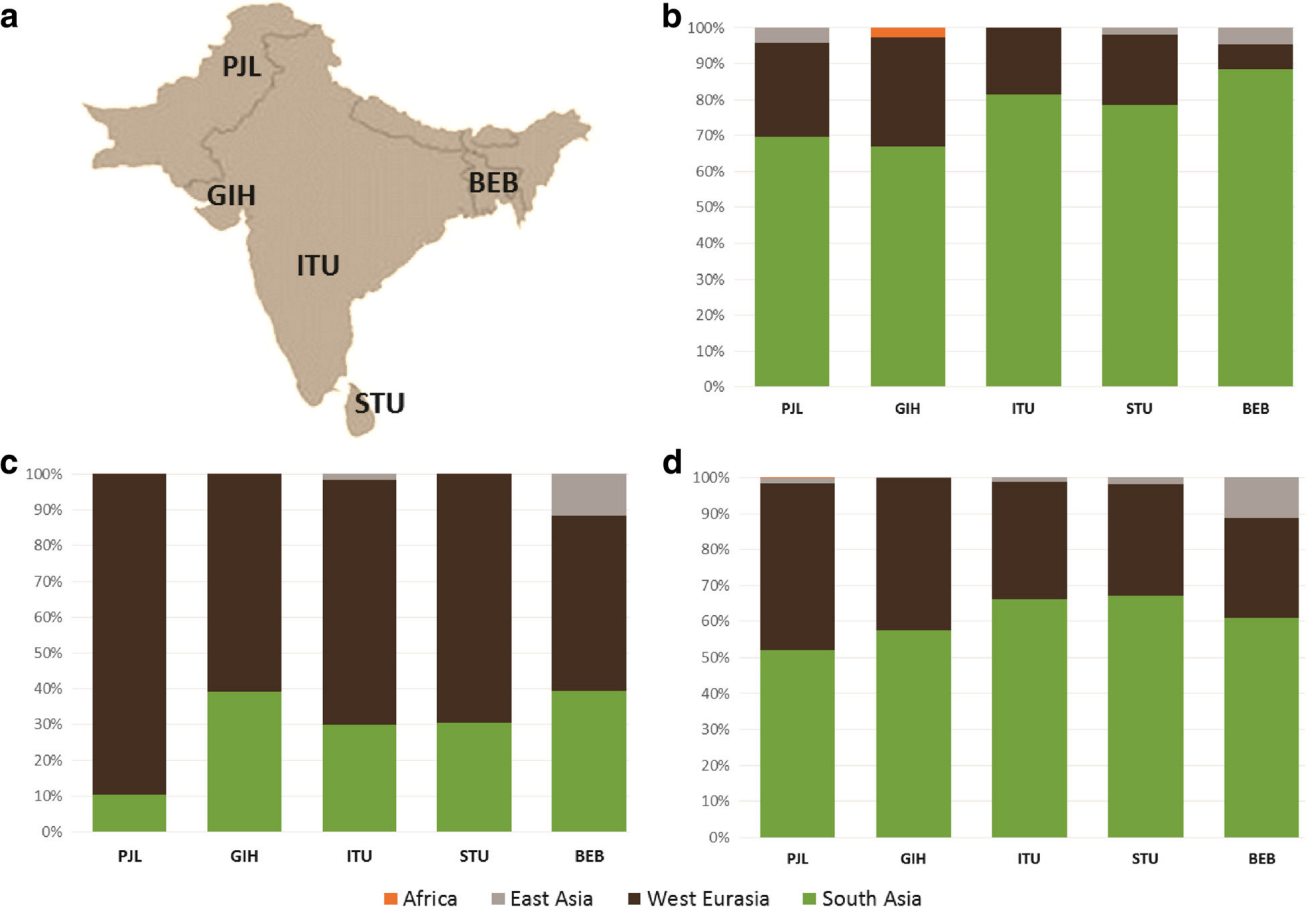
The ancestry of South Asian 1KGP populations according to different molecular markers: **a** sampling locations, **b** mtDNA lineages, **c** Y-chromosome lineages and **d** GW components (based on ADMIXTURE, *K* = 7). Putative origin of the uniparental lineages present in the populations in the Additional file 1; Table S4. Population codes: PJL—Punjabi from Lahore, Pakistan; GIH—Gujarati Indian from Houston, Texas; ITU—Indian Telugu from the UK; STU—Sri Lankan Tamil from the UK; BEB—Bengali from Bangladesh

A markedly higher proportion of male lineages of likely West Eurasian origin, of ~50–90%, is evident across the Subcontinent (Fig. 3c), in comparison with both the maternal line (Fig. 3b) and the GW pattern (Fig. 3d). A sex-biased pattern is also seen in the East Asian fraction, but is much less marked, with a much lower contribution overall and mainly focused on speakers of Tibeto-Burman and Austroasiatic language families [22].

## Discussion

### Towards a more fine-grained history of South Asian settlement

The phylogeographic analysis of non-recombining marker systems offers certain strengths that can complement genome-wide analyses. In particular, the polarity of gene trees allows us to identify the source of dispersals, and the increasing precision of molecular clocks for mtDNA and the Y chromosome allows us to date events during the ancestry of lineages with some confidence. However, the contribution of the two systems to the overall picture is not always the same, and South Asia is a case in point. Here it is clear from our analyses that there is a very strong sex bias in the ancestry of South Asians. The female line of descent is mostly autochthonous and traces back to the first settlement ~55 ka. However, the male line of descent emphasizes more recent ancestry, since the LGM, from Southwest Asia and Central Asia.

**Fig. 4:**
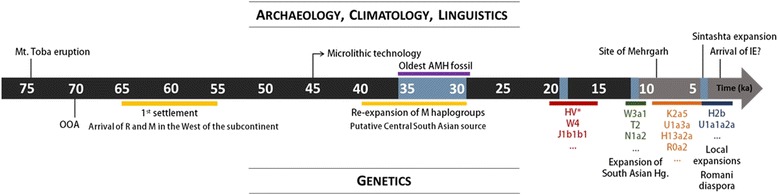
Timeline for AMH evolution in South Asia based on genetic, archaeological, climatological and linguistic evidence. Black and grey portions of the arrow represent Pleistocene and Holocene, respectively. Blue sections correspond to periods of climate changes: dryer periods between 35 and 30 ka, Last Glacial Maximum ~18 ka, Younger Dryas ~12 ka and the “4.2 ka” event. Lineages in red stand for the putative Late Glacial/postglacial genetic influx from West Eurasia; green for migrations from West Eurasia around the Pleistocene/Holocene transition, orange for the Neolithic period and blue for the genetic events in the last 4 ka

The mtDNA is, therefore, at present a uniquely powerful tool for teasing out multiple settlement episodes and dating them, establishing a timeline for demographic events in South Asia. By combining that information with GW patterns and Y-chromosome data, and taking into account also archaeological, palaeontological and palaeoclimatological data, we can reconstruct an outline demographic history of human populations in South Asia that captures some of the complexity of the region and moves beyond simplistic models of admixture between autochthonous Indians and invading Neolithic farmers or Indo-Aryan speakers (Fig. 4).

### Resolving the Pleistocene modern human settlement

Evidence is mounting that haplogroups M, N and R had a common origin and entered South Asia together, following a southern coastal route from Eastern Africa after the Toba eruption [2, 3]. This is supported by their global (non-African) distribution [3], including the detection of basal M lineages, M0 and M1, in Europe and the Near East respectively [65, 80, 81], and their similarity in age elsewhere either using both a stipulated clock [30] and aDNA-driven estimation [65].

We have resolved the issue of the anomalously low age of haplogroup M in South Asia by showing that the discrepancy vanishes when we take into account the regional origin of each basal branch. In the west, M dates to 55.3 [45.1; 65.9] ka, overlapping with the founder age of R (Fig. 4). The anomaly is most likely a result of major expansions across the Subcontinent ~45–35 ka: there is an increment in *N*_*e*_ in M across the Subcontinent ~40 ka, coinciding with the appearance and spread of microlithic technology and greater aridity [67, 68]. The lower age of M is most striking in central India, which is also the centre of gravity of the dramatic radiation of M4’67, which dates to ~40 ka. Microlithic technology can be traced to ~45 ka in central India [82], supporting this region as the likely source of the re-expansion.

### Re-peopling after the Last Glacial Maximum

Although South Asia displays a very high level of indigenous variation, the region subsequently received substantial genetic input from both west and east, dramatically re-shaping its genetic structure. Broadly, South Asian populations are closer to the Caucasus and Central Asian groups rather than to other West Eurasian populations. Pakistanis and Gujaratis in particular carry a preponderance of the “Ancestral North Indian” (ANI) gene pool, contrasting with the ASI or autochthonous population of the Subcontinent [25, 26]. However, our results suggest that this profile is due to multiple dispersals from the north-west, from several distinct sources, rather than just one or two major admixture events in the Neolithic/Bronze Age.

In fact, we see mtDNA lineages from Southwest Asia start to arrive as early as ~20 ka. This was a time of short-lived relative global warmth following the peak of the last glaciation, which might have triggered population movements in several regions [83]. Some lineages arrived in Late Glacial times, again from a Southwest Asian refugium, mirroring the situation in Europe [84]. After ~12 ka, with the end of the Younger Dryas glacial relapse, these movements intensified, with the arrival of yet more Southwest Asian lineages. This period also witnessed the expansion of several autochthonous mtDNA lineages across South Asia, in part from sources in the west (possibly carried alongside dispersing Southwest Asian lineages), but primarily from the south. Supporting this view, *N*_*e*_ increments at this period are visible in the west and the south, related to the expansion of indigenous M lineages.

### Disentangling Early Neolithic and Bronze Age dispersals into South Asia

After the first settlement, most attention in genetic studies has been focused on the Neolithic and Bronze Age periods, in part due to potential implications for the spread of Indo-European languages. The earliest Neolithic sites, on the Indus Valley around Mehrgarh in Baluchistan, date to before 9 ka [85, 86], and the earliest crops in South Asia derived from Southwest Asian founder crops from the Fertile Crescent [19, 87]. Numerous mtDNA lineages entered South Asia in this period from Anatolia, the Caucasus and Iran.

Although some have argued for co-dispersal of the Indo-Aryan languages with the earliest Neolithic from the Fertile Crescent [88, 89], others have argued that, if any language family dispersed with the Neolithic into South Asia, it was more likely to have been the Dravidian family now spoken across much of central and southern India [12]. Moreover, despite a largely imported suite of Near Eastern domesticates, there was also an indigenous component at Mehrgarh, including zebu cattle [85, 86, 90]. The more widely accepted “Steppe hypothesis” [91, 92] for the origins of Indo-European has recently received powerful support from aDNA evidence. Genome-wide, Y-chromosome and mtDNA analyses all suggest Late Neolithic dispersals into Europe, potentially originating amongst Indo-European-speaking Yamnaya pastoralists that arose in the Pontic-Caspian Steppe by ~5 ka, with expansions east and later south into Central Asia in the Bronze Age [53, 76, 93–95]. Given the difficulties with deriving the European Corded Ware directly from the Yamnaya [96], a plausible alternative (yet to be directly tested with genetic evidence) is an earlier Steppe origin amongst Copper Age Khavlyn, Srednij Stog and Skelya pastoralists, ~7-5.5 ka, with an infiltration of southeast European Chalcolithic Tripolye communities ~6.4 ka, giving rise to both the Corded Ware and Yamnaya when it broke up ~5.4 ka [12].

An influx of such migrants into South Asia would likely have contributed to the CHG component in the GW analysis found across the Subcontinent, as this is seen at a high rate amongst samples from the putative Yamnaya source pool and descendant Central Asian Bronze Age groups. Archaeological evidence suggests that Middle Bronze Age Andronovo descendants of the Early Bronze Age horse-based, pastoralist and chariot-using Sintashta culture, located in the grasslands and river valleys to the east of the Southern Ural Mountains and likely speaking a proto-Indo-Iranian language, probably expanded east and south into Central Asia by ~3.8 ka. Andronovo groups, and potentially Sintashta groups before them, are thought to have infiltrated and dominated the *soma*-using Bactrian Margiana Archaeological Complex (BMAC) in Turkmenistan/northern Afghanistan by 3.5 ka and possibly as early as 4 ka. The BMAC came into contact with the Indus Valley civilisation in Baluchistan from ~4 ka onwards, around the beginning of the Indus Valley decline, with pastoralist dominated groups dispersing further into South Asia by ~3.5 ka, as well as westwards across northern Iran into Syria (which came under the sway of the Indo-Iranian-speaking Mitanni) and Anatolia [12, 95, 97, 98].

Although GW patterns have been broadly argued to support this view [24], there have also been arguments against. For example, Metspalu et al. [28] argued cogently that the GW pattern in South Asia was the result of a complex series of processes, but they also suggested that an East Asian component, common in extant Central Asians, should be evident in the Subcontinent if it had experienced large-scale Bronze Age immigration from Central Asia. In fact, however, aDNA evidence shows that this element was not present in the relevant source regions in the Early Bronze Age [76]. Moreover, whilst the dating and genealogical resolution of Y-chromosome lineages has been weak until recently, it is now clear that a very large fraction of Y-chromosome variation in South Asia has a recent West Eurasian source.

### Genetic signals of Indo-European expansions

Contrary to earlier studies [99, 100], recent analyses of Y-chromosome sequence data [55, 58, 94] suggest that haplogroup R1a expanded both west and east across Eurasia during the Late Neolithic/Bronze Age. R1a-M17 (R1a-M198 or R1a1a) accounts for 17.5% of male lineages in Indian data overall, and it displays significantly higher frequencies in Indo-European than in Dravidian speakers [55].

**Fig. 5:**
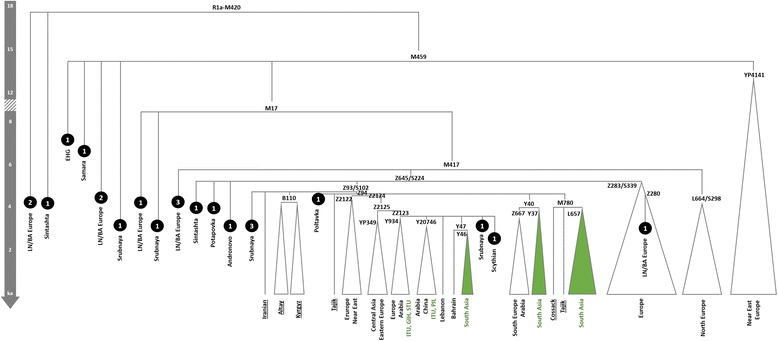
Schematic tree of Y-chromosome haplogroup R1a. Phylogeny and age estimates based on Yfull tree v4.10 [53]. Age estimates are corroborated by published estimates [54] for some nodes and aDNA evidence from radiocarbon and indirectly dated samples. Underlined samples and/or clades from Karmin et al. 2015 [54]. Black circles represent aDNA samples (number represents the sample size for each culture/period; LN/BA stands for Late Neolithic/Bronze Age) [52, 76, 77]

There are now sufficient high-quality Y-chromosome data available (especially Poznik et al. [58]) to be able to draw clear conclusions about the timing and direction of dispersal of R1a (Fig. 5). The indigenous South Asian subclades are too young to signal Early Neolithic dispersals from Iran, and strongly support Bronze Age incursions from Central Asia. The derived R1a-Z93 and the further derived R1a-Z94 subclades harbour the bulk of Central and South Asian R1a lineages [55, 58], as well as including some Russian and European lineages, and have been variously dated to 5.6 [4.0;7.3] ka [55], 4.5–5.3 ka with expansions ~4.0–4.5 ka [58], or 4.7 [4.0;5.5] ka (Yfull tree v4.10 [54]). The South Asian R1a-L657, dated to ~4.2 ka [3.3;5.1] (Yfull tree v4.10 [54]]), is the largest (in the 1KG dataset) of several closely related subclades within R1a-Z94 of very similar time depth. Moreover, not only has R1a been found in all Sintashta and Sintashta-derived Andronovo and Srubnaya remains analysed to date at the genome-wide level (nine in total) [76, 77], and been previously identified in a majority of Andronovo (2/3) and post-Andronovo Iron Age (Tagar and Tachtyk: 6/6) male samples from southern central Siberia tested using microsatellite analysis [101], it has also been identified in other remains across Europe and Central Asia ranging from the Mesolithic up until the Iron Age (Fig. 5).

The other major member of haplogroup R in South Asia, R2, shows a strikingly different pattern. It also has deep non-Subcontinental branches, nesting a South Asian specific subclade. But the deep lineages are mainly seen in the eastern part of the Near East, rather than Central Asia or eastern Europe, and the Subcontinental specific subclade is older, dating to ~8 ka [55].

Altogether, therefore, the recently refined Y-chromosome tree strongly suggests that R1a is indeed a highly plausible marker for the long-contested Bronze Age spread of Indo-Aryan speakers into South Asia, although dated aDNA evidence will be needed for a precise estimate of its arrival in various parts of the Subcontinent. aDNA will also be needed to test the hypothesis that there were several streams of Indo-Aryan immigration (each with a different pantheon), for example with the earliest arriving ~3.4 ka and those following the Rigveda several centuries later [12]. Although they are closely related, suggesting they likely spread from a single Central Asian source pool, there do seem to be at least three and probably more R1a founder clades within the Subcontinent [58], consistent with multiple waves of arrival. Genomic Y-chromosome phylogeography is in its infancy compared to mitogenome analysis so it is of course likely that the picture will evolve with sequencing of further South Asian Y-chromosomes, but the picture is already sufficiently clear that we do not expect it to change drastically.

**Fig. 6:**
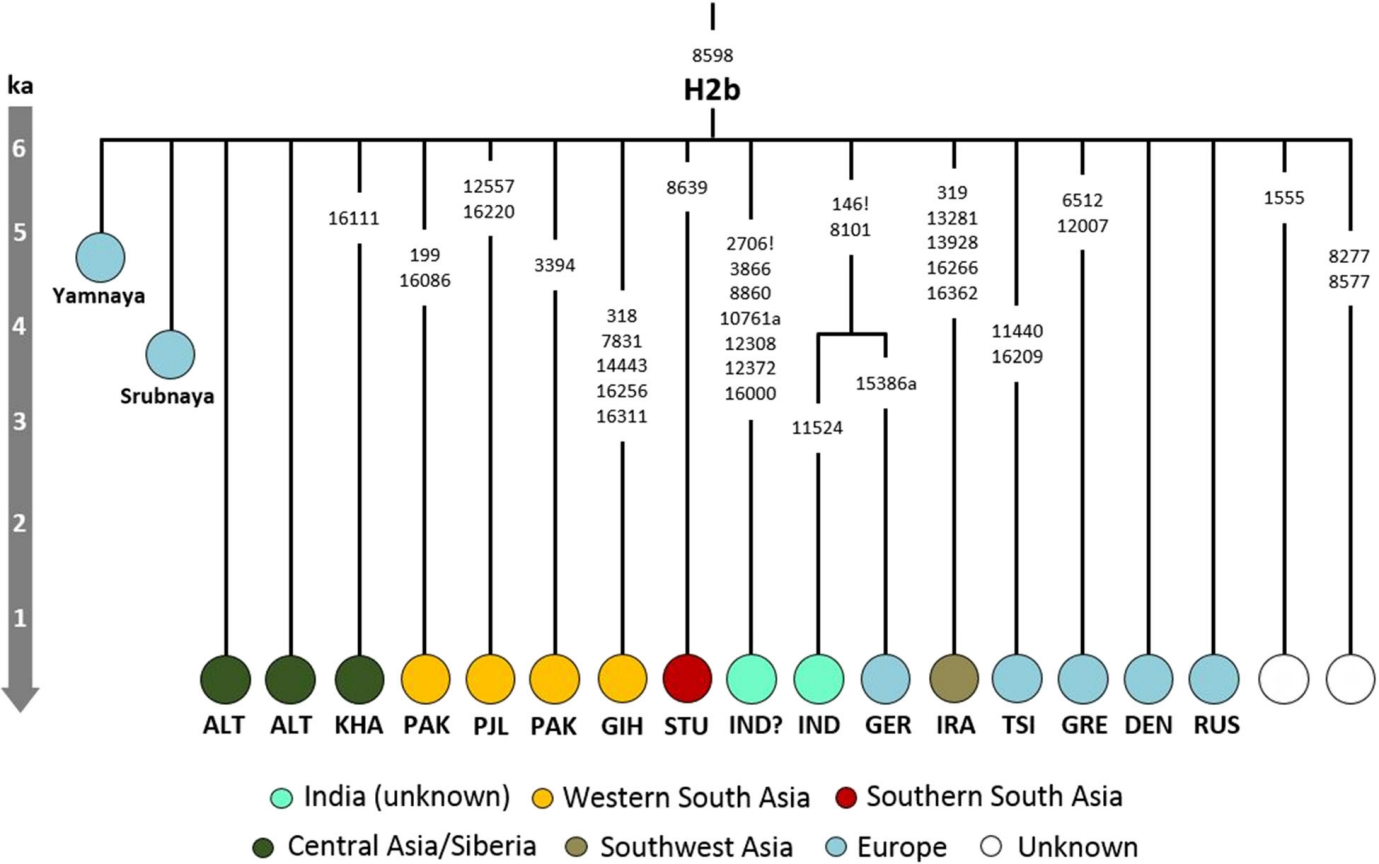
Tree of mtDNA haplogroup H2b based on ML age estimates for modern sequences. Population codes: ALT—Altai, DEN—Denmark, GER—Germany, GIH—Gujarati Indian from Houston, Texas, GRE—Greece, IND—India (without more details regarding location within India; the sample marked with “?” is possibly Indian), IRA—Iraq, KHA—Khamnigan, PAK—Pakistan, PJL—Punjabi from Lahore, Pakistan, RUS—Russia, TSI—Tuscans from Italy (the Additional file 1: Table S2). The ancient Yamnaya sample has been radiocarbon dated to 3010–2622 calibrated years BCE (Before Common Era) [52]; ancient Srubnaya sample dates to 1850–1600 BCE [77]

Although these migrations appear to have been male-driven, it might nevertheless be possible to detect a minor maternal signal. For example, haplogroup H2b (dating to 6.2 ka [3.8–8.7] ka; Fig. 6) is a starlike subclade with a probable ultimate ancestry in Eastern Europe, but includes several South Asian lineages (from Pakistan, India and Sri Lanka) that probably arrived more recently from Central Asia. Tellingly, H2b also includes two aDNA samples (Fig. 6): one individual from the small number of Yamnaya sampled to date [53, 76] and another from the Late Bronze Age Srubnaya culture [77].

Even so, the spread of Indo-European within the Subcontinent seems to have been mainly male-mediated, in agreement with recent X-chromosome analyses [102] and as indicated by the high frequency of West Eurasian (mainly R1a) paternal lineages across the region—varying in the 1KG data from ~25% in the northwest and ~20% in the northeast to ~14% in the south, but much more dramatically when taking caste and language into account (from almost 50% in upper-caste Indo-European speakers to almost zero in eastern Austro-Asiatic speakers) [12, 56, 59]. This present-day distribution cannot be directly correlated with language replacement, however, since the signal is also strong in Dravidian-speaking populations (Fig. 3). The last four millennia witnessed major cultural changes in the Indian Subcontinent, with the decline of the Indus Valley civilisation and the rise of Vedic religion, based on a strict caste system, often associated with the arrival of Indo-Aryan speakers. The mix of autochthonous and immigrant genetic lineages seen across South Asia, however, suggests a gradual merging of male-dominated Andronovo/BMAC immigrants with the indigenous descendants of the Indus Valley civilisation [12], possibly associated with the spread of the Megalithic culture as far south as Sri Lanka in the first century Before Common Era (BCE), prior to the establishment of the full jāti caste system very roughly ~2 ka [12, 103]. Basu et al. [26] date the “freezing” of India’s population structure to ~1.5 ka.

Although the mtDNA does not suggest similar continent-wide dispersals involving women, the last ~4 ka nevertheless witnessed a profound impact on the demography of maternal lineages, with a population increment associated with the indigenous lineages which might have involved local movements and facilitated the diffusion of the Indo-Aryan languages. This expansion is mainly evident amongst the autochthonous lineages in west and central South Asia.

We see no evidence that the caste system emerged in the wake of the arrival of Indo-Aryan speakers from the north, in agreement with formal admixture analyses [24, 26]. Higher-ranking castes do seem closer genetically to Pakistan and ultimately Caucasus and Central Asian populations, but this proximity was most likely established over millennia, by several distinct migratory events—indeed, a sizeable fraction of the non-R1a West Eurasian Y-chromosome lineages (e.g. R2a-M124, J2-M241, L1a-M27, L1c-M357) were most likely associated with the spread of agriculture or even earlier expansions from Southwest Asia, as with the mtDNA lineages [55, 59]. The tribal groups are generally more divergent from other South Asian groups and in particular from western South Asians, but the particular genetic diversity of tribal groups might have been due to isolation [20], and not necessarily because of more recent strict social boundaries enforced by newly-arriving groups imposing a new system, which in its historical form was likely established much more recently, not more than around 2000 years ago [12, 24, 26, 103].

## Conclusions

### The trans-continental demographic impact of the Eurasian Bronze Age

In conclusion, analysis of the uniparental marker systems can provide complementary insight into the main genome-wide component that arrived in and spread throughout South Asia since the LGM. This “CHG” component is now known to reach almost 100% in both pre-Neolithic remains from the Caucasus [74] and pre-Neolithic and Early Neolithic remains from Iran [75], and to occur at ~50% in the Pontic-Caspian steppe zone [53, 76], north of the Caucasus, by ~5 ka. This component underwent of multiple dispersals into the Subcontinent, with chronologically distinct sources in the eastern Fertile Crescent and the Steppe, via Central Asia. Moreover, these dispersals involved not simply the spread of early farming from Southwest Asia and the male-dominated arrival of Indo-Aryan speakers from Central Asia. The mtDNA signal suggests several streams of dispersal into the Subcontinent from the northwest since the LGM, and there were also more recent dispersals from the east, with a more limited impact [22].

In some ways, the overall picture for South Asia resembles the settlement history for a much smaller peninsula on the far side of the Near East with a similar sink status—Europe. Europe too was settled by early modern humans in the late Pleistocene, albeit suffering much greater impact from the LGM due to its latitude. Even so, Europe similarly experienced subsequent settlement episodes from the LGM onwards, culminating in the spread of agriculture from Southwest Asia ~9 ka, followed by the similarly male-dominated spread of pastoralism and, most likely, the Indo-European language family in the Late Neolithic/Early Bronze Age from the Pontic-Caspian steppe [65, 76, 77, 84, 104, 105].

Indeed, Y-chromosome haplogroup R1a, which spread with pastoralism and the Indo-European languages into South Asia, also seems to have been carried into Europe a millennium earlier, alongside a similar pastoral economy and language package and its sibling lineage, R1b [53, 58, 76, 94]. Notably, however, the extent to which the R1 lineages replaced earlier Y chromosomes was much greater across Europe than we see in South Asia. This corresponds to the greater impact of Indo-European languages in Europe, which ultimately left few relicts of earlier language families surviving—the only cases still extant being Basque and Finno-Ugric, with Etruscan and Iberian as well-attested but extinct examples. By comparison, almost a quarter of modern Indians speak the Dravidian languages that seem most likely to have been spread by the first farmers [12].

This greater impact in Europe is also reflected in the genome-wide picture. In Europe, although the CHG component is only 10–15% in most populations, it is thought to have been accompanied by a similar fraction of indigenous Mesolithic European lineages from the steppe, seen in Yamnaya samples [53]. This component does not seem to have spread significantly east and south into Central and South Asia, however [76].

Furthermore, in the case of Europe, the major stages are simpler to disentangle from the genome-wide evidence. This is because the distinctiveness of the Levantine source for the Early Neolithic, compared to the Pontic-Caspian steppe, gives most European populations a clear tripartite ancestry that is less evident in South Asia. In fact, even in Europe the situation may be more complex than it first appeared [80, 105, 106]. In the Subcontinent, the Levantine component is (like the European Mesolithic component) minor, due to a deep east–west separation across the Fertile Crescent prior to the spread of the Neolithic [75]. As a result, both the Southwest Asian source for the Late Palaeolithic/Early Holocene and the Steppe/Central Asian source for the Bronze Age largely share the same ancestral pool, which may have arisen in the region of the Caucasus and eastern Fertile Crescent and expanded both north and south during the later Neolithic and Early Bronze Age [74, 75, 95].

Consequently, it may be that only a minor fraction of the CHG component represents Indo-Aryan arrivals in South Asia, perhaps helping to explain why Metspalu et al. [28] were unable to detect it. In any case, estimates of the putative ancestral contributions in clustering analyses such as ADMIXTURE vary considerably depending on the data used, as well as being confounded by other factors such as bottlenecks and unsampled source regions, and so need to be treated with considerable caution [107, 108].

However, an attempt to quantify the relative contribution of Iran/Caucasus *versus* the Steppe by formal admixture analyses was recently made by Lazaridis et al. [75], using ancient DNA data to identify Neolithic Iran and the Yamnaya as the most plausible sources. Like Y-chromosome evidence, this analysis has again emphasized a lack of a direct fit with modern languages—for example, the Iranian component contributes predominantly in several sampled populations in the northwest, both Iranian and Dravidian speaking. Even so, in most of the sampled populations, the Steppe contribution was estimated to equal or even exceed the Iranian fraction, in agreement with the picture from uniparental markers presented here.

## Additional files

Additional file 1: Figure S1. BSP for haplogroup M in different regions of the Subcontinent: (a) West, (b) South, (c) Central and (d) East South Asia. **Figure S2.** Putative origin and age ranges (95% confidence interval) for non-autochthonous mtDNA lineages found in South Asia. The colours represent the most likely source for each lineage; branches exclusively with South Asian complete sequences coloured in green, whereas branches that also harbour sequences from other regions in white and with green contour. Ages according to ML estimates. **Figure S3.** ADMIXTURE analysis for all *K* values. Information on the populations included in Additional file 1: Table S3. **Figure S4.** sNMF analysis of modern populations for all *K* values. Information on the populations included in Additional file 1: Table S3. **Figure S5.** Cross-validation errors for different values of *K* for ADMIXTURE: (a) considering only modern populations and (b) including the Yamnaya in the analysis. **Figure S6.** ADMIXTURE analysis including the Yamnaya for all *K* values. Information on the populations included in Additional file 1: Table S3. **Figure S7.** PCA (for PC1 and PC3) of modern populations. Detailed information on the populations included in Additional file 1: Table S3. **Figure S8.** PCA (for PC1 and PC2) including the Yamnaya. Information on the populations included in Additional file 1: Table S3. **Table S1.** List of complete mtDNA sequences belonging to South Asian autochthonous haplogroups. **Table S2.** List of non-autochthonous complete modern mtDNA sequences used in our analyses. **Table S3.** Dataset used for the GW analyses. (a) Modern dataset. Populations marked with three asterisks (***) were added to the dataset exclusively for ADMIXTURE and sNMF runs, thereby being absent from the PCA. **Table S4.** Putative origin for the uniparental lineages found in the 1KGP South Asian populations. (PDF 7577 kb)

Additional file 2: Phylogenetic tree of South Asian mtDNA haplogroups. (XLSX 664 kb)
