# Supplementary material for: A genetic chronology for the Indian Subcontinent points to heavily sex-biased dispersals
Source: BMC Evol Biol. 2017 Mar 23;17:88. doi: 10.1186/s12862-017-0936-9 (PMC5364613; doi:10.1186/s12862-017-0936-9)
Supplement: Supplementary file 1 — BSP for haplogroup M in different regions of the Subcontinent: (a) West, (b) South, (c) Central and (d) East South Asia. Figure S2. Putative origin and age ranges (95% confidence interval) for non-autochthonous mtDNA lineages found in South Asia. The colours represent the most likely source for each lineage; branches exclusively with South Asian complete sequences coloured in green, whereas branches that also harbour sequences from other regions in white and with green contour. Ages according to ML estimates. Figure S3. ADMIXTURE analysis for all K values. Information on the populations included in Additional file 1: Table S3. Figure S4. sNMF analysis of modern populations for all K values. Information on the populations included in Additional file 1: Table S3. Figure S5. Cross-validation errors for different values of K for ADMIXTURE: (a) considering only modern populations and (b) including the Yamnaya in the analysis. Figure S6. ADMIXTURE analysis including the Yamnaya for all K values. Information on the populations included in Additional file 1: Table S3. Figure S7. PCA (for PC1 and PC3) of modern populations. Detailed information on the populations included in Additional file 1: Table S3. Figure S8. PCA (for PC1 and PC2) including the Yamnaya. Information on the populations included in Additional file 1: Table S3. Table S1. List of complete mtDNA sequences belonging to South Asian autochthonous haplogroups. Table S2. List of non-autochthonous complete modern mtDNA sequences used in our analyses. Table S3. Dataset used for the GW analyses. (a) Modern dataset. Populations marked with three asterisks (***) were added to the dataset exclusively for ADMIXTURE and sNMF runs, thereby being absent from the PCA. Table S4. Putative origin for the uniparental lineages found in the 1KGP South Asian populations. (PDF 7577 kb) [file 12862_2017_936_MOESM1_ESM.pdf]

Additional file 1

A genetic chronology for the Indian Subcontinent points to heavily sex-biased dispersals

Marina Silva[1+], Marisa Oliveira[2,3+], Daniel Vieira[4,5], Andreia Brandão[2,3], Teresa Rito[2,6,7], Joana B. Pereira[2,3], Ross M. Fraser[8,9], Bob Hudson[10], Francesca Gandini[1], Ceiridwen Edwards[1], Maria Pala[1], John Koch[11], James F. Wilson[8,12], Luísa Pereira[2,3], Martin B. Richards[1]*, Pedro Soares[3,5]*

[1] Department of Biological Sciences, School of Applied Sciences, University of Huddersfield, Queensgate, Huddersfield, HD1 3DH, United Kingdom

[2] i3S (Instituto de Investigação e Inovação em Saúde, Universidade do Porto), R. Alfredo Allen 208, 4200-135 Porto, Portugal.

[3] IPATIMUP (Instituto de Patologia e Imunologia Molecular da Universidade do Porto), Rua Júlio Amaral de Carvalho 45, 4200-135 Porto, Portugal

[4] Department of Informatics, University of Minho, Campus de Gualtar, 4710-057 Braga, Portugal

[5] CBMA (Centre of Molecular and Environmental Biology), Department of Biology, University of Minho, Campus de Gualtar, 4710-057 Braga, Portugal

[6] Life and Health Sciences Research Institute (ICVS), School of Health Sciences, University of Minho, Campus de Gualtar, 4710-057 Braga, Portugal

[7] ICVS/3B's - PT Government Associate Laboratory, Braga/Guimarães, Portugal

[8] Centre for Global Health Research, Usher Institute of Population Health Sciences and Informatics, University of Edinburgh, Teviot Place, Edinburgh EH8 9AG, Scotland, UK

[9] Synpromics Ltd, Nine Edinburgh Bioquarter, Edinburgh EH16 4UX, UK

[10] Archaeology Department, University of Sydney, New South Wales NSW 2006, Australia

[11] University of Wales Centre for Advanced Welsh and Celtic Studies, National Library of Wales, Aberystwyth, SY23 3HH, Wales, UK

[12] MRC Human Genetics Unit, Institute of Genetics and Molecular Medicine, University of Edinburgh, Western General Hospital, Edinburgh EH4 2XU, Scotland, UK

[+,*] Both authors contributed equally to this work

Corresponding authors:

Martin B. Richards: m.b.richards@hud.ac.uk; Pedro Soares: pedrosoares@bio.uminho.pt

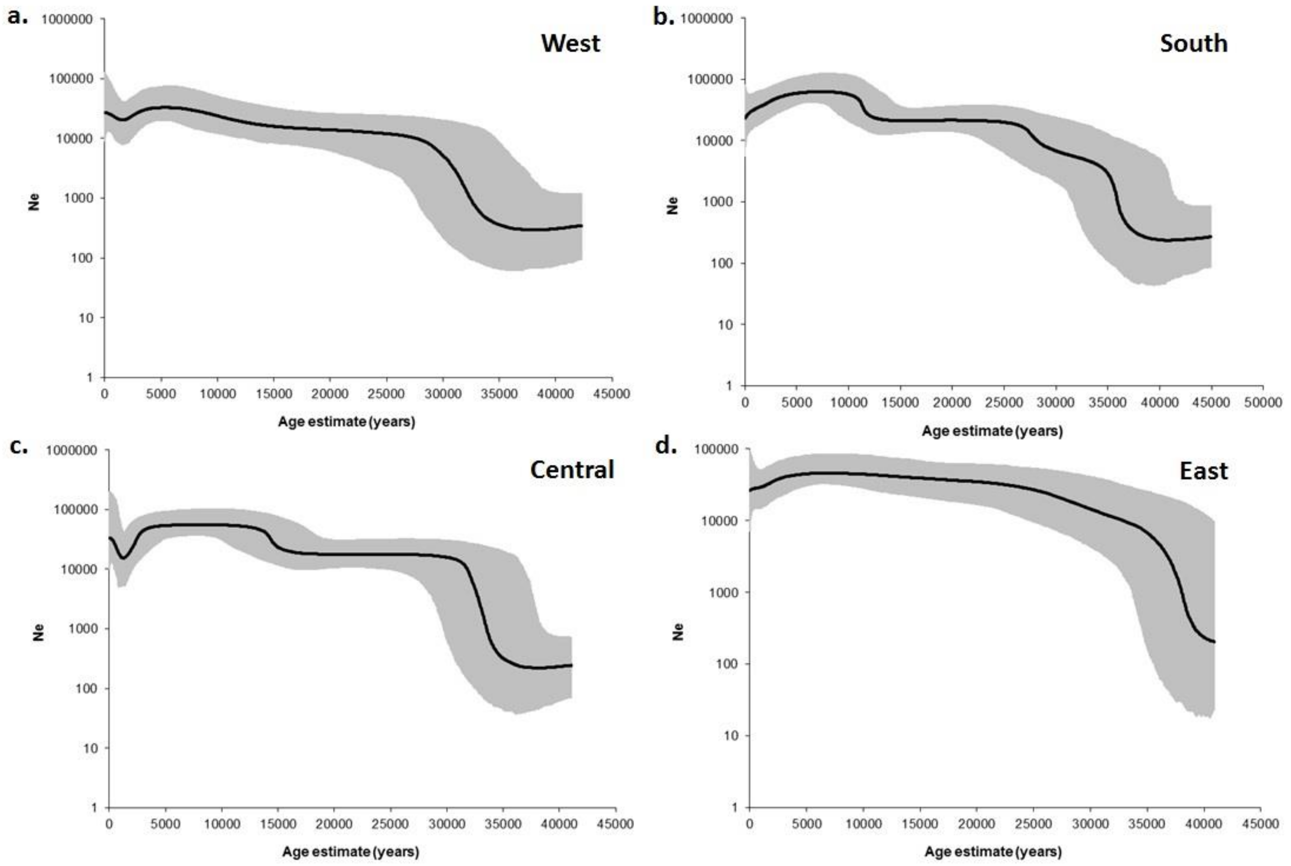

Fig. S1. BSP for haplogroup M in different regions of the subcontinent: (a) West, (b) South, (c) Central and (d) East South Asia.

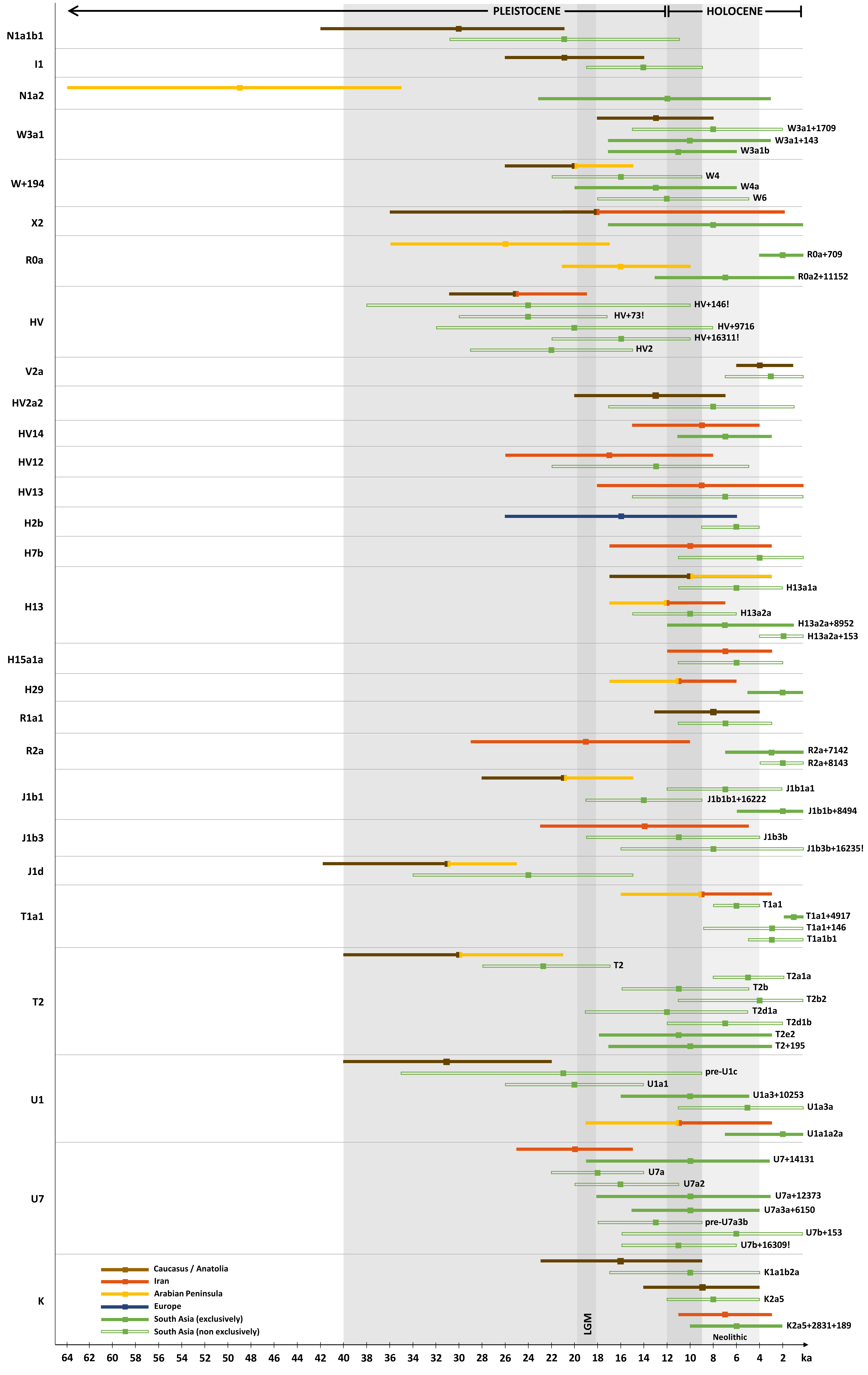
PLEISTOCENE
HOLOCENE
N1a1b1
I1
N1a2
W3a1
W3a1+1709
W3a1+143
W3a1b
W+194
W4
W4a
W6
X2
R0a
R0a+709
R0a2+11152
HV
HV+146!
HV+73!
HV+9716
HV+16311!
HV2
V2a
HV2a2
HV14
HV12
HV13
H2b
H7b
H13
H13a1a
H13a2a
H13a2a+8952
H13a2a+153
H15a1a
H29
R1a1
R2a
R2a+7142
R2a+8143
J1b1
J1b1a1
J1b1b1+16222
J1b1b+8494
J1b3
J1b3b
J1b3b+16235!
J1d
T1a1
T1a1
T1a1+4917
T1a1+146
T1a1b1
T2
T2
T2a1a
T2b
T2b2
T2d1a
T2d1b
T2e2
T2+195
U1
pre-U1c
U1a1
U1a3+10253
U1a3a
U1a1a2a
U7
U7+14131
U7a
U7a2
U7a+12373
U7a3a+6150
pre-U7a3b
U7b+153
U7b+16309!
K
K1a1b2a
K2a5
K2a5+2831+189
Caucasus / Anatolia
Iran
Arabian Peninsula
Europe
South Asia (exclusively)
South Asia (non exclusively)
LGM
Neolithic
64 62 60 58 56 54 52 50 48 46 44 42 40 38 36 34 32 30 28 26 24 22 20 18 16 14 12 10 8 6 4 2 ka

Fig. S2. Putative origin and age ranges (95% confidence interval) for non-autochthonous mtDNA lineages found in South Asia. The colours represent the most likely source for each lineage; branches exclusively with South Asian complete sequences coloured in green, whereas branches that also harbour sequences from other regions in white and with green contour. Ages according to ML estimates.

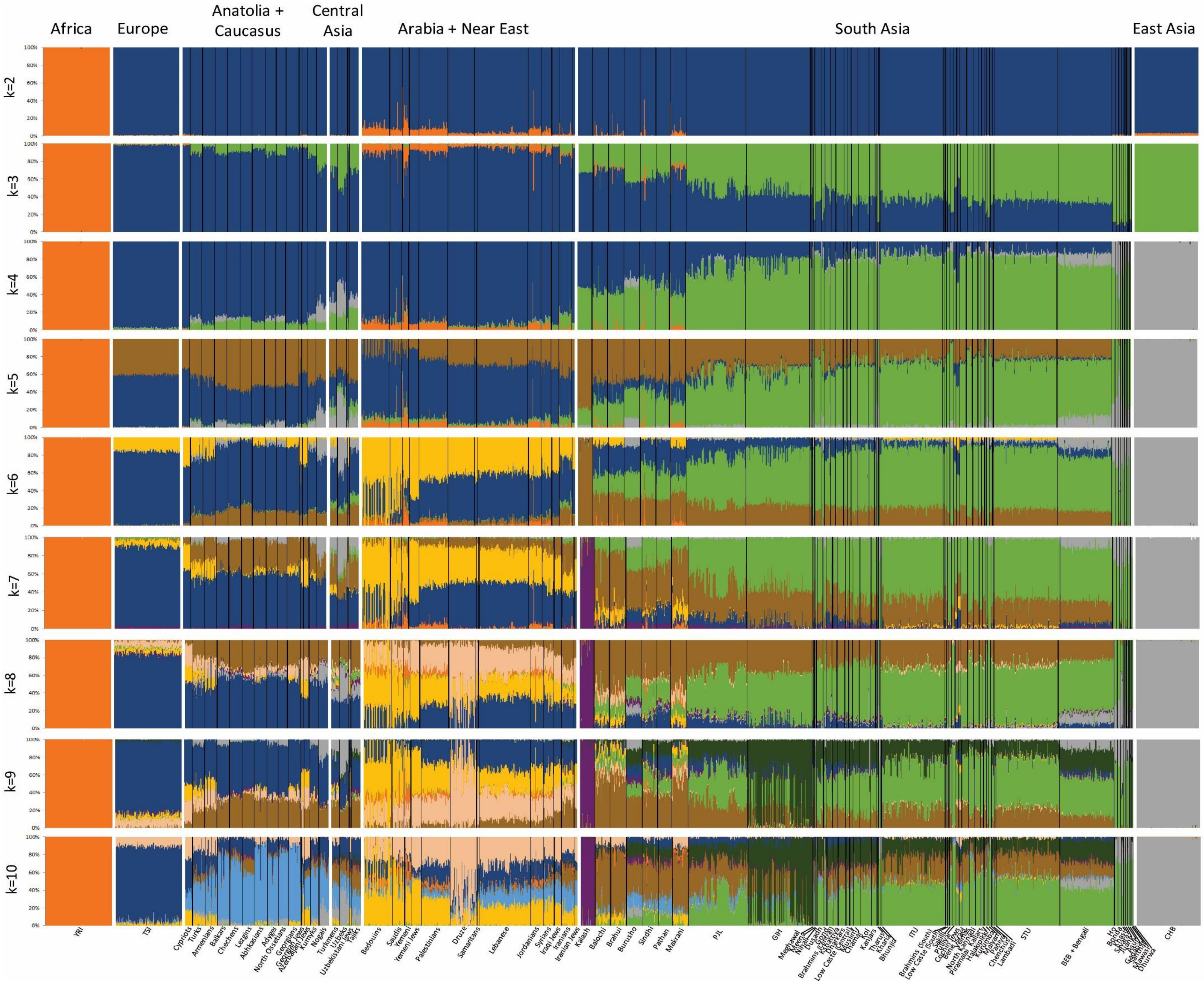

Africa
Europe
Anatolia + Caucasus
Central Asia
Arabia + Near East
South Asia
East Asia
k=2
k=3
k=4
k=5
k=6
k=7
k=8
k=9
k=10
100%
80%
60%
40%
20%
0%
YRI
TSI
Cypriots
Turks
Armenians
Balkars
Chechens
Lezgins
Abhkasians
Adygei
North Ossetians
Georgians
Georgian Jews
Azerbaijani Jews
Kumyks
Nogais
Turkmens
Uzbeks
Uzbekistani Jews
Tajiks
Bedouins
Saudis
Yemeni
Yemeni Jews
Palestinians
Druze
Samaritans
Lebanese
Jordanians
Syrians
Iraqi Jews
Iranians
Iranian Jews
Kalash
Balochi
Brahui
Burusho
Sindhi
Pathan
Makrani
PJL
GIH
Meghawal
Meena
Nihali
Dusadh
Gond
Brahmins (Central)
Kshatriya
Dharkars
Low Caste (Central)
Muslim
Chamar
Kol
Kanjars
Tharus
Kharia
Bhunjia
ITU
Brahmins (South)
Low Caste (South)
Sakilli
Pulliyar
Cochin Jews
Bene Israel
Velamas
North Kannadi
Piramalai Kallars
Halakipikki
Kurumba
Malyan
Paniya
Chenchus
Lambadi
STU
BEB + Bengali
HO
Bonda
Khasi
Savara
Juang
Asur
Gadaba
Santhal
Mawasi
Dhurwa
CHB

Fig.S3. ADMIXTURE analysis for all *K* values. Information on the populations included in Table S3.

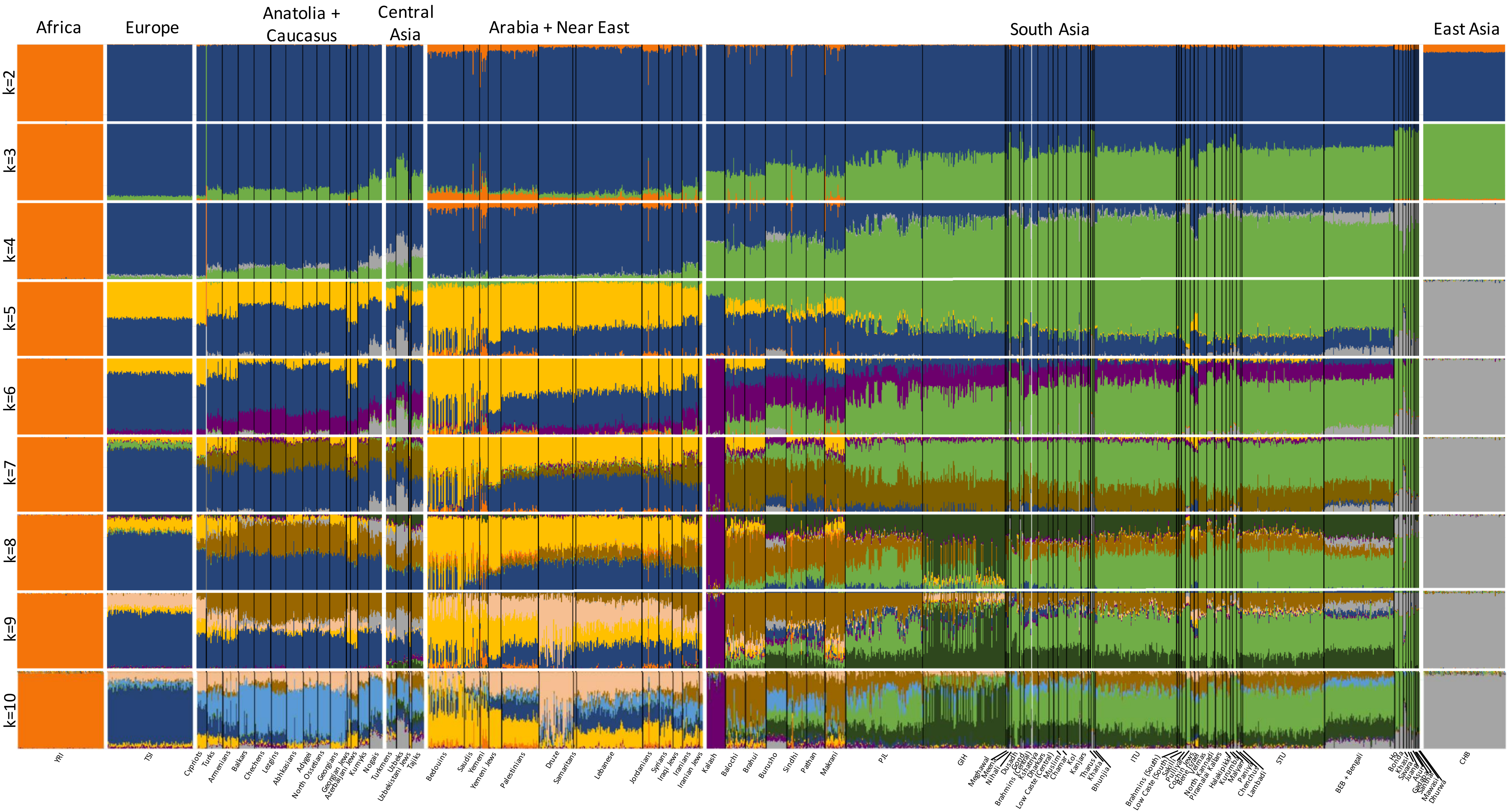

Africa
Europe
Anatolia + Caucasus
Central Asia
Arabia + Near East
South Asia
East Asia
k=2
k=3
k=4
k=5
k=6
k=7
k=8
k=9
k=10
YRI
TSI
Cypriots
Turks
Armenians
Balkars
Chechens
Lezgins
Abhkasians
Adygei
North Ossetians
Georgians
Georgian Jews
Azerbaijani Jews
Kumyks
Nogais
Turkmens
Uzbeks
Uzbekistani Jews
Tajiks
Bedouins
Saudis
Yemeni
Yemeni Jews
Palestinians
Druze
Samaritans
Lebanese
Jordanians
Syrians
Iraqi Jews
Iranians
Iranian Jews
Kalash
Balochi
Brahui
Burusho
Sindhi
Pathan
Makrani
PJL
GIH
Meghawal
Meena
Nihali
Dusadh
Gond
Brahmins (Central)
Kshatriya
Dharkars
Low Caste (Central)
Muslim
Chamar
Kol
Kanjars
Tharus
Kharia
Bhunjia
ITU
Brahmins (South)
Low Caste (South)
Sakilli
Pulliyar
Cochin Jews
Bene Israel
Velmas
North Kannadi
Piramalai Kallars
Halakipikki
Kurumba
Malyan
Paniya
Chenchus
Lambadi
STU
BEB + Bengali
Ho
Bonda
Khasi
Savara
Juang
Asur
Gadaba
Santhal
Mawasi
Dhurwa
CHB

Fig.S4. sNMF analysis of modern populations for all *K* values. Information on the populations included in Table S3.

a.

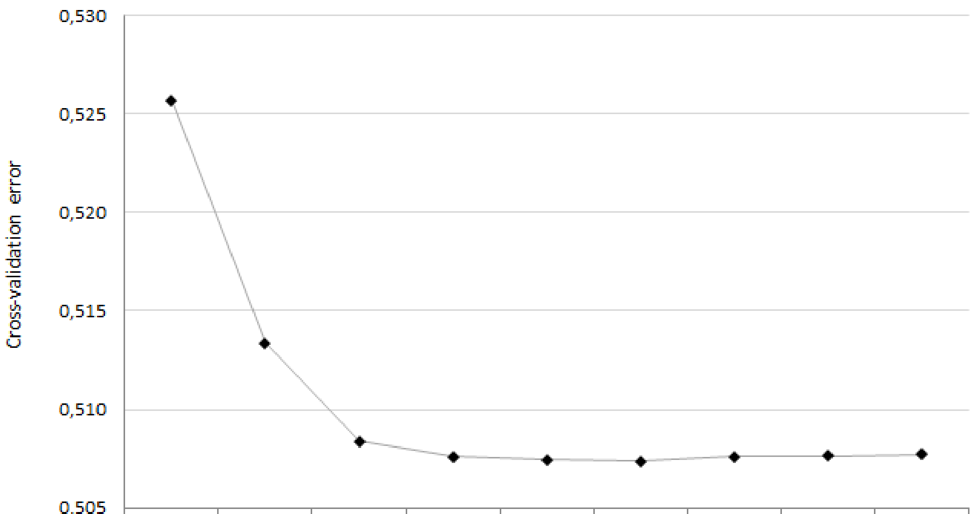

	K=2	K=3	K=4	K=5	K=6	K=7	K=8	K=9	K=10
Modern populations	0,52570	0,51337	0,50838	0,50760	0,50746	0,50738	0,50759	0,50764	0,50773

b.

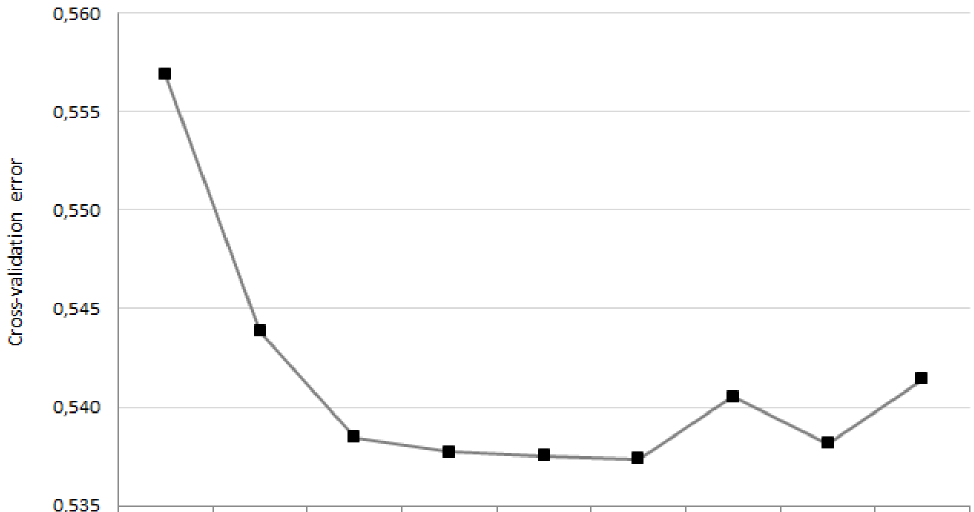

	K=2	K=3	K=4	K=5	K=6	K=7	K=8	K=9	K=10
Modern + Yamnaya	0,55694	0,54383	0,53845	0,53770	0,53753	0,53735	0,54051	0,53814	0,54144

Fig.S5. Cross-validation errors for different values of *K* for ADMIXTURE: (a) considering only modern populations and (b) including the Yamnaya in the analysis.

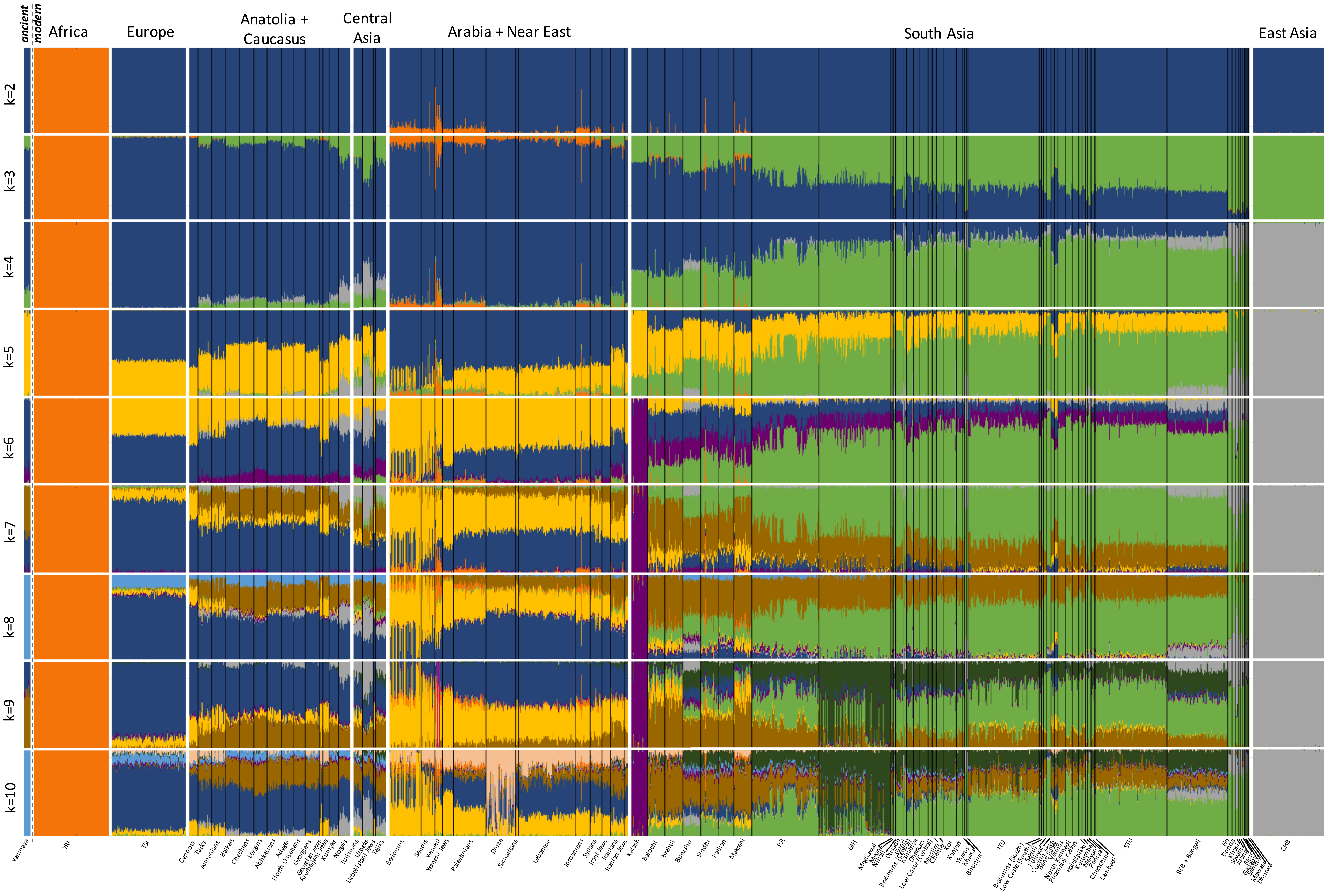
ancient
modern
Africa
Europe
Anatolia + Caucasus
Central Asia
Arabia + Near East
South Asia
East Asia
k=2
k=3
k=4
k=5
k=6
k=7
k=8
k=9
k=10
Yamnaya
YRI
TSI
Cypriots
Turks
Armenians
Balkars
Chechens
Lezgins
Abkhasians
Adygei
North Ossetians
Georgians
Georgian Jews
Azerbaijani Jews
Kumyks
Nogais
Turkmens
Uzbeks
Uzbekistani Jews
Tajiks
Bedouins
Saudis
Yemeni
Yemeni Jews
Palestinians
Druze
Samaritans
Lebanese
Jordanians
Syrians
Iraqi Jews
Iranians
Iranian Jews
Kalash
Balochi
Brahui
Burusho
Sindhi
Pathan
Makrani
PJL
GIH
ITU
STU
BEB + Bengali
CHB

Fig. S6. ADMIXTURE analysis including the Yamnaya for all *K* values. Information on the populations included in Table S3.

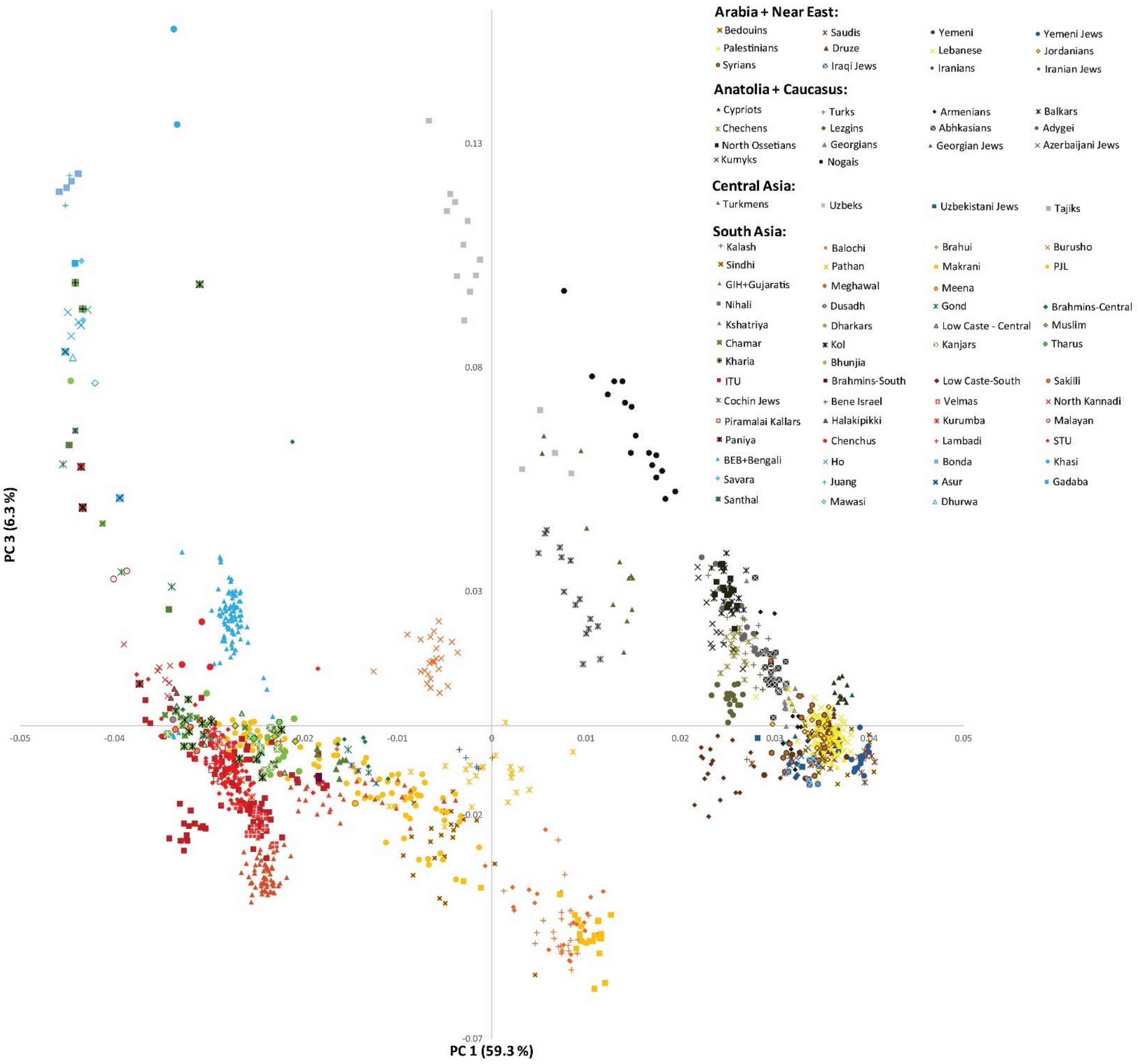

Arabia + Near East:
Bedouins
Saudis
Yemeni
Yemeni Jews
Palestinians
Druze
Lebanese
Jordanians
Syrians
Iraqi Jews
Iranians
Iranian Jews
Anatolia + Caucasus:
Cypriots
Turks
Armenians
Balkars
Chechens
Lezgins
Abhkasians
Adygei
North Ossetians
Georgians
Georgian Jews
Azerbaijani Jews
Kumyks
Nogais
Central Asia:
Turkmens
Uzbeks
Uzbekistani Jews
Tajiks
South Asia:
Kalash
Balochi
Brahui
Burusho
Sindhi
Pathan
Makrani
PJL
GIH+Gujaratis
Meghawal
Meena
Nihali
Dusadh
Gond
Brahmins-Central
Kshatriya
Dharkars
Low Caste - Central
Muslim
Chamar
Kol
Kanjars
Tharus
Kharia
Bhunjia
ITU
Brahmins-South
Low Caste-South
Sakilli
Cochin Jews
Bene Israel
Velmas
North Kannadi
Piramalai Kallars
Halakipikki
Kurumba
Malayan
Paniya
Chenchus
Lambadi
STU
BEB+Bengali
Ho
Bonda
Khasi
Savara
Juang
Asur
Gadaba
Santhal
Mawasi
Dhurwa
PC 3 (6.3 %)
PC 1 (59.3 %)
0.13
0.08
0.03
-0.02
-0.07
-0.05
-0.04
-0.02
-0.01
0
0.01
0.02
0.03
0.04
0.05

Fig. S7. PCA analysis (for PC1 and PC3) of modern populations. Detailed information on the populations included in Table S3.

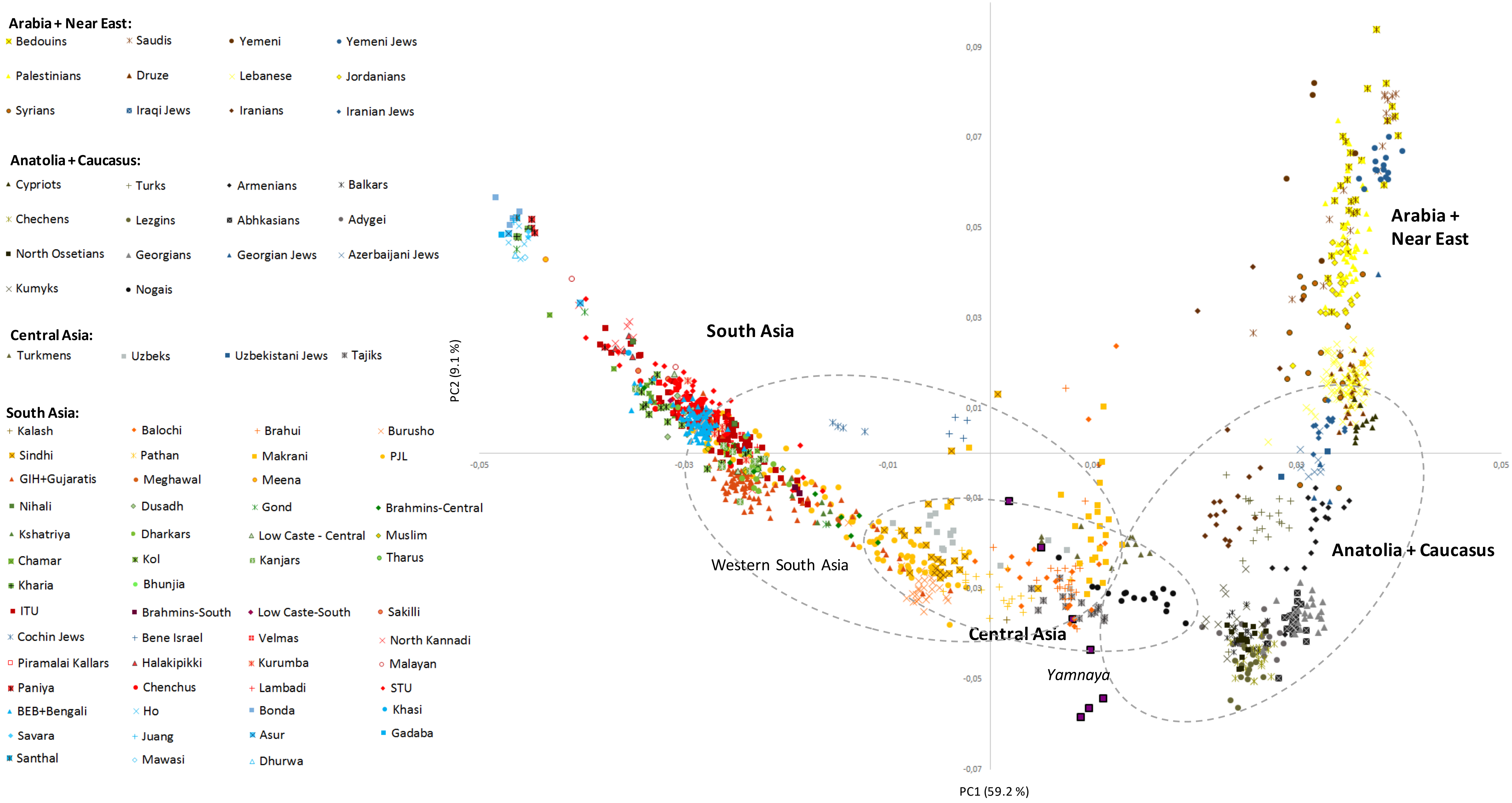

Arabia + Near East:
Bedouins
Saudis
Yemeni
Yemeni Jews
Palestinians
Druze
Lebanese
Jordanians
Syrians
Iraqi Jews
Iranians
Iranian Jews
Anatolia + Caucasus:
Cypriots
Turks
Armenians
Balkars
Chechens
Lezgins
Abkhasians
Adygei
North Ossetians
Georgians
Georgian Jews
Azerbaijani Jews
Kumyks
Nogais
Central Asia:
Turkmens
Uzbeks
Uzbekistani Jews
Tajiks
South Asia:
Kalash
Balochi
Brahui
Burusho
Sindhi
Pathan
Makrani
PJL
GIH+Gujaratis
Meghawal
Meena
Nihali
Dusadh
Gond
Brahmins-Central
Kshatriya
Dharkars
Low Caste - Central
Muslim
Chamar
Kol
Kanjars
Tharus
Kharia
Bhunjia
ITU
Brahmins-South
Low Caste-South
Sakilli
Cochin Jews
Bene Israel
Velmas
North Kannadi
Piramalai Kallars
Halakipikki
Kurumba
Malayan
Paniya
Chenchus
Lambadi
STU
BEB+Bengali
Ho
Bonda
Khasi
Savara
Juang
Asur
Gadaba
Santhal
Mawasi
Dhurwa
South Asia
Western South Asia
Central Asia
Yamnaya
Arabia + Near East
Anatolia + Caucasus
PC2 (9.1 %)
PC1 (59.2 %)
0,09
0,07
0,05
0,03
0,01
-0,01
-0,03
-0,05
-0,07
-0,05
-0,03
-0,01
0,01
0,03
0,05

Fig. S8. PCA analysis (for PC1 and PC2) including the Yamnaya. Information on the populations included in Table S3.

Table S1. List of complete mtDNA sequences belonging to South Asian autochthonous haplogroups.

Population codes: BEB – Bengali from Bangladesh; GIH – Gujarati Indian from Houston, Texas; ITU – Indian Telugu from the UK; PJL – Punjabi from Lahore, Pakistan; STU – Sri Lankan Tamil from the UK.

Sample	Haplogroup	Origin (Population)	Region	Reference
FJ770945	M	India	South Asia	(Fornarino et al. 2009)
JF742212	M	Nepal: Kathmandu	South Asia - Central	(Wang et al. 2012)
FJ383814	M	Orissa	South Asia - East	(Chandrasekar et al. 2009)
JX289110	M	Myanmar	Southeast Asia	(Summerer et al. 2014)
AY922300	M	Uttar Pradesh	South Asia - Central	(Sun et al. 2006)
HG04210	M	STU	South Asia - South	(Abecasis et al. 2012)
HG04076	M	ITU	South Asia - South	(Abecasis et al. 2012)
JX289097	M	Myanmar	Southeast Asia	(Summerer et al. 2014)
JX289130	M	Myanmar	Southeast Asia	(Summerer et al. 2014)
HG03594	M	BEB	South Asia - East	(Abecasis et al. 2012)
JX289131	M	Myanmar	Southeast Asia	(Summerer et al. 2014)
HG03611	M	BEB	South Asia - East	(Abecasis et al. 2012)
HG04033	M	STU	South Asia - South	(Abecasis et al. 2012)
HG03643	M	STU	South Asia - South	(Abecasis et al. 2012)
HG03668	M	PJL	South Asia - West	(Abecasis et al. 2012)
DQ246828	M	Bihar	South Asia - East	(Rajkumar et al. 2005)
FJ770941	M	Andhra Pradesh	South Asia - South	(Fornarino et al. 2009)
JX289123	M	Myanmar	Southeast Asia	(Summerer et al. 2014)
JX289119	M13'46'61	Myanmar	Southeast Asia	(Summerer et al. 2014)
FJ383772	M13'46'61	Nepal	South Asia - Central	(Chandrasekar et al. 2009)
FJ383770	M13'46'61	Nepal	South Asia - Central	(Chandrasekar et al. 2009)
FJ383773	M13'46'61	Nepal	South Asia - Central	(Chandrasekar et al. 2009)
FJ383771	M13'46'61	Nepal	South Asia - Central	(Chandrasekar et al. 2009)
HM030546	M13'46'61	China	East Asia	(Kong et al. 2011)
KK178	M13'46'61	Borneo: Kota Kinabalu	Southeast Asia	This study
VNM396	M13'46'61	Vietnam	Southeast Asia	This study
JX289113	M13'46'61	Myanmar	Southeast Asia	(Summerer et al. 2014)
FJ383769	M13'46'61	Arunachal Pradesh	South Asia - East	(Chandrasekar et al. 2009)
FJ383775	M13'46'61	Arunachal Pradesh	South Asia - East	(Chandrasekar et al. 2009)
FJ383776	M13'46'61	Arunachal Pradesh	South Asia - East	(Chandrasekar et al. 2009)
FJ383777	M13'46'61	Arunachal Pradesh	South Asia - East	(Chandrasekar et al. 2009)
FJ383774	M13'46'61	Arunachal Pradesh	South Asia - East	(Chandrasekar et al. 2009)
FJ770957	M13'46'61	Nepal	South Asia - Central	(Fornarino et al. 2009)
AY963577	M13'46'61	Semang (Jahai)	Southeast Asia	(Macaulay et al. 2005)
HG03743	M2	STU	South Asia - South	(Abecasis et al. 2012)
EU443499	M2	Malpaharia	South Asia - East	(Kumar et al. 2008)
FJ383284	M2	Jharkhand	South Asia - East	(Chandrasekar et al. 2009)
AY922303	M2	Andhra Pradesh	South Asia - South	(Sun et al. 2006)
AY922306	M2	Andhra Pradesh	South Asia - South	(Sun et al. 2006)
FJ383292	M2	Maharashtra	South Asia - South	(Chandrasekar et al. 2009)
HG03857	M2	STU	South Asia - South	(Abecasis et al. 2012)

Table S1. (*continued*)

Sample	Haplogroup	Origin (Population)	Region	Reference
HG03978	M2	ITU	South Asia - South	(Abecasis et al. 2012)
HG04042	M2	STU	South Asia - South	(Abecasis et al. 2012)
HG03866	M2	ITU	South Asia - South	(Abecasis et al. 2012)
HG03951	M2	STU	South Asia - South	(Abecasis et al. 2012)
FJ383254	M2	Rajasthan	South Asia - West	(Chandrasekar et al. 2009)
FJ383253	M2	Rajasthan	South Asia - West	(Chandrasekar et al. 2009)
FJ383251	M2	Rajasthan	South Asia - West	(Chandrasekar et al. 2009)
FJ383252	M2	Rajasthan	South Asia - West	(Chandrasekar et al. 2009)
EU443454	M2	Dungri Bhil	South Asia - West	(Kumar et al. 2008)
EU443457	M2	Dungri Bhil	South Asia - West	(Kumar et al. 2008)
EU443455	M2	Dungri Bhil	South Asia - West	(Kumar et al. 2008)
EU443456	M2	Dungri Bhil	South Asia - West	(Kumar et al. 2008)
FJ383246	M2	Maharashtra	South Asia - South	(Chandrasekar et al. 2009)
EU443443	M2	Maharashtra	South Asia - South	(Kumar et al. 2008)
EU443444	M2	Maharashtra	South Asia - South	(Kumar et al. 2008)
EU443445	M2	Maharashtra	South Asia - South	(Kumar et al. 2008)
GU480007	M2	Madhya Pradesh	South Asia - Central	(Sharma et al. 2012)
GU480005	M2	Madhya Pradesh	South Asia - Central	(Sharma et al. 2012)
EU443506	M2	Jharkhand	South Asia - East	(Kumar et al. 2008)
HG03585	M2	BEB	South Asia - East	(Abecasis et al. 2012)
EU443502	M2	Maharashtra	South Asia - South	(Kumar et al. 2008)
EU443490	M2	Gujarat	South Asia - West	(Kumar et al. 2008)
EU443489	M2	Gujarat	South Asia - West	(Kumar et al. 2008)
EU443468	M2	Maharashtra	South Asia - South	(Kumar et al. 2008)
FJ383272	M2	Maharashtra	South Asia - South	(Chandrasekar et al. 2009)
FJ383274	M2	Maharashtra	South Asia - South	(Chandrasekar et al. 2009)
EU443471	M2	Maharashtra	South Asia - South	(Kumar et al. 2008)
EU443500	M2	Maharashtra	South Asia - South	(Kumar et al. 2008)
EU443501	M2	Maharashtra	South Asia - South	(Kumar et al. 2008)
EU443470	M2	Maharashtra	South Asia - South	(Kumar et al. 2008)
EU443467	M2	Maharashtra	South Asia - South	(Kumar et al. 2008)
EU443469	M2	Maharashtra	South Asia - South	(Kumar et al. 2008)
FJ383273	M2	Maharashtra	South Asia - South	(Chandrasekar et al. 2009)
EU443503	M2	Maharashtra	South Asia - South	(Kumar et al. 2008)
EU443492	M2	Chhattisgarh	South Asia - Central	(Kumar et al. 2008)
EU443493	M2	Chhattisgarh	South Asia - Central	(Kumar et al. 2008)
EU443495	M2	Chhattisgarh	South Asia - Central	(Kumar et al. 2008)
EU443494	M2	Chhattisgarh	South Asia - Central	(Kumar et al. 2008)
NA21103	M2	GIH	South Asia - West	(Abecasis et al. 2012)
EU443478	M2	Chhattisgarh	South Asia - Central	(Kumar et al. 2008)
EU443477	M2	Chhattisgarh	South Asia - Central	(Kumar et al. 2008)
FJ383280	M2	Chhattisgarh	South Asia - Central	(Chandrasekar et al. 2009)

Table S1. (*continued*)

Sample	Haplogroup	Origin (Population)	Region	Reference
EU443479	M2	Chhattisgarh	South Asia - Central	(Kumar et al. 2008)
FJ383276	M2	Chhattisgarh	South Asia - Central	(Chandrasekar et al. 2009)
FJ383277	M2	Chhattisgarh	South Asia - Central	(Chandrasekar et al. 2009)
FJ383281	M2	Chhattisgarh	South Asia - Central	(Chandrasekar et al. 2009)
FJ383282	M2	Chhattisgarh	South Asia - Central	(Chandrasekar et al. 2009)
FJ383279	M2	Chhattisgarh	South Asia - Central	(Chandrasekar et al. 2009)
FJ383275	M2	Chhattisgarh	South Asia - Central	(Chandrasekar et al. 2009)
FJ383278	M2	Chhattisgarh	South Asia - Central	(Chandrasekar et al. 2009)
FJ383283	M2	Chhattisgarh	South Asia - Central	(Chandrasekar et al. 2009)
EU443476	M2	Chhattisgarh	South Asia - Central	(Kumar et al. 2008)
EU443480	M2	Chhattisgarh	South Asia - Central	(Kumar et al. 2008)
FJ383287	M2	Madhya Pradesh	South Asia - Central	(Chandrasekar et al. 2009)
FJ383288	M2	Madhya Pradesh	South Asia - Central	(Chandrasekar et al. 2009)
HG04211	M2	ITU	South Asia - South	(Abecasis et al. 2012)
EU597516	M2	Pakistan: Sindhi	South Asia - West	(Hartmann et al. 2009)
HG02691	M2	PJL	South Asia - West	(Abecasis et al. 2012)
HG03913	M2	BEB	South Asia - East	(Abecasis et al. 2012)
HG02654	M2	PJL	South Asia - West	(Abecasis et al. 2012)
HG02655	M2	PJL	South Asia - West	(Abecasis et al. 2012)
FJ383247	M2	Maharashtra	South Asia - South	(Chandrasekar et al. 2009)
EU443460	M2	Andhra Pradesh	South Asia - South	(Kumar et al. 2008)
FJ383244	M2	Maharashtra	South Asia - South	(Chandrasekar et al. 2009)
EU443472	M2	Maharashtra	South Asia - South	(Kumar et al. 2008)
EU443488	M2	Gujarat	South Asia - West	(Kumar et al. 2008)
EU443508	M2	Madhya Pradesh	South Asia - Central	(Kumar et al. 2008)
FJ383240	M2	Maharashtra	South Asia - South	(Chandrasekar et al. 2009)
EU443473	M2	Maharashtra	South Asia - South	(Kumar et al. 2008)
FJ383243	M2	Maharashtra	South Asia - South	(Chandrasekar et al. 2009)
EU443507	M2	Madhya Pradesh	South Asia - Central	(Kumar et al. 2008)
FJ383286	M2	Madhya Pradesh	South Asia - Central	(Chandrasekar et al. 2009)
EU443509	M2	Madhya Pradesh	South Asia - Central	(Kumar et al. 2008)
EU443510	M2	Madhya Pradesh	South Asia - Central	(Kumar et al. 2008)
FJ383290	M2	Madhya Pradesh	South Asia - Central	(Chandrasekar et al. 2009)
FJ383285	M2	Madhya Pradesh	South Asia - Central	(Chandrasekar et al. 2009)
FJ383289	M2	Madhya Pradesh	South Asia - Central	(Chandrasekar et al. 2009)
EU443505	M2	Jharkhand	South Asia - East	(Kumar et al. 2008)
FJ383291	M2	Bihar	South Asia - East	(Chandrasekar et al. 2009)
EU443450	M2	Karnataka	South Asia - South	(Kumar et al. 2008)
FJ383255	M2	Karnataka	South Asia - South	(Chandrasekar et al. 2009)
EU443464	M2	Karnataka	South Asia - South	(Kumar et al. 2008)
EU443462	M2	Karnataka	South Asia - South	(Kumar et al. 2008)
EU443513	M2	Orissa	South Asia - East	(Kumar et al. 2008)

Table S1. (*continued*)

Sample	Haplogroup	Origin (Population)	Region	Reference
EU443511	M2	Orissa	South Asia - East	(Kumar et al. 2008)
EU443514	M2	Orissa	South Asia - East	(Kumar et al. 2008)
EU443446	M2	Maharashtra	South Asia - South	(Kumar et al. 2008)
EU443447	M2	Maharashtra	South Asia - South	(Kumar et al. 2008)
EU443448	M2	Maharashtra	South Asia - South	(Kumar et al. 2008)
EU443491	M2	Gujarat	South Asia - West	(Kumar et al. 2008)
EU443487	M2	Gujarat	South Asia - West	(Kumar et al. 2008)
FJ383239	M2	Maharashtra	South Asia - South	(Chandrasekar et al. 2009)
FJ383242	M2	Maharashtra	South Asia - South	(Chandrasekar et al. 2009)
EU443475	M2	Maharashtra	South Asia - South	(Kumar et al. 2008)
FJ383238	M2	Maharashtra	South Asia - South	(Chandrasekar et al. 2009)
EU443474	M2	Maharashtra	South Asia - South	(Kumar et al. 2008)
FJ383245	M2	Maharashtra	South Asia - South	(Chandrasekar et al. 2009)
FJ383241	M2	Maharashtra	South Asia - South	(Chandrasekar et al. 2009)
EU443486	M2	Chhattisgarh	South Asia - Central	(Kumar et al. 2008)
EU443484	M2	Chhattisgarh	South Asia - Central	(Kumar et al. 2008)
EU443512	M2	Orissa	South Asia - East	(Kumar et al. 2008)
AY922305	M2	Andhra Pradesh	South Asia - South	(Sun et al. 2006)
NA21104	M2	GIH	South Asia - West	(Abecasis et al. 2012)
EU443452	M2	Karnataka	South Asia - South	(Kumar et al. 2008)
EU443453	M2	Karnataka	South Asia - South	(Kumar et al. 2008)
EU443451	M2	Karnataka	South Asia - South	(Kumar et al. 2008)
EU443449	M2	Betta Kuruba	South Asia - South	(Kumar et al. 2008)
FJ383265	M2	Karnataka	South Asia - South	(Chandrasekar et al. 2009)
FJ383258	M2	Karnataka	South Asia - South	(Chandrasekar et al. 2009)
FJ383263	M2	Karnataka	South Asia - South	(Chandrasekar et al. 2009)
FJ383262	M2	Karnataka	South Asia - South	(Chandrasekar et al. 2009)
FJ383261	M2	Karnataka	South Asia - South	(Chandrasekar et al. 2009)
FJ383260	M2	Karnataka	South Asia - South	(Chandrasekar et al. 2009)
FJ383259	M2	Karnataka	South Asia - South	(Chandrasekar et al. 2009)
FJ383257	M2	Karnataka	South Asia - South	(Chandrasekar et al. 2009)
FJ383266	M2	Karnataka	South Asia - South	(Chandrasekar et al. 2009)
FJ383269	M2	Karnataka	South Asia - South	(Chandrasekar et al. 2009)
FJ383270	M2	Karnataka	South Asia - South	(Chandrasekar et al. 2009)
FJ383264	M2	Karnataka	South Asia - South	(Chandrasekar et al. 2009)
FJ383256	M2	Karnataka	South Asia - South	(Chandrasekar et al. 2009)
FJ383268	M2	Karnataka	South Asia - South	(Chandrasekar et al. 2009)
FJ383271	M2	Karnataka	South Asia - South	(Chandrasekar et al. 2009)
FJ383267	M2	Karnataka	South Asia - South	(Chandrasekar et al. 2009)
HG03684	M2	STU	South Asia - South	(Abecasis et al. 2012)
EU443466	M2	Karnataka	South Asia - South	(Kumar et al. 2008)
EU443463	M2	Karnataka	South Asia - South	(Kumar et al. 2008)

Table S1. (*continued*)

Sample	Haplogroup	Origin (Population)	Region	Reference
EU443465	M2	Karnataka	South Asia - South	(Kumar et al. 2008)
HG04173	M2	BEB	South Asia - East	(Abecasis et al. 2012)
EU443458	M2	Andhra Pradesh	South Asia - South	Rao (direct submission)
EU443459	M2	Andhra Pradesh	South Asia - South	(Kumar et al. 2008)
EU443461	M2	Andhra Pradesh	South Asia - South	(Kumar et al. 2008)
AY922308	M2	Andhra Pradesh	South Asia - South	Sun (direct submission)
EU443485	M2	Chhattisgarh	South Asia - Central	(Kumar et al. 2008)
EU443481	M2	Chhattisgarh	South Asia - Central	(Kumar et al. 2008)
FJ383249	M2	Madhya Pradesh	South Asia - Central	(Chandrasekar et al. 2009)
FJ383250	M2	Madhya Pradesh	South Asia - Central	(Chandrasekar et al. 2009)
HG03007	M2	BEB	South Asia - East	(Abecasis et al. 2012)
HG03950	M2	STU	South Asia - South	(Abecasis et al. 2012)
FJ383248	M2	Maharashtra	South Asia - South	(Chandrasekar et al. 2009)
EU443483	M2	Chhattisgarh	South Asia - Central	(Kumar et al. 2008)
EU443482	M2	Chhattisgarh	South Asia - Central	(Kumar et al. 2008)
EU443504	M2	Jharkhand	South Asia - East	(Kumar et al. 2008)
NA20905	M2	GIH	South Asia - West	(Abecasis et al. 2012)
HM156675	M2	Andhra Pradesh	South Asia - South	(Govindaraj et al. 2011)
HM156678	M2	Andhra Pradesh	South Asia - South	(Govindaraj et al. 2011)
EU443496	M2	Jharkhand	South Asia - East	(Kumar et al. 2008)
EU443497	M2	Jharkhand	South Asia - East	(Kumar et al. 2008)
EU443498	M2	Jharkhand	South Asia - East	(Kumar et al. 2008)
NA20885	M2	GIH	South Asia - West	(Abecasis et al. 2012)
NA20882	M2	GIH	South Asia - West	(Abecasis et al. 2012)
NA20900	M2	GIH	South Asia - West	(Abecasis et al. 2012)
KC911426	M2	Iran: Persian	Southwest Asia	(Derenko et al. 2013)
DQ408676	M3	Kerala	South Asia - South	(Thangaraj et al. 2006)
KC887464	M3	Cambodia	Southeast Asia	(Zhang et al. 2013)
KC887465	M3	Cambodia	Southeast Asia	(Zhang et al. 2013)
KC887463	M3	Cambodia	Southeast Asia	(Zhang et al. 2013)
KC505095	M3	Cambodia	Southeast Asia	(Zhang et al. 2013)
KC887466	M3	Cambodia	Southeast Asia	(Zhang et al. 2013)
KC887467	M3	Cambodia	Southeast Asia	(Zhang et al. 2013)
KC887468	M3	Cambodia	Southeast Asia	(Zhang et al. 2013)
KC887469	M3	Cambodia	Southeast Asia	(Zhang et al. 2013)
KC887471	M3	Cambodia	Southeast Asia	(Zhang et al. 2013)
KC887474	M3	Cambodia	Southeast Asia	(Zhang et al. 2013)
KC887470	M3	Cambodia	Southeast Asia	(Zhang et al. 2013)
KF056281	M3	Sherpa	South Asia - Central	(Kang et al. 2013)
KF056282	M3	Sherpa	South Asia - Central	(Kang et al. 2013)
KF056283	M3	Sherpa	South Asia - Central	(Kang et al. 2013)
HG03781	M3	ITU	South Asia - South	(Abecasis et al. 2012)

Table S1. (*continued*)

Sample	Haplogroup	Origin (Population)	Region	Reference
FJ770946	M3	India	South Asia	(Fornarino et al. 2009)
JF742206	M3	Nepal: Kathmandu	South Asia - Central	(Wang et al. 2012)
DQ246826	M3	Maharashtra	South Asia - South	(Rajkumar et al. 2005)
F6	M3	Myanmar	Southeast Asia	This study
HG03910	M3	BEB	South Asia - East	(Abecasis et al. 2012)
HG04161	M3	BEB	South Asia - East	(Abecasis et al. 2012)
HG04019	M3	ITU	South Asia - South	(Abecasis et al. 2012)
HG03974	M3	ITU	South Asia - South	(Abecasis et al. 2012)
HG03976	M3	ITU	South Asia - South	(Abecasis et al. 2012)
FJ383522	M3	Chhattisgarh	South Asia - Central	(Chandrasekar et al. 2009)
FJ383513	M3	Chhattisgarh	South Asia - Central	(Chandrasekar et al. 2009)
FJ383523	M3	Chhattisgarh	South Asia - Central	(Chandrasekar et al. 2009)
FJ383524	M3	Chhattisgarh	South Asia - Central	(Chandrasekar et al. 2009)
FJ383525	M3	Chhattisgarh	South Asia - Central	(Chandrasekar et al. 2009)
FJ383526	M3	Chhattisgarh	South Asia - Central	(Chandrasekar et al. 2009)
FJ383527	M3	Rajasthan	South Asia - West	(Chandrasekar et al. 2009)
KJ446720	M3	Pakistan: Sindhi	South Asia - West	HGDP - Zheng et al. (unpublished)
HG03616	M3	BEB	South Asia - East	(Abecasis et al. 2012)
KJ446721	M3	Pakistan: Sindhi	South Asia - West	HGDP - Zheng et al. (unpublished)
HG04155	M3	BEB	South Asia - East	(Abecasis et al. 2012)
AY922263	M3	Uttar Pradesh	South Asia - Central	(Sun et al. 2006)
HM852820	M3	Iran	Southwest Asia	(Schönberg et al. 2011)
JF742216	M3	Nepal: Kathmandu	South Asia - Central	(Wang et al. 2012)
HG03940	M3	BEB	South Asia - East	(Abecasis et al. 2012)
HG03824	M3	BEB	South Asia - East	(Abecasis et al. 2012)
HG04183	M3	BEB	South Asia - East	(Abecasis et al. 2012)
HG04186	M3	BEB	South Asia - East	(Abecasis et al. 2012)
FJ383538	M3	Gujarat	South Asia - West	(Chandrasekar et al. 2009)
FJ383536	M3	Gujarat	South Asia - West	(Chandrasekar et al. 2009)
FJ383539	M3	Gujarat	South Asia - West	(Chandrasekar et al. 2009)
FJ383537	M3	Gujarat	South Asia - West	(Chandrasekar et al. 2009)
FJ383540	M3	Gujarat	South Asia - West	(Chandrasekar et al. 2009)
HG02793	M3	PJL	South Asia - West	(Abecasis et al. 2012)
KJ446716	M3	Pakistan: Makrani	South Asia - West	HGDP - Zheng et al. (unpublished)
KJ446717	M3	Pakistan: Makrani	South Asia - West	HGDP - Zheng et al. (unpublished)
KJ446713	M3	Pakistan: Hazara	South Asia - West	HGDP - Zheng et al. (unpublished)
KJ446714	M3	Pakistan: Hazara	South Asia - West	HGDP - Zheng et al. (unpublished)
KC911530	M3	Iran: Persian	Southwest Asia	(Derenko et al. 2013)

Table S1. (*continued*)

Sample	Haplogroup	Origin (Population)	Region	Reference
KJ446719	M3	Pakistan: Brahui	South Asia - West	HGDP - Zheng et al. (unpublished)
KJ446718	M3	Pakistan: Brahui	South Asia - West	HGDP - Zheng et al. (unpublished)
HG03686	M3	STU	South Asia - South	(Abecasis et al. 2012)
JQ702447	M3	unknown	unknown	(Behar et al. 2012)
JQ704107	M3	unknown	unknown	(Behar et al. 2012)
HG03925	M3	BEB	South Asia - East	(Abecasis et al. 2012)
HG03907	M3	BEB	South Asia - East	(Abecasis et al. 2012)
JF742210	M3	Nepal: Kathmandu	South Asia - Central	(Wang et al. 2012)
HM156695	M3	Andhra Pradesh: Mahabubnagar	South Asia - South	(Govindaraj et al. 2011)
FJ383514	M3	Chhattisgarh	South Asia - Central	(Chandrasekar et al. 2009)
FJ383515	M3	Chhattisgarh	South Asia - Central	(Chandrasekar et al. 2009)
FJ383516	M3	Chhattisgarh	South Asia - Central	(Chandrasekar et al. 2009)
FJ383517	M3	Chhattisgarh	South Asia - Central	(Chandrasekar et al. 2009)
FJ383520	M3	Chhattisgarh	South Asia - Central	(Chandrasekar et al. 2009)
FJ383521	M3	Chhattisgarh	South Asia - Central	(Chandrasekar et al. 2009)
FJ383535	M3	Chhattisgarh	South Asia - Central	(Chandrasekar et al. 2009)
FJ383519	M3	Chhattisgarh	South Asia - Central	(Chandrasekar et al. 2009)
FJ383534	M3	Chhattisgarh	South Asia - Central	(Chandrasekar et al. 2009)
FJ383518	M3	Chhattisgarh	South Asia - Central	(Chandrasekar et al. 2009)
AY922266	M3	Andhra Pradesh	South Asia - South	(Sun et al. 2006)
KJ446722	M3	Pakistan: Burusho	South Asia - West	HGDP - Zheng et al. (unpublished)
HG03229	M3	PJL	South Asia - West	(Abecasis et al. 2012)
HG03636	M3	PJL	South Asia - West	(Abecasis et al. 2012)
NA21092	M3	GIH	South Asia - West	(Abecasis et al. 2012)
HG01583	M3	PJL	South Asia - West	(Abecasis et al. 2012)
HG02684	M3	PJL	South Asia - West	(Abecasis et al. 2012)
KJ446723	M3	Pakistan: Pathan	South Asia - West	HGDP - Zheng et al. (unpublished)
KJ446724	M3	Pakistan: Sindhi	South Asia - West	HGDP - Zheng et al. (unpublished)
KJ446725	M3	Pakistan: Balochi	South Asia - West	HGDP - Zheng et al. (unpublished)
KJ446726	M3	Pakistan: Balochi	South Asia - West	HGDP - Zheng et al. (unpublished)
HG03717	M3	ITU	South Asia - South	(Abecasis et al. 2012)
HG03772	M3	ITU	South Asia - South	(Abecasis et al. 2012)
HG03773	M3	ITU	South Asia - South	(Abecasis et al. 2012)
HG04017	M3	ITU	South Asia - South	(Abecasis et al. 2012)
HG04054	M3	ITU	South Asia - South	(Abecasis et al. 2012)
HG04096	M3	ITU	South Asia - South	(Abecasis et al. 2012)
FJ383531	M3	Karnataka	South Asia - South	(Chandrasekar et al. 2009)
FJ383533	M3	Karnataka	South Asia - South	(Chandrasekar et al. 2009)

Table S1. (*continued*)

Sample	Haplogroup	Origin (Population)	Region	Reference
FJ383530	M3	Karnataka	South Asia - South	(Chandrasekar et al. 2009)
FJ383528	M3	Karnataka	South Asia - South	(Chandrasekar et al. 2009)
FJ383532	M3	Karnataka	South Asia - South	(Chandrasekar et al. 2009)
HG03968	M3	ITU	South Asia - South	(Abecasis et al. 2012)
FJ383529	M3	Karnataka	South Asia - South	(Chandrasekar et al. 2009)
GU810074	M3	Thailand	Southeast Asia	Pradutkanchana et al. (unpublished)
HG03875	M3	ITU	South Asia - South	(Abecasis et al. 2012)
FJ383541	M3	Maharashtra	South Asia - South	(Chandrasekar et al. 2009)
KJ446715	M3	Pakistan: Brahui	South Asia - West	HGDP - Zheng et al. (unpublished)
HG04164	M3	BEB	South Asia - East	(Abecasis et al. 2012)
FJ383542	M3	Chhattisgarh	South Asia - Central	(Chandrasekar et al. 2009)
HG03745	M3	STU	South Asia - South	(Abecasis et al. 2012)
FJ770942	M3	Andhra Pradesh	South Asia - South	(Fornarino et al. 2009)
HG04140	M3	BEB	South Asia - East	(Abecasis et al. 2012)
HG03012	M3	BEB	South Asia - East	(Abecasis et al. 2012)
FJ770965	M3	Nepal	South Asia - Central	(Fornarino et al. 2009)
FJ383459	M3	Maharashtra	South Asia - South	(Chandrasekar et al. 2009)
FJ383472	M3	Karnataka	South Asia - South	(Chandrasekar et al. 2009)
FJ383474	M3	Karnataka	South Asia - South	(Chandrasekar et al. 2009)
FJ383471	M3	Karnataka	South Asia - South	(Chandrasekar et al. 2009)
FJ383462	M3	Karnataka	South Asia - South	(Chandrasekar et al. 2009)
FJ383476	M3	Karnataka	South Asia - South	(Chandrasekar et al. 2009)
FJ383464	M3	Karnataka	South Asia - South	(Chandrasekar et al. 2009)
FJ383473	M3	Karnataka	South Asia - South	(Chandrasekar et al. 2009)
FJ383475	M3	Karnataka	South Asia - South	(Chandrasekar et al. 2009)
FJ383467	M3	Karnataka	South Asia - South	(Chandrasekar et al. 2009)
FJ383468	M3	Karnataka	South Asia - South	(Chandrasekar et al. 2009)
FJ383469	M3	Karnataka	South Asia - South	(Chandrasekar et al. 2009)
FJ383477	M3	Karnataka	South Asia - South	(Chandrasekar et al. 2009)
FJ383478	M3	Karnataka	South Asia - South	(Chandrasekar et al. 2009)
FJ383479	M3	Karnataka	South Asia - South	(Chandrasekar et al. 2009)
FJ383480	M3	Karnataka	South Asia - South	(Chandrasekar et al. 2009)
FJ383481	M3	Karnataka	South Asia - South	(Chandrasekar et al. 2009)
FJ383466	M3	Karnataka	South Asia - South	(Chandrasekar et al. 2009)
FJ383483	M3	Karnataka	South Asia - South	(Chandrasekar et al. 2009)
FJ383484	M3	Karnataka	South Asia - South	(Chandrasekar et al. 2009)
FJ383485	M3	Karnataka	South Asia - South	(Chandrasekar et al. 2009)
FJ383461	M3	Karnataka	South Asia - South	(Chandrasekar et al. 2009)
FJ383465	M3	Karnataka	South Asia - South	(Chandrasekar et al. 2009)
FJ383470	M3	Karnataka	South Asia - South	(Chandrasekar et al. 2009)
FJ383460	M3	Karnataka	South Asia - South	(Chandrasekar et al. 2009)

Table S1. (*continued*)

Sample	Haplogroup	Origin (Population)	Region	Reference
FJ383463	M3	Karnataka	South Asia - South	(Chandrasekar et al. 2009)
FJ383482	M3	Karnataka	South Asia - South	(Chandrasekar et al. 2009)
HG03948	M4’67	STU	South Asia - South	(Abecasis et al. 2012)
HG03673	M4’67	STU	South Asia - South	(Abecasis et al. 2012)
NA20894	M4’67	GIH	South Asia - West	(Abecasis et al. 2012)
HG02688	M4’67	PJL	South Asia - West	(Abecasis et al. 2012)
FJ383293	M4’67	Maharashtra	South Asia - South	(Chandrasekar et al. 2009)
FJ383296	M4’67	Maharashtra	South Asia - South	(Chandrasekar et al. 2009)
HG02658	M4’67	PJL	South Asia - West	(Abecasis et al. 2012)
HG03687	M4’67	STU	South Asia - South	(Abecasis et al. 2012)
KJ446709	M4’67	Pakistan: Makrani	South Asia - West	HGDP - Zheng et al. (unpublished)
KJ446710	M4’67	Pakistan: Makrani	South Asia - West	HGDP - Zheng et al. (unpublished)
KJ446711	M4’67	Pakistan: Hazara	South Asia - West	HGDP - Zheng et al. (unpublished)
KJ446712	M4’67	Pakistan: Hazara	South Asia - West	HGDP - Zheng et al. (unpublished)
KC911394	M4’67	Iran: Persian	Southwest Asia	(Derenko et al. 2013)
HG03720	M4’67	ITU	South Asia - South	(Abecasis et al. 2012)
HG03792	M4’67	ITU	South Asia - South	(Abecasis et al. 2012)
NA20853	M4’67	GIH	South Asia - West	(Abecasis et al. 2012)
HG03746	M4’67	STU	South Asia - South	(Abecasis et al. 2012)
AY922278	M4’67	Andhra Pradesh	South Asia - South	(Sun et al. 2006)
KC911313	M4’67	Iran: Qashqai	Southwest Asia	(Derenko et al. 2013)
DQ408679	M4’67	Gujarat	South Asia - West	(Thangaraj et al. 2006)
KJ446708	M4’67	Pakistan: Pathan	South Asia - West	HGDP - Zheng et al. (unpublished)
HG03805	M4’67	BEB	South Asia - East	(Abecasis et al. 2012)
AY922291	M4’67	Andhra Pradesh	South Asia - South	(Sun et al. 2006)
AY922297	M4’67	Andhra Pradesh	South Asia - South	(Sun et al. 2006)
HG03808	M4’67	BEB	South Asia - East	(Abecasis et al. 2012)
HG04176	M4’67	BEB	South Asia - East	(Abecasis et al. 2012)
HG04229	M4’67	STU	South Asia - South	(Abecasis et al. 2012)
HG02792	M4’67	PJL	South Asia - West	(Abecasis et al. 2012)
HM036536	M4’67	Great Himalayas: Ladakh tribe	South Asia - Central	Sharma et al. (unpublished)
KJ446732	M4’67	Pakistan: Sindhi	South Asia - West	HGDP - Zheng et al. (unpublished)
HM036576	M4’67	Great Himalayas: Ladakh tribe	South Asia - Central	Sharma et al. (unpublished)
KJ446731	M4’67	Pakistan: Balochi	South Asia - West	HGDP - Zheng et al. (unpublished)
GQ337623	M4’67	India	South Asia	(Palanichamy et al. 2014)
JX289111	M4’67	Myanmar	Southeast Asia	(Summerer et al. 2014)
GQ337562	M4’67	India	South Asia	(Palanichamy et al. 2014)

Table S1. (*continued*)

Sample	Haplogroup	Origin (Population)	Region	Reference
AY922261	M4’67	Uttar Pradesh	South Asia - Central	(Sun et al. 2006)
FJ383297	M4’67	Maharashtra	South Asia - South	(Chandrasekar et al. 2009)
FJ383298	M4’67	Maharashtra	South Asia - South	(Chandrasekar et al. 2009)
FJ383299	M4’67	Maharashtra	South Asia - South	(Chandrasekar et al. 2009)
FJ383295	M4’67	Maharashtra	South Asia - South	(Chandrasekar et al. 2009)
KJ446730	M4’67	Pakistan: Balochi	South Asia - West	HGDP - Zheng et al. (unpublished)
HG02699	M4’67	PJL	South Asia - West	(Abecasis et al. 2012)
HG04177	M4’67	BEB	South Asia - East	(Abecasis et al. 2012)
HG03718	M4’67	ITU	South Asia - South	(Abecasis et al. 2012)
HG04063	M4’67	ITU	South Asia - South	(Abecasis et al. 2012)
FJ383300	M4’67	Arunachal Pradesh	South Asia - East	(Chandrasekar et al. 2009)
FJ383294	M4’67	Arunachal Pradesh	South Asia - East	(Chandrasekar et al. 2009)
HG03672	M4’67	STU	South Asia - South	(Abecasis et al. 2012)
JX289108	M4’67	Myanmar	Southeast Asia	(Summerer et al. 2014)
FJ383337	M4’67	Bihar	South Asia - East	(Chandrasekar et al. 2009)
FJ383335	M4’67	Orissa	South Asia - East	(Chandrasekar et al. 2009)
JQ705662	M4’67	United Kingdom	Europe	(Behar et al. 2012)
KC911475	M4’67	Iran: Persian	Southwest Asia	(Derenko et al. 2013)
KJ446728	M4’67	Pakistan: Sindhi	South Asia - West	HGDP - Zheng et al. (unpublished)
HG03016	M4’67	PJL	South Asia - West	(Abecasis et al. 2012)
HG03663	M4’67	PJL	South Asia - West	(Abecasis et al. 2012)
HG03867	M4’67	ITU	South Asia - South	(Abecasis et al. 2012)
HG04080	M4’67	ITU	South Asia - South	(Abecasis et al. 2012)
HG04146	M4’67	BEB	South Asia - East	(Abecasis et al. 2012)
HG03765	M4’67	PJL	South Asia - West	(Abecasis et al. 2012)
HG04001	M4’67	ITU	South Asia - South	(Abecasis et al. 2012)
FJ383336	M4’67	Madhya Pradesh	South Asia - Central	(Chandrasekar et al. 2009)
DQ408674	M4’67	Bihar	South Asia - East	(Thangaraj et al. 2006)
DQ246825	M4’67	Bihar	South Asia - East	(Rajkumar et al. 2005)
JQ446407	M4’67	India	South Asia	Khan et al. (unpublished)
HM156679	M4’67	Andhra Pradesh: Mahabubnagar	South Asia - South	(Govindaraj et al. 2011)
JF742214	M4’67	Nepal	South Asia - Central	(Wang et al. 2012)
KJ446729	M4’67	China: Uygur	East Asia	HGDP - Zheng et al. (unpublished)
KF055880	M4’67	Spain: Romani	Europe	(Gómez-Carballa et al. 2013)
AY922270	M4’67	Uttar Pradesh	South Asia - Central	(Sun et al. 2006)
HG04060	M4’67	ITU	South Asia - South	(Abecasis et al. 2012)
HG03730	M4’67	ITU	South Asia - South	(Abecasis et al. 2012)
HG03646	M4’67	STU	South Asia - South	(Abecasis et al. 2012)
JX289116	M4’67	Myanmar	Southeast Asia	(Summerer et al. 2014)

Table S1. (*continued*)

Sample	Haplogroup	Origin (Population)	Region	Reference
KF056274	M4’67	Sherpa	South Asia - Central	(Kang et al. 2013)
FJ770947	M4’67	India	South Asia	(Fornarino et al. 2009)
FJ770963	M4’67	Nepal	South Asia - Central	(Fornarino et al. 2009)
FJ383395	M4’67	Maharashtra	South Asia - South	(Chandrasekar et al. 2009)
FJ383397	M4’67	Maharashtra	South Asia - South	(Chandrasekar et al. 2009)
FJ383399	M4’67	Maharashtra	South Asia - South	(Chandrasekar et al. 2009)
FJ383400	M4’67	Maharashtra	South Asia - South	(Chandrasekar et al. 2009)
FJ383401	M4’67	Maharashtra	South Asia - South	(Chandrasekar et al. 2009)
FJ383398	M4’67	Maharashtra	South Asia - South	(Chandrasekar et al. 2009)
FJ383396	M4’67	Maharashtra	South Asia - South	(Chandrasekar et al. 2009)
FJ383403	M4’67	Orissa	South Asia - East	(Chandrasekar et al. 2009)
FJ383405	M4’67	Bihar	South Asia - East	(Chandrasekar et al. 2009)
FJ383394	M4’67	Madhya Pradesh	South Asia - Central	(Chandrasekar et al. 2009)
FJ383404	M4’67	Bihar	South Asia - East	(Chandrasekar et al. 2009)
FJ383402	M4’67	Madhya Pradesh	South Asia - Central	(Chandrasekar et al. 2009)
HG03809	M4’67	BEB	South Asia - East	(Abecasis et al. 2012)
HG03488	M4’67	PJL	South Asia - West	(Abecasis et al. 2012)
HG03702	M4’67	PJL	South Asia - West	(Abecasis et al. 2012)
AY922290	M4’67	Andhra Pradesh	South Asia - South	(Sun et al. 2006)
AY922286	M4’67	Uttar Pradesh	South Asia - Central	(Sun et al. 2006)
NA21109	M4’67	GIH	South Asia - West	(Abecasis et al. 2012)
NA20849	M4’67	GIH	South Asia - West	(Abecasis et al. 2012)
NA21133	M4’67	GIH	South Asia - West	(Abecasis et al. 2012)
HG03894	M4’67	STU	South Asia - South	(Abecasis et al. 2012)
HG03693	M4’67	STU	South Asia - South	(Abecasis et al. 2012)
NA21100	M4’67	GIH	South Asia - West	(Abecasis et al. 2012)
HG03914	M4’67	BEB	South Asia - East	(Abecasis et al. 2012)
HG03782	M4’67	ITU	South Asia - South	(Abecasis et al. 2012)
KF161562	M4’67	Denmark	Europe	(Li et al. 2014)
HG02687	M4’67	PJL	South Asia - West	(Abecasis et al. 2012)
HG03660	M4’67	PJL	South Asia - West	(Abecasis et al. 2012)
FJ383659	M4’67	Gujarat	South Asia - West	(Chandrasekar et al. 2009)
FJ383658	M4’67	Gujarat	South Asia - West	(Chandrasekar et al. 2009)
HG02780	M4’67	PJL	South Asia - West	(Abecasis et al. 2012)
FJ383675	M4’67	Madhya Pradesh	South Asia - Central	(Chandrasekar et al. 2009)
FJ383673	M4’67	Madhya Pradesh	South Asia - Central	(Chandrasekar et al. 2009)
HG04090	M4’67	ITU	South Asia - South	(Abecasis et al. 2012)
FJ383657	M4’67	Karnataka	South Asia - South	(Chandrasekar et al. 2009)
FJ383667	M4’67	Maharashtra	South Asia - South	(Chandrasekar et al. 2009)
JF742213	M4’67	Nepal: Kathmandu	South Asia - Central	(Wang et al. 2012)
HG03237	M4’67	PJL	South Asia - West	(Abecasis et al. 2012)
FJ383671	M4’67	Madhya Pradesh	South Asia - Central	(Chandrasekar et al. 2009)

Table S1. (*continued*)

Sample	Haplogroup	Origin (Population)	Region	Reference
HM156694	M4’67	Andhra Pradesh	South Asia - South	(Govindaraj et al. 2011)
HG02690	M4’67	PJL	South Asia - West	(Abecasis et al. 2012)
KJ446736	M4’67	Palestinian	Southwest Asia	HGDP - Zheng et al. (unpublished)
KJ446738	M4’67	Palestinian	Southwest Asia	HGDP - Zheng et al. (unpublished)
KJ446739	M4’67	Palestinian	Southwest Asia	HGDP - Zheng et al. (unpublished)
KP076664	M4’67	France	Europe	Family Tree
EU597504	M4’67	Pakistan: Sindhi	South Asia - West	(Hartmann et al. 2009)
HG03871	M4’67	ITU	South Asia - South	(Abecasis et al. 2012)
FJ383672	M4’67	Maharashtra	South Asia - South	(Chandrasekar et al. 2009)
FJ383670	M4’67	Maharashtra	South Asia - South	(Chandrasekar et al. 2009)
FJ383676	M4’67	Maharashtra	South Asia - South	(Chandrasekar et al. 2009)
FJ383678	M4’67	Gujarat	South Asia - West	(Chandrasekar et al. 2009)
FJ383677	M4’67	Maharashtra	South Asia - South	(Chandrasekar et al. 2009)
FJ383679	M4’67	Gujarat	South Asia - West	(Chandrasekar et al. 2009)
HG03803	M4’67	BEB	South Asia - East	(Abecasis et al. 2012)
HG03941	M4’67	BEB	South Asia - East	(Abecasis et al. 2012)
HG04141	M4’67	BEB	South Asia - East	(Abecasis et al. 2012)
HG03919	M4’67	BEB	South Asia - East	(Abecasis et al. 2012)
KJ446735	M4’67	Pakistan: Sindhi	South Asia - West	HGDP - Zheng et al. (unpublished)
AY922258	M4’67	Uttar Pradesh	South Asia - Central	(Sun et al. 2006)
FJ748727	M4’67	China	East Asia	(Ji et al. 2012)
FJ383662	M4’67	Rajasthan	South Asia - West	(Chandrasekar et al. 2009)
FJ383663	M4’67	Rajasthan	South Asia - West	(Chandrasekar et al. 2009)
AY922254	M4’67	Andhra Pradesh	South Asia - South	(Sun et al. 2006)
AY289072	M4’67	Koraga	South Asia - South	(Ingman and Gyllensten 2003)
FJ383660	M4’67	Rajasthan	South Asia - West	(Chandrasekar et al. 2009)
FJ383666	M4’67	Rajasthan	South Asia - West	(Chandrasekar et al. 2009)
FJ383665	M4’67	Rajasthan	South Asia - West	(Chandrasekar et al. 2009)
FJ383661	M4’67	Rajasthan	South Asia - West	(Chandrasekar et al. 2009)
HG03846	M4’67	STU	South Asia - South	(Abecasis et al. 2012)
KJ446733	M4’67	Pakistan: Sindhi	South Asia - West	HGDP - Zheng et al. (unpublished)
HG03990	M4’67	STU	South Asia - South	(Abecasis et al. 2012)
FJ383674	M4’67	Madhya Pradesh	South Asia - Central	(Chandrasekar et al. 2009)
NA21101	M4’67	GIH	South Asia - West	(Abecasis et al. 2012)
GU480014	M4’67	Madhya Pradesh	South Asia - Central	(Sharma et al. 2012)
HG04100	M4’67	STU	South Asia - South	(Abecasis et al. 2012)
AF382013	M4’67	India	South Asia	(Maca-Meyer et al. 2001)
HG03598	M4’67	BEB	South Asia - East	(Abecasis et al. 2012)

Table S1. (*continued*)

Sample	Haplogroup	Origin (Population)	Region	Reference
HG04194	M4’67	BEB	South Asia - East	(Abecasis et al. 2012)
HM036550	M4’67	Great Himalayas: Ladakh tribe	South Asia - Central	Sharma et al. (unpublished)
HM036539	M4’67	Great Himalayas: Ladakh tribe	South Asia - Central	Sharma et al. (unpublished)
AY922257	M4’67	Andhra Pradesh	South Asia - South	(Sun et al. 2006)
AY922268	M4’67	Uttar Pradesh	South Asia - Central	(Sun et al. 2006)
NA21135	M4’67	GIH	South Asia - West	(Abecasis et al. 2012)
AY289071	M4’67	Kannada	South Asia - South	(Ingman and Gyllensten 2003)
FJ383664	M4’67	Rajasthan	South Asia - West	(Chandrasekar et al. 2009)
HG04188	M4’67	BEB	South Asia - East	(Abecasis et al. 2012)
AY922277	M4’67	Andhra Pradesh	South Asia - South	(Sun et al. 2006)
HG03715	M4’67	ITU	South Asia - South	(Abecasis et al. 2012)
JQ702987	M4’67	unknown	unknown	(Behar et al. 2012)
HG03848	M4’67	STU	South Asia - South	(Abecasis et al. 2012)
KM043056	M4’67	Punjab: Jatt	South Asia - West	Family Tree
FJ383669	M4’67	Maharashtra	South Asia - South	(Chandrasekar et al. 2009)
FJ383668	M4’67	Maharashtra	South Asia - South	(Chandrasekar et al. 2009)
HM156674	M4’67	Andhra Pradesh	South Asia - South	(Govindaraj et al. 2011)
AY922255	M4’67	Andhra Pradesh	South Asia - South	(Sun et al. 2006)
HG03600	M4’67	BEB	South Asia - East	(Abecasis et al. 2012)
KJ446734	M4’67	Pakistan: Makrani	South Asia - West	HGDP - Zheng et al. (unpublished)
HG03934	M4’67	BEB	South Asia - East	(Abecasis et al. 2012)
HG04144	M4’67	BEB	South Asia - East	(Abecasis et al. 2012)
AY922256	M4’67	Uttar Pradesh	South Asia - Central	(Sun et al. 2006)
HG02700	M4’67	PJL	South Asia - West	(Abecasis et al. 2012)
HG03690	M4’67	STU	South Asia - South	(Abecasis et al. 2012)
HG03830	M4’67	BEB	South Asia - East	(Abecasis et al. 2012)
FJ383656	M4’67	Nepal	South Asia - Central	(Chandrasekar et al. 2009)
FJ383654	M4’67	Nepal	South Asia - Central	(Chandrasekar et al. 2009)
FJ383655	M4’67	Nepal	South Asia - Central	(Chandrasekar et al. 2009)
GU480009	M4’67	Madhya Pradesh	South Asia - Central	(Sharma et al. 2012)
AY922267	M4’67	Uttar Pradesh	South Asia - Central	(Sun et al. 2006)
NA21120	M4’67	GIH	South Asia - West	(Abecasis et al. 2012)
FJ383693	M4’67	Orissa	South Asia - East	(Chandrasekar et al. 2009)
FJ383694	M4’67	Orissa	South Asia - East	(Chandrasekar et al. 2009)
FJ383684	M4’67	Rajasthan	South Asia - West	(Chandrasekar et al. 2009)
FJ383686	M4’67	Arunachal Pradesh	South Asia - East	(Chandrasekar et al. 2009)
DQ408678	M4’67	Gujarat	South Asia - West	(Thangaraj et al. 2006)
FJ383687	M4’67	Maharashtra	South Asia - South	(Chandrasekar et al. 2009)
FJ383688	M4’67	Maharashtra	South Asia - South	(Chandrasekar et al. 2009)

Table S1. (*continued*)

Sample	Haplogroup	Origin (Population)	Region	Reference
AY922265	M4'67	Andhra Pradesh	South Asia - South	(Sun et al. 2006)
FJ383689	M4'67	Gujarat	South Asia - West	(Chandrasekar et al. 2009)
FJ383692	M4'67	Madhya Pradesh	South Asia - Central	(Chandrasekar et al. 2009)
FJ383691	M4'67	Madhya Pradesh	South Asia - Central	(Chandrasekar et al. 2009)
FJ383690	M4'67	Madhya Pradesh	South Asia - Central	(Chandrasekar et al. 2009)
FJ383685	M4'67	Rajasthan	South Asia - West	(Chandrasekar et al. 2009)
FJ383695	M4'67	Rajasthan	South Asia - West	(Chandrasekar et al. 2009)
FJ383683	M4'67	Rajasthan	South Asia - West	(Chandrasekar et al. 2009)
JF742211	M4'67	Nepal: Kathmandu	South Asia - Central	(Wang et al. 2012)
JX289107	M4'67	Myanmar	Southeast Asia	(Summerer et al. 2014)
JF742207	M4'67	Nepal: Kathmandu	South Asia - Central	(Wang et al. 2012)
FJ770954	M4'67	Nepal	South Asia - Central	(Fornarino et al. 2009)
FJ770958	M4'67	Nepal	South Asia - Central	(Fornarino et al. 2009)
AY922301	M4'67	Uttar Pradesh	South Asia - Central	(Sun et al. 2006)
FJ383429	M4'67	Arunachal Pradesh	South Asia - East	(Chandrasekar et al. 2009)
FJ383428	M4'67	Arunachal Pradesh	South Asia - East	(Chandrasekar et al. 2009)
HG03631	M4'67	PJL	South Asia - West	(Abecasis et al. 2012)
FJ770948	M4'67	India	South Asia	(Fornarino et al. 2009)
FJ383437	M4'67	Bihar	South Asia - East	(Chandrasekar et al. 2009)
FJ383436	M4'67	Bihar	South Asia - East	(Chandrasekar et al. 2009)
HG03779	M4'67	ITU	South Asia - South	(Abecasis et al. 2012)
FJ383431	M4'67	Maharashtra	South Asia - South	(Chandrasekar et al. 2009)
FJ383430	M4'67	Maharashtra	South Asia - South	(Chandrasekar et al. 2009)
HG03905	M4'67	BEB	South Asia - East	(Abecasis et al. 2012)
FJ383435	M4'67	Bihar	South Asia - East	(Chandrasekar et al. 2009)
FJ383434	M4'67	Bihar	South Asia - East	(Chandrasekar et al. 2009)
FJ383433	M4'67	Madhya Pradesh	South Asia - Central	(Chandrasekar et al. 2009)
FJ383432	M4'67	Madhya Pradesh	South Asia - Central	(Chandrasekar et al. 2009)
HG03640	M4'67	PJL	South Asia - West	(Abecasis et al. 2012)
HG02728	M4'67	PJL	South Asia - West	(Abecasis et al. 2012)
FJ748709	M4'67	China	East Asia	(Ji et al. 2012)
FJ383813	M4'67	Madhya Pradesh	South Asia - Central	(Chandrasekar et al. 2009)
FJ383812	M4'67	Madhya Pradesh	South Asia - Central	(Chandrasekar et al. 2009)
NA21090	M4'67	GIH	South Asia - West	(Abecasis et al. 2012)
FJ383737	M4'67	Chhattisgarh	South Asia - Central	(Chandrasekar et al. 2009)
FJ383736	M4'67	Chhattisgarh	South Asia - Central	(Chandrasekar et al. 2009)
FJ383739	M4'67	Chhattisgarh	South Asia - Central	(Chandrasekar et al. 2009)
FJ383738	M4'67	Chhattisgarh	South Asia - Central	(Chandrasekar et al. 2009)
FJ383740	M4'67	Chhattisgarh	South Asia - Central	(Chandrasekar et al. 2009)
FJ383741	M4'67	Chhattisgarh	South Asia - Central	(Chandrasekar et al. 2009)
FJ770971	M4'67	Nepal	South Asia - Central	(Fornarino et al. 2009)
GU480012	M4'67	Madhya Pradesh	South Asia - Central	(Sharma et al. 2012)

Table S1. (*continued*)

Sample	Haplogroup	Origin (Population)	Region	Reference
AY922283	M4’67	Andhra Pradesh	South Asia - South	(Sun et al. 2006)
HG03607	M4’67	BEB	South Asia - East	(Abecasis et al. 2012)
NA20881	M4’67	GIH	South Asia - West	(Abecasis et al. 2012)
NA20891	M4’67	GIH	South Asia - West	(Abecasis et al. 2012)
HG03752	M4’67	STU	South Asia - South	(Abecasis et al. 2012)
HG03753	M4’67	STU	South Asia - South	(Abecasis et al. 2012)
HG04022	M4’67	ITU	South Asia - South	(Abecasis et al. 2012)
HG04235	M4’67	ITU	South Asia - South	(Abecasis et al. 2012)
AY922289	M5	Andhra Pradesh	South Asia - South	(Sun et al. 2006)
FJ383568	M5	Rajasthan	South Asia - West	(Chandrasekar et al. 2009)
FJ383553	M5	Madhya Pradesh	South Asia - Central	(Chandrasekar et al. 2009)
HG02783	M5	PJL	South Asia - West	(Abecasis et al. 2012)
HG03926	M5	BEB	South Asia - East	(Abecasis et al. 2012)
HG03945	M5	STU	South Asia - South	(Abecasis et al. 2012)
HG03991	M5	STU	South Asia - South	(Abecasis et al. 2012)
HG03955	M5	STU	South Asia - South	(Abecasis et al. 2012)
FJ383591	M5	Maharashtra	South Asia - South	(Chandrasekar et al. 2009)
HG03021	M5	PJL	South Asia - West	(Abecasis et al. 2012)
HG03015	M5	PJL	South Asia - West	(Abecasis et al. 2012)
HG02784	M5	PJL	South Asia - West	(Abecasis et al. 2012)
HG02786	M5	PJL	South Asia - West	(Abecasis et al. 2012)
HG02787	M5	PJL	South Asia - West	(Abecasis et al. 2012)
KJ446690	M5	Pakistan: Burusho	South Asia - West	HGDP - Zheng et al. (unpublished)
KF055864	M5	Spain: Romani	Europe	(Gómez-Carballa et al. 2013)
KF055874	M5	Spain: Romani	Europe	(Gómez-Carballa et al. 2013)
KF055888	M5	Spain: Romani	Europe	(Gómez-Carballa et al. 2013)
JQ705991	M5	unknown	unknown	(Behar et al. 2012)
KF055866	M5	Spain: Romani	Europe	(Gómez-Carballa et al. 2013)
KF055876	M5	Spain: Romani	Europe	(Gómez-Carballa et al. 2013)
KF055877	M5	Spain: Romani	Europe	(Gómez-Carballa et al. 2013)
KF055881	M5	Spain: Romani	Europe	(Gómez-Carballa et al. 2013)
KF055883	M5	Spain: Romani	Europe	(Gómez-Carballa et al. 2013)
KF055887	M5	Spain: Romani	Europe	(Gómez-Carballa et al. 2013)
JQ705965	M5	unknown	unknown	(Behar et al. 2012)
NA21124	M5	GIH	South Asia - West	(Abecasis et al. 2012)
NA20878	M5	GIH	South Asia - West	(Abecasis et al. 2012)

Table S1. (*continued*)

Sample	Haplogroup	Origin (Population)	Region	Reference
AY922259	M5	Uttar Pradesh	South Asia - Central	(Sun et al. 2006)
AY922260	M5	Uttar Pradesh	South Asia - Central	(Sun et al. 2006)
HG03814	M5	BEB	South Asia - East	(Abecasis et al. 2012)
FJ383549	M5	Orissa	South Asia - East	(Chandrasekar et al. 2009)
FJ383547	M5	Orissa	South Asia - East	(Chandrasekar et al. 2009)
FJ383544	M5	Orissa	South Asia - East	(Chandrasekar et al. 2009)
FJ383548	M5	Orissa	South Asia - East	(Chandrasekar et al. 2009)
FJ383574	M5	Bihar	South Asia - East	(Chandrasekar et al. 2009)
AY922273	M5	Uttar Pradesh	South Asia - Central	(Sun et al. 2006)
FJ383585	M5	Chhattisgarh	South Asia - Central	(Chandrasekar et al. 2009)
FJ383577	M5	Chhattisgarh	South Asia - Central	(Chandrasekar et al. 2009)
FJ383578	M5	Chhattisgarh	South Asia - Central	(Chandrasekar et al. 2009)
FJ383579	M5	Chhattisgarh	South Asia - Central	(Chandrasekar et al. 2009)
FJ383582	M5	Chhattisgarh	South Asia - Central	(Chandrasekar et al. 2009)
FJ383584	M5	Chhattisgarh	South Asia - Central	(Chandrasekar et al. 2009)
FJ383587	M5	Chhattisgarh	South Asia - Central	(Chandrasekar et al. 2009)
FJ383593	M5	Chhattisgarh	South Asia - Central	(Chandrasekar et al. 2009)
GU480013	M5	Madhya Pradesh	South Asia - Central	(Sharma et al. 2012)
HM030518	M5	China	East Asia	(Kong et al. 2011)
KC911363	M5	Iran: Persian	Southwest Asia	(Derenko et al. 2013)
FJ383564	M5	Rajasthan	South Asia - West	(Chandrasekar et al. 2009)
HG02490	M5	PJL	South Asia - West	(Abecasis et al. 2012)
HG03649	M5	PJL	South Asia - West	(Abecasis et al. 2012)
NA20846	M5	GIH	South Asia - West	(Abecasis et al. 2012)
NA20895	M5	GIH	South Asia - West	(Abecasis et al. 2012)
NA20893	M5	GIH	South Asia - West	(Abecasis et al. 2012)
AY922285	M5	Uttar Pradesh	South Asia - Central	(Sun et al. 2006)
FJ383555	M5	Madhya Pradesh	South Asia - Central	(Chandrasekar et al. 2009)
FJ383556	M5	Madhya Pradesh	South Asia - Central	(Chandrasekar et al. 2009)
HG03624	M5	PJL	South Asia - West	(Abecasis et al. 2012)
KJ446702	M5	Pakistan: Balochi	South Asia - West	HGDP - Zheng et al. (unpublished)
HG04152	M5	BEB	South Asia - East	(Abecasis et al. 2012)
KJ446703	M5	Pakistan: Sindhi	South Asia - West	HGDP - Zheng et al. (unpublished)
FJ770949	M5	India	South Asia	(Fornarino et al. 2009)
HM156673	M5	Andhra Pradesh	South Asia - South	(Govindaraj et al. 2011)
FJ383552	M5	Madhya Pradesh	South Asia - Central	(Chandrasekar et al. 2009)
HG03863	M5	ITU	South Asia - South	(Abecasis et al. 2012)
HG03969	M5	ITU	South Asia - South	(Abecasis et al. 2012)
NA20845	M5	GIH	South Asia - West	(Abecasis et al. 2012)
KJ446693	M5	Pakistan: Balochi	South Asia - West	HGDP - Zheng et al. (unpublished)

Table S1. (*continued*)

Sample	Haplogroup	Origin (Population)	Region	Reference
NA21127	M5	GIH	South Asia - West	(Abecasis et al. 2012)
KJ446695	M5	Pakistan: Brahui	South Asia - West	HGDP - Zheng et al. (unpublished)
KJ446696	M5	Pakistan: Brahui	South Asia - West	HGDP - Zheng et al. (unpublished)
KJ446697	M5	Pakistan: Brahui	South Asia - West	HGDP - Zheng et al. (unpublished)
KJ446698	M5	Pakistan: Balochi	South Asia - West	HGDP - Zheng et al. (unpublished)
KJ446699	M5	Pakistan: Makrani	South Asia - West	HGDP - Zheng et al. (unpublished)
KJ446700	M5	Pakistan: Makrani	South Asia - West	HGDP - Zheng et al. (unpublished)
KJ446701	M5	Pakistan: Brahui	South Asia - West	HGDP - Zheng et al. (unpublished)
HM852834	M5	Iran	Southwest Asia	(Schönberg et al. 2011)
EU597563	M5	Pakistan: Brahui	South Asia - West	(Hartmann et al. 2009)
KC911312	M5	Iran: Persian	Southwest Asia	(Derenko et al. 2013)
KC911579	M5	Iran: Persian	Southwest Asia	(Derenko et al. 2013)
KC911321	M5	Iran: Persian	Southwest Asia	(Derenko et al. 2013)
KC911351	M5	Iran: Persian	Southwest Asia	(Derenko et al. 2013)
KC911557	M5	Iran: Persian	Southwest Asia	(Derenko et al. 2013)
KC911596	M5	Iran: Persian	Southwest Asia	(Derenko et al. 2013)
HM156691	M5	Andhra Pradesh	South Asia - South	(Govindaraj et al. 2011)
FJ383570	M5	Rajasthan	South Asia - West	(Chandrasekar et al. 2009)
FJ383572	M5	Rajasthan	South Asia - West	(Chandrasekar et al. 2009)
FJ383573	M5	Rajasthan	South Asia - West	(Chandrasekar et al. 2009)
FJ383559	M5	Rajasthan	South Asia - West	(Chandrasekar et al. 2009)
FJ383562	M5	Rajasthan	South Asia - West	(Chandrasekar et al. 2009)
FJ383567	M5	Rajasthan	South Asia - West	(Chandrasekar et al. 2009)
FJ383561	M5	Rajasthan	South Asia - West	(Chandrasekar et al. 2009)
FJ383565	M5	Rajasthan	South Asia - West	(Chandrasekar et al. 2009)
FJ383563	M5	Rajasthan	South Asia - West	(Chandrasekar et al. 2009)
FJ383560	M5	Rajasthan	South Asia - West	(Chandrasekar et al. 2009)
FJ383580	M5	Chhattisgarh	South Asia - Central	(Chandrasekar et al. 2009)
FJ383581	M5	Chhattisgarh	South Asia - Central	(Chandrasekar et al. 2009)
FJ383586	M5	Chhattisgarh	South Asia - Central	(Chandrasekar et al. 2009)
FJ383583	M5	Chhattisgarh	South Asia - Central	(Chandrasekar et al. 2009)
FJ383554	M5	Madhya Pradesh	South Asia - Central	(Chandrasekar et al. 2009)
FJ383590	M5	Gujarat	South Asia - West	(Chandrasekar et al. 2009)
NA21125	M5	GIH	South Asia - West	(Abecasis et al. 2012)
FJ383571	M5	Rajasthan	South Asia - West	(Chandrasekar et al. 2009)
FJ383569	M5	Rajasthan	South Asia - West	(Chandrasekar et al. 2009)
FJ383566	M5	Rajasthan	South Asia - West	(Chandrasekar et al. 2009)
EF556195	M5	Kerala: Cochin	South Asia - South	(Behar et al. 2008)

Table S1. (*continued*)

Sample	Haplogroup	Origin (Population)	Region	Reference
KJ446691	M5	Pakistan: Pathan	South Asia - West	HGDP - Zheng et al. (unpublished)
DQ408675	M5	Orissa	South Asia - East	(Thangaraj et al. 2006)
FJ383594	M5	Maharashtra	South Asia - South	(Chandrasekar et al. 2009)
HG03589	M5	BEB	South Asia - East	(Abecasis et al. 2012)
DQ246814	M5	Karnataka	South Asia - South	(Rajkumar et al. 2005)
HG03006	M5	BEB	South Asia - East	(Abecasis et al. 2012)
HG02681	M5	PJL	South Asia - West	(Abecasis et al. 2012)
HG03708	M5	PJL	South Asia - West	(Abecasis et al. 2012)
HG03944	M5	STU	South Asia - South	(Abecasis et al. 2012)
HG03885	M5	STU	South Asia - South	(Abecasis et al. 2012)
HM852843	M5	Iran	Southwest Asia	(Schönberg et al. 2011)
HG04006	M5	STU	South Asia - South	(Abecasis et al. 2012)
KJ446692	M5	Pakistan: Pathan	South Asia - West	HGDP - Zheng et al. (unpublished)
NA21122	M5	GIH	South Asia - West	(Abecasis et al. 2012)
NA21114	M5	GIH	South Asia - West	(Abecasis et al. 2012)
NA20911	M5	GIH	South Asia - West	(Abecasis et al. 2012)
NA21128	M5	GIH	South Asia - West	(Abecasis et al. 2012)
FJ383557	M5	Madhya Pradesh	South Asia - Central	(Chandrasekar et al. 2009)
FJ770955	M5	Nepal	South Asia - Central	(Fornarino et al. 2009)
KC577360	M5	Mauritius	Africa	(Fregel et al. 2014)
FJ383589	M5	Maharashtra	South Asia - South	(Chandrasekar et al. 2009)
FJ383543	M5	Orissa	South Asia - East	(Chandrasekar et al. 2009)
FJ383596	M5	Arunachal Pradesh	South Asia - East	(Chandrasekar et al. 2009)
FJ383595	M5	Arunachal Pradesh	South Asia - East	(Chandrasekar et al. 2009)
FJ383592	M5	Maharashtra	South Asia - South	(Chandrasekar et al. 2009)
FJ383588	M5	Jharkhand	South Asia - East	(Chandrasekar et al. 2009)
HG04094	M5	ITU	South Asia - South	(Abecasis et al. 2012)
FJ383550	M5	Orissa	South Asia - East	(Chandrasekar et al. 2009)
FJ383545	M5	Orissa	South Asia - East	(Chandrasekar et al. 2009)
FJ383576	M5	Bihar	South Asia - East	(Chandrasekar et al. 2009)
HM036533	M5	Great Himalayas: Ladakh tribe	South Asia - Central	Sharma et al. (unpublished)
HM036571	M5	Great Himalayas: Ladakh tribe	South Asia - Central	Sharma et al. (unpublished)
JF742201	M5	Nepal: Kathmandu	South Asia - Central	(Wang et al. 2012)
JF742202	M5	Nepal: Kathmandu	South Asia - Central	(Wang et al. 2012)
KF056269	M5	Sherpa	South Asia - Central	(Kang et al. 2013)
FJ383575	M5	Bihar	South Asia - East	(Chandrasekar et al. 2009)
FJ383551	M5	Jharkhand	South Asia - East	(Chandrasekar et al. 2009)
AY922292	M5	Andhra Pradesh	South Asia - South	(Sun et al. 2006)
AY922281	M5	Uttar Pradesh	South Asia - Central	(Sun et al. 2006)

Table S1. (*continued*)

Sample	Haplogroup	Origin (Population)	Region	Reference
AY922282	M5	Uttar Pradesh	South Asia - Central	(Sun et al. 2006)
HG03971	M5	ITU	South Asia - South	(Abecasis et al. 2012)
NA21143	M5	GIH	South Asia - West	(Abecasis et al. 2012)
FJ383546	M5	Orissa	South Asia - East	(Chandrasekar et al. 2009)
FJ383558	M5	Madhya Pradesh	South Asia - Central	(Chandrasekar et al. 2009)
FJ770953	M5	Nepal	South Asia - Central	(Fornarino et al. 2009)
KF056270	M5	Sherpa	South Asia - Central	(Kang et al. 2013)
DQ246832	M5	West Bengal	South Asia - East	(Rajkumar et al. 2005)
FJ383301	M6	Maharashtra	South Asia - South	(Chandrasekar et al. 2009)
DQ408677	M6	Kerala	South Asia - South	(Thangaraj et al. 2006)
HG02727	M6	PJL	South Asia - West	(Abecasis et al. 2012)
HG04219	M6	ITU	South Asia - South	(Abecasis et al. 2012)
HG02725	M6	PJL	South Asia - West	(Abecasis et al. 2012)
HG02789	M6	PJL	South Asia - West	(Abecasis et al. 2012)
HG02493	M6	PJL	South Asia - West	(Abecasis et al. 2012)
HG02778	M6	PJL	South Asia - West	(Abecasis et al. 2012)
HG03019	M6	PJL	South Asia - West	(Abecasis et al. 2012)
HG03018	M6	PJL	South Asia - West	(Abecasis et al. 2012)
HG03022	M6	PJL	South Asia - West	(Abecasis et al. 2012)
JQ703707	M6	India	South Asia	(Behar et al. 2012)
AY922307	M6	Andhra Pradesh	South Asia - South	(Sun et al. 2006)
HG03774	M6	ITU	South Asia - South	(Abecasis et al. 2012)
HG03713	M6	ITU	South Asia - South	(Abecasis et al. 2012)
HG03722	M6	ITU	South Asia - South	(Abecasis et al. 2012)
HG03756	M6	STU	South Asia - South	(Abecasis et al. 2012)
HG03709	M6	PJL	South Asia - West	(Abecasis et al. 2012)
HG04198	M6	ITU	South Asia - South	(Abecasis et al. 2012)
AY922296	M6	Andhra Pradesh	South Asia - South	(Sun et al. 2006)
FJ383308	M6	Bihar	South Asia - East	(Chandrasekar et al. 2009)
FJ383307	M6	Bihar	South Asia - East	(Chandrasekar et al. 2009)
FJ383306	M6	Bihar	South Asia - East	(Chandrasekar et al. 2009)
FJ383309	M6	Maharashtra	South Asia - South	(Chandrasekar et al. 2009)
FJ383304	M6	Orissa	South Asia - East	(Chandrasekar et al. 2009)
FJ383302	M6	Orissa	South Asia - East	(Chandrasekar et al. 2009)
FJ383303	M6	Orissa	South Asia - East	(Chandrasekar et al. 2009)
HG03977	M6	ITU	South Asia - South	(Abecasis et al. 2012)
HG04206	M6	ITU	South Asia - South	(Abecasis et al. 2012)
HG03986	M6	STU	South Asia - South	(Abecasis et al. 2012)
HG03705	M6	PJL	South Asia - West	(Abecasis et al. 2012)
HG03928	M6	BEB	South Asia - East	(Abecasis et al. 2012)
AY950300	M31	Andaman	South Asia - East	(Thangaraj et al. 2005)
AY950298	M31	Andaman	South Asia - East	(Thangaraj et al. 2005)

Table S1. (*continued*)

Sample	Haplogroup	Origin (Population)	Region	Reference
AY950297	M31	Andaman	South Asia - East	(Thangaraj et al. 2005)
DQ149515	M31	Jarawa	South Asia - East	(Barik et al. 2008)
DQ149516	M31	Jarawa	South Asia - East	(Barik et al. 2008)
DQ149519	M31	Jarawa	South Asia - East	(Barik et al. 2008)
DQ149511	M31	Jarawa	South Asia - East	(Barik et al. 2008)
DQ149520	M31	Jarawa	South Asia - East	(Barik et al. 2008)
AY950293	M31	Andaman	South Asia - East	(Thangaraj et al. 2005)
AY950294	M31	Andaman	South Asia - East	(Thangaraj et al. 2005)
DQ408673	M31	Andaman	South Asia - East	(Thangaraj et al. 2006)
HQ438683	M31	Bangladesh	South Asia - East	(Wang et al. 2011)
HQ438684	M31	Myanmar	Southeast Asia	(Wang et al. 2011)
HQ438687	M31	East India	South Asia - East	(Wang et al. 2011)
HQ438689	M31	East India	South Asia - East	(Wang et al. 2011)
HQ438688	M31	West Bengal	South Asia - East	(Wang et al. 2011)
EU075305	M31	Jharkhand	South Asia - East	(Barik et al. 2008)
EU075306	M31	Jharkhand	South Asia - East	(Barik et al. 2008)
EF060263	M31	Orissa	South Asia - East	(Barik et al. 2008)
EF060264	M31	Orissa	South Asia - East	(Barik et al. 2008)
EF060262	M31	Orissa	South Asia - East	(Barik et al. 2008)
EF060266	M31	Orissa	South Asia - East	(Barik et al. 2008)
EF060265	M31	Orissa	South Asia - East	(Barik et al. 2008)
HQ438685	M31	Northeast India	South Asia - East	(Wang et al. 2011)
WB8	M31	Myanmar	Southeast Asia	This study
WD1	M31	Myanmar	Southeast Asia	This study
HQ438686	M31	West Bengal	South Asia - East	(Wang et al. 2011)
FJ770962	M31	Nepal	South Asia - Central	(Fornarino et al. 2009)
Redd_Bh23	M31	Meghalaya	South Asia - East	Reddy et al. (2007)
DQ149513	M32'56	Jarawa	South Asia - East	(Barik et al. 2008)
DQ149514	M32'56	Jarawa	South Asia - East	(Barik et al. 2008)
DQ149517	M32'56	Jarawa	South Asia - East	(Barik et al. 2008)
DQ149512	M32'56	Jarawa	South Asia - East	(Barik et al. 2008)
DQ149518	M32'56	Jarawa	South Asia - East	(Barik et al. 2008)
AY950291	M32'56	Jarawa	South Asia - East	(Thangaraj et al. 2005)
AY950292	M32'56	Jarawa	South Asia - East	(Thangaraj et al. 2005)
AY950295	M32'56	Jarawa	South Asia - East	(Thangaraj et al. 2005)
AY950296	M32'56	Andaman	South Asia - East	(Thangaraj et al. 2005)
AY950299	M32'56	Andaman	South Asia - East	(Thangaraj et al. 2005)
FJ383487	M32'56	Maharashtra	South Asia - South	(Chandrasekar et al. 2009)
FJ383486	M32'56	Maharashtra	South Asia - South	(Chandrasekar et al. 2009)
FJ770951	M33	India	South Asia	(Fornarino et al. 2009)
FJ383348	M33	Jharkhand	South Asia - East	(Chandrasekar et al. 2009)
FJ383349	M33	Jharkhand	South Asia - East	(Chandrasekar et al. 2009)

Table S1. (*continued*)

Sample	Haplogroup	Origin (Population)	Region	Reference
FJ383350	M33	Jharkhand	South Asia - East	(Chandrasekar et al. 2009)
FJ383346	M33	Jharkhand	South Asia - East	(Chandrasekar et al. 2009)
FJ383347	M33	Jharkhand	South Asia - East	(Chandrasekar et al. 2009)
HG03634	M33	PJL	South Asia - West	(Abecasis et al. 2012)
HG03922	M33	BEB	South Asia - East	(Abecasis et al. 2012)
FJ770963	M33	Nepal	South Asia - Central	(Fornarino et al. 2009)
JF742205	M33	Nepal: Kathmandu	South Asia - Central	(Wang et al. 2012)
FJ383344	M33	Sikkim	South Asia - East	(Chandrasekar et al. 2009)
FJ383340	M33	Sikkim	South Asia - East	(Chandrasekar et al. 2009)
FJ383341	M33	Sikkim	South Asia - East	(Chandrasekar et al. 2009)
FJ383339	M33	Sikkim	South Asia - East	(Chandrasekar et al. 2009)
FJ383342	M33	Sikkim	South Asia - East	(Chandrasekar et al. 2009)
FJ383343	M33	Sikkim	South Asia - East	(Chandrasekar et al. 2009)
FJ383345	M33	Sikkim	South Asia - East	(Chandrasekar et al. 2009)
DQ408680	M33	Gujarat	South Asia - West	(Thangaraj et al. 2006)
FJ383358	M33	Rajasthan	South Asia - West	(Chandrasekar et al. 2009)
FJ383353	M33	Rajasthan	South Asia - West	(Chandrasekar et al. 2009)
FJ383354	M33	Rajasthan	South Asia - West	(Chandrasekar et al. 2009)
FJ383356	M33	Rajasthan	South Asia - West	(Chandrasekar et al. 2009)
FJ383355	M33	Rajasthan	South Asia - West	(Chandrasekar et al. 2009)
FJ383352	M33	Rajasthan	South Asia - West	(Chandrasekar et al. 2009)
FJ383357	M33	Rajasthan	South Asia - West	(Chandrasekar et al. 2009)
FJ383361	M33	Maharashtra	South Asia - South	(Chandrasekar et al. 2009)
HG03642	M33	STU	South Asia - South	(Abecasis et al. 2012)
JQ702439	M33	Qatar	Southwest Asia	(Behar et al. 2012)
AY922276	M33	Uttar Pradesh	South Asia - Central	(Sun et al. 2006)
FJ383338	M33	Madhya Pradesh	South Asia - Central	(Chandrasekar et al. 2009)
FJ383360	M33	Madhya Pradesh	South Asia - Central	(Chandrasekar et al. 2009)
HG03989	M33	STU	South Asia - South	(Abecasis et al. 2012)
JN540042	M33	Iraq: Marsh Arab	Southwest Asia	(Al-Zahery et al. 2011)
JX289104	M33	Myanmar	Southeast Asia	(Summerer et al. 2014)
FJ383359	M33	Assam	South Asia - East	(Chandrasekar et al. 2009)
FJ770950	M33	India	South Asia	(Fornarino et al. 2009)
HG02660	M33	PJL	South Asia - West	(Abecasis et al. 2012)
FJ770960	M33	Nepal	South Asia - Central	(Fornarino et al. 2009)
HG04202	M33	ITU	South Asia - South	(Abecasis et al. 2012)
Redd_Ga11	M33	Meghalaya	South Asia - East	Reddy et al. (2007)
JF742198	M33	Nepal: Kathmandu	South Asia - Central	(Wang et al. 2012)
AY922298	M33	West Bengal	South Asia - East	(Sun et al. 2006)
FJ383351	M33	Chhattisgarh	South Asia - Central	(Chandrasekar et al. 2009)
HG04171	M33	BEB	South Asia - East	(Abecasis et al. 2012)
FJ383362	M33	Arunachal Pradesh	South Asia - East	(Chandrasekar et al. 2009)

Table S1. (*continued*)

Sample	Haplogroup	Origin (Population)	Region	Reference
Redd_Pn14	M33	Arunachal Pradesh	South Asia - East	Reddy et al. (2007)
FJ770968	M33	Nepal	South Asia - Central	(Fornarino et al. 2009)
KC252506	M33	Taiwan	East Asia	(Loo et al. 2014)
JQ702003	M33	unknown	unknown	(Behar et al. 2012)
KC252508	M33	Taiwan	East Asia	(Loo et al. 2014)
AY922274	M34'57	Uttar Pradesh	South Asia - Central	(Sun et al. 2006)
FJ383681	M34'57	Madhya Pradesh	South Asia - Central	(Chandrasekar et al. 2009)
HG03884	M34'57	STU	South Asia - South	(Abecasis et al. 2012)
FJ383682	M34'57	Orissa	South Asia - East	(Chandrasekar et al. 2009)
FJ383680	M34'57	Bihar	South Asia - East	(Chandrasekar et al. 2009)
HG04153	M34'57	BEB	South Asia - East	(Abecasis et al. 2012)
DQ408672	M34'57	Karnataka	South Asia - South	(Thangaraj et al. 2006)
AY922304	M34'57	Uttar Pradesh	South Asia - Central	(Sun et al. 2006)
JX289117	M34'57	Myanmar	Southeast Asia	(Summerer et al. 2014)
FJ383764	M34'57	Madhya Pradesh	South Asia - Central	(Chandrasekar et al. 2009)
KJ446727	M34'57	Pakistan: Sindhi	South Asia - West	HGDP - Zheng et al. (unpublished)
FJ383763	M34'57	Maharashtra	South Asia - South	(Chandrasekar et al. 2009)
FJ383762	M34'57	Maharashtra	South Asia - South	(Chandrasekar et al. 2009)
FJ383760	M34'57	Maharashtra	South Asia - South	(Chandrasekar et al. 2009)
FJ383761	M34'57	Maharashtra	South Asia - South	(Chandrasekar et al. 2009)
FJ383757	M34'57	Rajasthan	South Asia - West	(Chandrasekar et al. 2009)
FJ383759	M34'57	Rajasthan	South Asia - West	(Chandrasekar et al. 2009)
FJ383758	M34'57	Rajasthan	South Asia - West	(Chandrasekar et al. 2009)
FJ383380	M35	Madhya Pradesh	South Asia - Central	(Chandrasekar et al. 2009)
HG04185	M35	BEB	South Asia - East	(Abecasis et al. 2012)
FJ770952	M35	India	South Asia	(Fornarino et al. 2009)
FJ383391	M35	Karnataka	South Asia - South	(Chandrasekar et al. 2009)
FJ383384	M35	Karnataka	South Asia - South	(Chandrasekar et al. 2009)
FJ383387	M35	Karnataka	South Asia - South	(Chandrasekar et al. 2009)
FJ383388	M35	Karnataka	South Asia - South	(Chandrasekar et al. 2009)
FJ383385	M35	Karnataka	South Asia - South	(Chandrasekar et al. 2009)
FJ383389	M35	Karnataka	South Asia - South	(Chandrasekar et al. 2009)
FJ383386	M35	Karnataka	South Asia - South	(Chandrasekar et al. 2009)
FJ383390	M35	Karnataka	South Asia - South	(Chandrasekar et al. 2009)
AY289074	M35	Mullukurunan	South Asia - South	(Ingman and Gyllensten 2003)
HG03800	M35	BEB	South Asia - East	(Abecasis et al. 2012)
HG03731	M35	ITU	South Asia - South	(Abecasis et al. 2012)
HG03869	M35	ITU	South Asia - South	(Abecasis et al. 2012)
HG03697	M35	STU	South Asia - South	(Abecasis et al. 2012)
HG03882	M35	ITU	South Asia - South	(Abecasis et al. 2012)
HG04062	M35	ITU	South Asia - South	(Abecasis et al. 2012)

Table S1. (*continued*)

Sample	Haplogroup	Origin (Population)	Region	Reference
HM156693	M35	Andhra Pradesh	South Asia - South	(Govindaraj et al. 2011)
FJ383365	M35	Maharashtra	South Asia - South	(Chandrasekar et al. 2009)
FJ383364	M35	Maharashtra	South Asia - South	(Chandrasekar et al. 2009)
AY922264	M35	Andhra Pradesh	South Asia - South	(Sun et al. 2006)
FJ383368	M35	Rajasthan	South Asia - West	(Chandrasekar et al. 2009)
AY922279	M35	Andhra Pradesh	South Asia - South	(Sun et al. 2006)
FJ383363	M35	Maharashtra	South Asia - South	(Chandrasekar et al. 2009)
FJ383372	M35	Maharashtra	South Asia - South	(Chandrasekar et al. 2009)
AY922272	M35	Andhra Pradesh	South Asia - South	(Sun et al. 2006)
HG03856	M35	STU	South Asia - South	(Abecasis et al. 2012)
HG04059	M35	ITU	South Asia - South	(Abecasis et al. 2012)
NA21116	M35	GIH	South Asia - West	(Abecasis et al. 2012)
HG03832	M35	BEB	South Asia - East	(Abecasis et al. 2012)
FJ383393	M35	Assam	South Asia - East	(Chandrasekar et al. 2009)
FJ383369	M35	Assam	South Asia - East	(Chandrasekar et al. 2009)
FJ383370	M35	Assam	South Asia - East	(Chandrasekar et al. 2009)
FJ383371	M35	Assam	South Asia - East	(Chandrasekar et al. 2009)
NA20869	M35	GIH	South Asia - West	(Abecasis et al. 2012)
NA20876	M35	GIH	South Asia - West	(Abecasis et al. 2012)
JF742208	M35	Nepal	South Asia - Central	(Wang et al. 2012)
HG03615	M35	BEB	South Asia - East	(Abecasis et al. 2012)
FJ383377	M35	Chhattisgarh	South Asia - Central	(Chandrasekar et al. 2009)
FJ383374	M35	Chhattisgarh	South Asia - Central	(Chandrasekar et al. 2009)
FJ383375	M35	Chhattisgarh	South Asia - Central	(Chandrasekar et al. 2009)
FJ383378	M35	Chhattisgarh	South Asia - Central	(Chandrasekar et al. 2009)
FJ383379	M35	Chhattisgarh	South Asia - Central	(Chandrasekar et al. 2009)
FJ383376	M35	Chhattisgarh	South Asia - Central	(Chandrasekar et al. 2009)
HM156677	M35	Andhra Pradesh	South Asia - South	(Govindaraj et al. 2011)
HG04070	M35	ITU	South Asia - South	(Abecasis et al. 2012)
HG04020	M35	ITU	South Asia - South	(Abecasis et al. 2012)
HM036544	M35	Great Himalayas: Ladakh tribe	South Asia - Central	Sharma et al. (unpublished)
HM036535	M35	Great Himalayas: Ladakh tribe	South Asia - Central	Sharma et al. (unpublished)
HM036559	M35	Great Himalayas: Ladakh tribe	South Asia - Central	Sharma et al. (unpublished)
HM036575	M35	Great Himalayas: Ladakh tribe	South Asia - Central	Sharma et al. (unpublished)
FJ770966	M35	Nepal	South Asia - Central	(Fornarino et al. 2009)
FJ383392	M35	Arunachal Pradesh	South Asia - East	(Chandrasekar et al. 2009)
GU810066	M35	Thailand	Southeast Asia	Pradutkanchana et al. (unpublished)
JF742209	M35	Nepal	South Asia - Central	(Wang et al. 2012)
FJ383381	M35	Gujarat	South Asia - West	(Chandrasekar et al. 2009)

Table S1. (*continued*)

Sample	Haplogroup	Origin (Population)	Region	Reference
FJ383373	M35	Bihar	South Asia - East	(Chandrasekar et al. 2009)
JF742215	M35	Nepal	South Asia - Central	(Wang et al. 2012)
FJ383383	M35	Gujarat	South Asia - West	(Chandrasekar et al. 2009)
FJ383382	M35	Gujarat	South Asia - West	(Chandrasekar et al. 2009)
FJ383366	M35	Maharashtra	South Asia - South	(Chandrasekar et al. 2009)
FJ383367	M35	Maharashtra	South Asia - South	(Chandrasekar et al. 2009)
GU480006	M35	Madhya Pradesh	South Asia - Central	(Sharma et al. 2012)
HG04216	M35	ITU	South Asia - South	(Abecasis et al. 2012)
HG02601	M35	PJL	South Asia - West	(Abecasis et al. 2012)
HG03917	M35	BEB	South Asia - East	(Abecasis et al. 2012)
FJ770956	M35	Nepal	South Asia - Central	(Fornarino et al. 2009)
AY922288	M39	Uttar Pradesh	South Asia - Central	(Sun et al. 2006)
AY922269	M39	Andhra Pradesh	South Asia - South	(Sun et al. 2006)
AY922293	M39	Andhra Pradesh	South Asia - South	(Sun et al. 2006)
HG04209	M39	ITU	South Asia - South	(Abecasis et al. 2012)
FJ383714	M39	Maharashtra	South Asia - South	(Chandrasekar et al. 2009)
FJ383710	M39	Chhattisgarh	South Asia - Central	(Chandrasekar et al. 2009)
FJ383715	M39	Maharashtra	South Asia - South	(Chandrasekar et al. 2009)
FJ383705	M39	Bihar	South Asia - East	(Chandrasekar et al. 2009)
FJ383712	M39	Jharkhand	South Asia - East	(Chandrasekar et al. 2009)
FJ383706	M39	Bihar	South Asia - East	(Chandrasekar et al. 2009)
FJ383702	M39	Orissa	South Asia - East	(Chandrasekar et al. 2009)
FJ383708	M39	Maharashtra	South Asia - South	(Chandrasekar et al. 2009)
FJ383713	M39	Maharashtra	South Asia - South	(Chandrasekar et al. 2009)
FJ383707	M39	Maharashtra	South Asia - South	(Chandrasekar et al. 2009)
FJ383709	M39	Maharashtra	South Asia - South	(Chandrasekar et al. 2009)
FJ383703	M39	Chhattisgarh	South Asia - Central	(Chandrasekar et al. 2009)
FJ383711	M39	Chhattisgarh	South Asia - Central	(Chandrasekar et al. 2009)
AY922262	M39	Andhra Pradesh	South Asia - South	(Sun et al. 2006)
HG03870	M39	ITU	South Asia - South	(Abecasis et al. 2012)
HG03838	M39	STU	South Asia - South	(Abecasis et al. 2012)
AY922275	M39	Andhra Pradesh	South Asia - South	(Sun et al. 2006)
NA20897	M39	GIH	South Asia - West	(Abecasis et al. 2012)
HG02597	M39	PJL	South Asia - West	(Abecasis et al. 2012)
NA20888	M39	GIH	South Asia - West	(Abecasis et al. 2012)
NA20906	M39	GIH	South Asia - West	(Abecasis et al. 2012)
NA20908	M39	GIH	South Asia - West	(Abecasis et al. 2012)
FJ383704	M39	Maharashtra	South Asia - South	(Chandrasekar et al. 2009)
FJ383701	M39	Maharashtra	South Asia - South	(Chandrasekar et al. 2009)
FJ383699	M39	Karnataka	South Asia - South	(Chandrasekar et al. 2009)
FJ383697	M39	Karnataka	South Asia - South	(Chandrasekar et al. 2009)
FJ383696	M39	Karnataka	South Asia - South	(Chandrasekar et al. 2009)

Table S1. (*continued*)

Sample	Haplogroup	Origin (Population)	Region	Reference
FJ383698	M39	Karnataka	South Asia - South	(Chandrasekar et al. 2009)
FJ383700	M39	Karnataka	South Asia - South	(Chandrasekar et al. 2009)
HG03787	M39	ITU	South Asia - South	(Abecasis et al. 2012)
HG03963	M39	ITU	South Asia - South	(Abecasis et al. 2012)
AY922287	M40	Andhra Pradesh	South Asia - South	(Sun et al. 2006)
NA20864	M40	GIH	South Asia - West	(Abecasis et al. 2012)
HG03896	M40	STU	South Asia - South	(Abecasis et al. 2012)
HG04075	M40	STU	South Asia - South	(Abecasis et al. 2012)
HG04015	M40	ITU	South Asia - South	(Abecasis et al. 2012)
AY922284	M40	Andhra Pradesh	South Asia - South	(Sun et al. 2006)
FJ383807	M40	Chhattisgarh	South Asia - Central	(Chandrasekar et al. 2009)
FJ383786	M40	Karnataka	South Asia - South	(Chandrasekar et al. 2009)
FJ383784	M40	Karnataka	South Asia - South	(Chandrasekar et al. 2009)
FJ383785	M40	Karnataka	South Asia - South	(Chandrasekar et al. 2009)
FJ383796	M40	Karnataka	South Asia - South	(Chandrasekar et al. 2009)
FJ383797	M40	Karnataka	South Asia - South	(Chandrasekar et al. 2009)
FJ383802	M40	Karnataka	South Asia - South	(Chandrasekar et al. 2009)
FJ383789	M40	Karnataka	South Asia - South	(Chandrasekar et al. 2009)
FJ383805	M40	Karnataka	South Asia - South	(Chandrasekar et al. 2009)
FJ383793	M40	Karnataka	South Asia - South	(Chandrasekar et al. 2009)
FJ383804	M40	Karnataka	South Asia - South	(Chandrasekar et al. 2009)
FJ383782	M40	Karnataka	South Asia - South	(Chandrasekar et al. 2009)
FJ383783	M40	Karnataka	South Asia - South	(Chandrasekar et al. 2009)
FJ383780	M40	Karnataka	South Asia - South	(Chandrasekar et al. 2009)
FJ383806	M40	Karnataka	South Asia - South	(Chandrasekar et al. 2009)
FJ383779	M40	Karnataka	South Asia - South	(Chandrasekar et al. 2009)
FJ383794	M40	Karnataka	South Asia - South	(Chandrasekar et al. 2009)
FJ383798	M40	Karnataka	South Asia - South	(Chandrasekar et al. 2009)
FJ383790	M40	Karnataka	South Asia - South	(Chandrasekar et al. 2009)
FJ383803	M40	Karnataka	South Asia - South	(Chandrasekar et al. 2009)
FJ383778	M40	Karnataka	South Asia - South	(Chandrasekar et al. 2009)
FJ383781	M40	Karnataka	South Asia - South	(Chandrasekar et al. 2009)
FJ383787	M40	Karnataka	South Asia - South	(Chandrasekar et al. 2009)
FJ383788	M40	Karnataka	South Asia - South	(Chandrasekar et al. 2009)
FJ383800	M40	Karnataka	South Asia - South	(Chandrasekar et al. 2009)
FJ383791	M40	Karnataka	South Asia - South	(Chandrasekar et al. 2009)
FJ383792	M40	Karnataka	South Asia - South	(Chandrasekar et al. 2009)
FJ383808	M40	Karnataka	South Asia - South	(Chandrasekar et al. 2009)
FJ383811	M40	Karnataka	South Asia - South	(Chandrasekar et al. 2009)
FJ383809	M40	Karnataka	South Asia - South	(Chandrasekar et al. 2009)
FJ383795	M40	Karnataka	South Asia - South	(Chandrasekar et al. 2009)
FJ383799	M40	Karnataka	South Asia - South	(Chandrasekar et al. 2009)

Table S1. (*continued*)

Sample	Haplogroup	Origin (Population)	Region	Reference
FJ383801	M40	Karnataka	South Asia - South	(Chandrasekar et al. 2009)
FJ383810	M40	Karnataka	South Asia - South	(Chandrasekar et al. 2009)
FJ383409	M40	Chhattisgarh	South Asia - Central	(Chandrasekar et al. 2009)
FJ383406	M40	Jharkhand	South Asia - East	(Chandrasekar et al. 2009)
NA20874	M40	GIH	South Asia - West	(Abecasis et al. 2012)
FJ383425	M40	Bihar	South Asia - East	(Chandrasekar et al. 2009)
FJ383413	M40	Madhya Pradesh	South Asia - Central	(Chandrasekar et al. 2009)
FJ383423	M40	Madhya Pradesh	South Asia - Central	(Chandrasekar et al. 2009)
FJ383421	M40	Bihar	South Asia - East	(Chandrasekar et al. 2009)
FJ383426	M40	Bihar	South Asia - East	(Chandrasekar et al. 2009)
FJ383427	M40	Bihar	South Asia - East	(Chandrasekar et al. 2009)
FJ383422	M40	Bihar	South Asia - East	(Chandrasekar et al. 2009)
FJ383424	M40	Bihar	South Asia - East	(Chandrasekar et al. 2009)
HG04180	M40	BEB	South Asia - East	(Abecasis et al. 2012)
FJ383419	M40	Orissa	South Asia - East	(Chandrasekar et al. 2009)
FJ383420	M40	Orissa	South Asia - East	(Chandrasekar et al. 2009)
FJ383417	M40	Orissa	South Asia - East	(Chandrasekar et al. 2009)
FJ383414	M40	Orissa	South Asia - East	(Chandrasekar et al. 2009)
FJ383412	M40	Arunachal Pradesh	South Asia - East	(Chandrasekar et al. 2009)
FJ383410	M40	Arunachal Pradesh	South Asia - East	(Chandrasekar et al. 2009)
FJ383411	M40	Arunachal Pradesh	South Asia - East	(Chandrasekar et al. 2009)
FJ383407	M40	Madhya Pradesh	South Asia - Central	(Chandrasekar et al. 2009)
FJ383408	M40	Madhya Pradesh	South Asia - Central	(Chandrasekar et al. 2009)
HG03771	M40	ITU	South Asia - South	(Abecasis et al. 2012)
FJ383418	M40	Orissa	South Asia - East	(Chandrasekar et al. 2009)
FJ383416	M40	Orissa	South Asia - East	(Chandrasekar et al. 2009)
FJ383415	M40	Orissa	South Asia - East	(Chandrasekar et al. 2009)
AY922295	M40	Andhra Pradesh	South Asia - South	(Sun et al. 2006)
AY922271	M40	Andhra Pradesh	South Asia - South	(Sun et al. 2006)
AY922294	M40	Andhra Pradesh	South Asia - South	(Sun et al. 2006)
AY922280	M41	Andhra Pradesh	South Asia - South	(Sun et al. 2006)
FJ383718	M41	Jharkhand	South Asia - East	(Chandrasekar et al. 2009)
FJ383717	M41	Jharkhand	South Asia - East	(Chandrasekar et al. 2009)
FJ383716	M41	Jharkhand	South Asia - East	(Chandrasekar et al. 2009)
FJ383719	M41	Jharkhand	South Asia - East	(Chandrasekar et al. 2009)
HG03593	M41	BEB	South Asia - East	(Abecasis et al. 2012)
DQ513521	M41	Andhra Pradesh	South Asia - South	(Thangaraj et al. 2006)
FJ383722	M41	Chhattisgarh	South Asia - Central	(Chandrasekar et al. 2009)
FJ383721	M41	Chhattisgarh	South Asia - Central	(Chandrasekar et al. 2009)
FJ383720	M41	Chhattisgarh	South Asia - Central	(Chandrasekar et al. 2009)
GU480001	M41	Madhya Pradesh	South Asia - Central	(Sharma et al. 2012)
DQ513522	M41	Andhra Pradesh	South Asia - South	(Thangaraj et al. 2006)

Table S1. (*continued*)

Sample	Haplogroup	Origin (Population)	Region	Reference
FJ380216	M42b	Orissa	South Asia - East	(Kumar et al. 2009)
KC911559	M42b	Iran: Persian	Southwest Asia	(Derenko et al. 2013)
FJ380213	M42b	Jharkhand	South Asia - East	(Kumar et al. 2009)
HG03858	M42b	STU	South Asia - South	(Abecasis et al. 2012)
FJ383742	M42b	Chhattisgarh	South Asia - Central	(Chandrasekar et al. 2009)
FJ380210	M42b	Chhattisgarh	South Asia - Central	(Kumar et al. 2009)
FJ383743	M42b	Chhattisgarh	South Asia - Central	(Chandrasekar et al. 2009)
FJ380211	M42b	Chhattisgarh	South Asia - Central	(Kumar et al. 2009)
FJ380212	M42b	Chhattisgarh	South Asia - Central	(Kumar et al. 2009)
FJ383745	M42b	Bihar	South Asia - East	(Chandrasekar et al. 2009)
FJ380214	M42b	Jharkhand	South Asia - East	(Kumar et al. 2009)
FJ383746	M42b	Bihar	South Asia - East	(Chandrasekar et al. 2009)
FJ380215	M42b	Jharkhand	South Asia - East	(Kumar et al. 2009)
FJ383750	M44	Maharashtra	South Asia - South	(Chandrasekar et al. 2009)
FJ383751	M44	Maharashtra	South Asia - South	(Chandrasekar et al. 2009)
FJ383753	M44	Maharashtra	South Asia - South	(Chandrasekar et al. 2009)
FJ383755	M44	Maharashtra	South Asia - South	(Chandrasekar et al. 2009)
FJ383754	M44	Maharashtra	South Asia - South	(Chandrasekar et al. 2009)
FJ383752	M44	Maharashtra	South Asia - South	(Chandrasekar et al. 2009)
FJ383756	M44	Maharashtra	South Asia - South	(Chandrasekar et al. 2009)
FJ383749	M44	Maharashtra	South Asia - South	(Chandrasekar et al. 2009)
HG03740	M44	STU	South Asia - South	(Abecasis et al. 2012)
AY922253	M44	Uttar Pradesh	South Asia - Central	(Sun et al. 2006)
FJ383748	M44	Maharashtra	South Asia - South	(Chandrasekar et al. 2009)
FJ383747	M44	Maharashtra	South Asia - South	(Chandrasekar et al. 2009)
Redd_Kh52	M48	Meghalaya	South Asia - East	Reddy et al. (2007)
JX289133	M49	Myanmar	Southeast Asia	(Summerer et al. 2014)
WI9	M49	Myanmar	Southeast Asia	This study
JX289094	M49	Myanmar	Southeast Asia	(Summerer et al. 2014)
JX289099	M49	Myanmar	Southeast Asia	(Summerer et al. 2014)
Redd_Bh1	M49	Meghalaya	South Asia - East	Reddy et al. (2007)
FJ383735	M49	Arunachal Pradesh	South Asia - East	(Chandrasekar et al. 2009)
FJ383724	M49	Arunachal Pradesh	South Asia - East	(Chandrasekar et al. 2009)
FJ383732	M49	Arunachal Pradesh	South Asia - East	(Chandrasekar et al. 2009)
FJ383726	M49	Arunachal Pradesh	South Asia - East	(Chandrasekar et al. 2009)
FJ383727	M49	Arunachal Pradesh	South Asia - East	(Chandrasekar et al. 2009)
FJ383728	M49	Arunachal Pradesh	South Asia - East	(Chandrasekar et al. 2009)
FJ383730	M49	Arunachal Pradesh	South Asia - East	(Chandrasekar et al. 2009)
FJ383731	M49	Arunachal Pradesh	South Asia - East	(Chandrasekar et al. 2009)
FJ383729	M49	Arunachal Pradesh	South Asia - East	(Chandrasekar et al. 2009)
FJ383725	M49	Arunachal Pradesh	South Asia - East	(Chandrasekar et al. 2009)
FJ383723	M49	Arunachal Pradesh	South Asia - East	(Chandrasekar et al. 2009)

Table S1. (*continued*)

Sample	Haplogroup	Origin (Population)	Region	Reference
FJ383734	M49	Arunachal Pradesh	South Asia - East	(Chandrasekar et al. 2009)
FJ383733	M49	Arunachal Pradesh	South Asia - East	(Chandrasekar et al. 2009)
JX289106	M49	Myanmar	Southeast Asia	(Summerer et al. 2014)
KM267721	M49	India	South Asia	(Palanichamy et al. 2014)
JQ702407	M49	unknown	unknown	(Behar et al. 2012)
FJ383768	M49	Arunachal Pradesh	South Asia - East	(Chandrasekar et al. 2009)
FJ383767	M49	Arunachal Pradesh	South Asia - East	(Chandrasekar et al. 2009)
KM267720	M49	India	South Asia	(Palanichamy et al. 2014)
FJ383766	M49	Orissa	South Asia - East	(Chandrasekar et al. 2009)
HG02685	M49	PJL	South Asia - West	(Abecasis et al. 2012)
HG03595	M49	BEB	South Asia - East	(Abecasis et al. 2012)
KM267722	M49	India	South Asia	(Palanichamy et al. 2014)
KC577354	M49	Mauritius	Africa	(Fregel et al. 2014)
HG03967	M49	ITU	South Asia - South	(Abecasis et al. 2012)
FJ383765	M49	Jharkhand	South Asia - East	(Chandrasekar et al. 2009)
FJ770940	M49	Andhra Pradesh	South Asia - South	(Fornarino et al. 2009)
GU480011	M49	Madhya Pradesh	South Asia - Central	(Sharma et al. 2012)
Redd_Kh91	M50	Meghalaya	South Asia - East	Reddy et al. (2007)
HG03796	M50	BEB	South Asia - East	(Abecasis et al. 2012)
HG04195	M50	BEB	South Asia - East	(Abecasis et al. 2012)
AY922299	M52	Andhra Pradesh	South Asia - South	(Sun et al. 2006)
JF742203	M52	Nepal: Kathmandu	South Asia - Central	(Wang et al. 2012)
FJ770964	M52	Nepal	South Asia - Central	(Fornarino et al. 2009)
HG03652	M52	PJL	South Asia - West	(Abecasis et al. 2012)
FJ383488	M52	Bihar	South Asia - East	(Chandrasekar et al. 2009)
FJ383490	M52	Bihar	South Asia - East	(Chandrasekar et al. 2009)
FJ383489	M52	Bihar	South Asia - East	(Chandrasekar et al. 2009)
FJ383491	M52	Bihar	South Asia - East	(Chandrasekar et al. 2009)
FJ383492	M52	Madhya Pradesh	South Asia - Central	(Chandrasekar et al. 2009)
JQ703445	M52	unknown	unknown	(Behar et al. 2012)
HG03234	M52	PJL	South Asia - West	(Abecasis et al. 2012)
HG03898	M52	STU	South Asia - South	(Abecasis et al. 2012)
HG03899	M52	STU	South Asia - South	(Abecasis et al. 2012)
HG03733	M52	STU	South Asia - South	(Abecasis et al. 2012)
HG03775	M52	ITU	South Asia - South	(Abecasis et al. 2012)
FJ157842	M52	Uttar Pradesh: Shia Muslim	South Asia - Central	(Eaaswarkhanth et al. 2010)
FJ157848	M52	Uttar Pradesh: Sunni Muslim	South Asia - Central	(Eaaswarkhanth et al. 2010)
FJ157844	M52	Uttar Pradesh: Shia Muslim	South Asia - Central	(Eaaswarkhanth et al. 2010)
FJ157845	M52	Uttar Pradesh: Shia Muslim	South Asia - Central	(Eaaswarkhanth et al. 2010)

Table S1. (*continued*)

Sample	Haplogroup	Origin (Population)	Region	Reference
FJ157841	M52	Uttar Pradesh: Shia Muslim	South Asia - Central	(Eaaswarkhanth et al. 2010)
FJ157846	M52	Uttar Pradesh: Shia Muslim	South Asia - Central	(Eaaswarkhanth et al. 2010)
HM852830	M52	Iran	Southwest Asia	(Schönberg et al. 2011)
FJ157847	M52	Uttar Pradesh: Sunni Muslim	South Asia - Central	(Eaaswarkhanth et al. 2010)
FJ157849	M52	Uttar Pradesh: Sunni Muslim	South Asia - Central	(Eaaswarkhanth et al. 2010)
FJ157843	M52	Uttar Pradesh: Shia Muslim	South Asia - Central	(Eaaswarkhanth et al. 2010)
FJ383444	M53	Chhattisgarh	South Asia - Central	(Chandrasekar et al. 2009)
FJ383446	M53	Chhattisgarh	South Asia - Central	(Chandrasekar et al. 2009)
FJ383443	M53	Chhattisgarh	South Asia - Central	(Chandrasekar et al. 2009)
FJ383448	M53	Chhattisgarh	South Asia - Central	(Chandrasekar et al. 2009)
FJ383442	M53	Chhattisgarh	South Asia - Central	(Chandrasekar et al. 2009)
FJ383455	M53	Orissa	South Asia - East	(Chandrasekar et al. 2009)
FJ383456	M53	Orissa	South Asia - East	(Chandrasekar et al. 2009)
FJ383457	M53	Orissa	South Asia - East	(Chandrasekar et al. 2009)
FJ383458	M53	Orissa	South Asia - East	(Chandrasekar et al. 2009)
FJ383449	M53	Madhya Pradesh	South Asia - Central	(Chandrasekar et al. 2009)
FJ383450	M53	Madhya Pradesh	South Asia - Central	(Chandrasekar et al. 2009)
FJ383451	M53	Madhya Pradesh	South Asia - Central	(Chandrasekar et al. 2009)
FJ383452	M53	Madhya Pradesh	South Asia - Central	(Chandrasekar et al. 2009)
FJ383453	M53	Madhya Pradesh	South Asia - Central	(Chandrasekar et al. 2009)
FJ383454	M53	Madhya Pradesh	South Asia - Central	(Chandrasekar et al. 2009)
FJ383440	M53	Chhattisgarh	South Asia - Central	(Chandrasekar et al. 2009)
FJ383438	M53	Chhattisgarh	South Asia - Central	(Chandrasekar et al. 2009)
FJ383445	M53	Chhattisgarh	South Asia - Central	(Chandrasekar et al. 2009)
FJ383441	M53	Chhattisgarh	South Asia - Central	(Chandrasekar et al. 2009)
FJ383439	M53	Chhattisgarh	South Asia - Central	(Chandrasekar et al. 2009)
FJ383447	M53	Chhattisgarh	South Asia - Central	(Chandrasekar et al. 2009)
AY922302	M53	Andhra Pradesh	South Asia - South	(Sun et al. 2006)
HG03757	M53	STU	South Asia - South	(Abecasis et al. 2012)
FJ383493	M58	Arunachal Pradesh	South Asia - East	(Chandrasekar et al. 2009)
WA10	M58	Myanmar	Southeast Asia	This study
FJ383305	M6	Madhya Pradesh	South Asia - Central	(Chandrasekar et al. 2009)
FJ383498	M60	Arunachal Pradesh	South Asia - East	(Chandrasekar et al. 2009)
FJ383494	M60	Arunachal Pradesh	South Asia - East	(Chandrasekar et al. 2009)
FJ383496	M60	Arunachal Pradesh	South Asia - East	(Chandrasekar et al. 2009)
FJ383495	M60	Arunachal Pradesh	South Asia - East	(Chandrasekar et al. 2009)
FJ383497	M60	Arunachal Pradesh	South Asia - East	(Chandrasekar et al. 2009)
PRY39	M60	Indonesia: South Borneo	Southeast Asia	This study
JF742204	M60	Nepal: Kathmandu	South Asia - Central	(Wang et al. 2012)

Table S1. (*continued*)

Sample	Haplogroup	Origin (Population)	Region	Reference
FJ383500	M60	Assam	South Asia - East	(Chandrasekar et al. 2009)
FJ383499	M60	Assam	South Asia - East	(Chandrasekar et al. 2009)
FJ383501	M62	unknown	unknown	(Chandrasekar et al. 2009)
FJ383502	M62	Arunachal Pradesh	South Asia - East	(Chandrasekar et al. 2009)
FJ748717	M62	China	East Asia	(Ji et al. 2012)
HG04238	N1a2	ITU	South Asia - South	(Abecasis et al. 2012)
HG03698	N1a2	STU	South Asia - South	(Abecasis et al. 2012)
HG04047	N5	STU	South Asia - South	(Abecasis et al. 2012)
AY714031	N5	Andhrapradesh: Reddy	South Asia - South	(Palanichamy et al. 2004)
GU480015	N5	Madhya Pradesh	South Asia - Central	(Sharma et al. 2012)
GU480016	N5	Madhya Pradesh	South Asia - Central	(Sharma et al. 2012)
GU480019	N5	Madhya Pradesh	South Asia - Central	(Sharma et al. 2012)
GU480020	N5	Madhya Pradesh	South Asia - Central	(Sharma et al. 2012)
GU480021	N5	Madhya Pradesh	South Asia - Central	(Sharma et al. 2012)
HG03943	N21	STU	South Asia - South	(Abecasis et al. 2012)
HG02790	R	PJL	South Asia - West	(Abecasis et al. 2012)
HG03706	R	PJL	South Asia - West	(Abecasis et al. 2012)
HG02774	R	PJL	South Asia - West	(Abecasis et al. 2012)
HG02696	R	PJL	South Asia - West	(Abecasis et al. 2012)
NA20859	R	GIH	South Asia - West	(Abecasis et al. 2012)
NA20863	R	GIH	South Asia - West	(Abecasis et al. 2012)
NA21129	R	GIH	South Asia - West	(Abecasis et al. 2012)
NA21106	R	GIH	South Asia - West	(Abecasis et al. 2012)
NA21087	R	GIH	South Asia - West	(Abecasis et al. 2012)
NA20847	R	GIH	South Asia - West	(Abecasis et al. 2012)
NA20884	R	GIH	South Asia - West	(Abecasis et al. 2012)
NA21094	R	GIH	South Asia - West	(Abecasis et al. 2012)
NA21088	R	GIH	South Asia - West	(Abecasis et al. 2012)
AY713998	R5	Uttar Pradesh	South Asia - Central	(Palanichamy et al. 2004)
HG03685	R5	STU	South Asia - South	(Abecasis et al. 2012)
FJ004822	R5	Uttar Pradesh	South Asia - Central	(Chaubey et al. 2008)
FJ004806	R5	West Bengal	South Asia - East	(Chaubey et al. 2008)
HG03790	R5	ITU	South Asia - South	(Abecasis et al. 2012)
AY713984	R5	Uttar Pradesh	South Asia - Central	(Palanichamy et al. 2004)
AY713983	R5	Uttar Pradesh	South Asia - Central	(Palanichamy et al. 2004)
NA20868	R5	GIH	South Asia - West	(Abecasis et al. 2012)
NA20871	R5	GIH	South Asia - West	(Abecasis et al. 2012)
FJ004837	R5	Gujarat	South Asia - West	(Chaubey et al. 2008)
HG03762	R5	PJL	South Asia - West	(Abecasis et al. 2012)
AY713993	R5	Andhra Pradesh	South Asia - South	(Palanichamy et al. 2004)
HG03667	R5	PJL	South Asia - West	(Abecasis et al. 2012)
HG03729	R5	ITU	South Asia - South	(Abecasis et al. 2012)

Table S1. (*continued*)

Sample	Haplogroup	Origin (Population)	Region	Reference
KJ446146	R5	Pakistan: Sindhi	South Asia - West	HGDP - Zheng et al. (unpublished)
KJ446149	R5	Israel: Druze	Southwest Asia	HGDP - Zheng et al. (unpublished)
KJ446147	R5	Pakistan: Burusho	South Asia - West	HGDP - Zheng et al. (unpublished)
HG03973	R5	ITU	South Asia - South	(Abecasis et al. 2012)
NA21099	R5	GIH	South Asia - West	(Abecasis et al. 2012)
KJ446148	R5	Pakistan: Sindhi	South Asia - West	HGDP - Zheng et al. (unpublished)
AY713985	R5	Uttar Pradesh	South Asia - Central	(Palanichamy et al. 2004)
HG03629	R5	PJL	South Asia - West	(Abecasis et al. 2012)
AY713996	R5	Uttar Pradesh	South Asia - Central	(Palanichamy et al. 2004)
HG03812	R5	BEB	South Asia - East	(Abecasis et al. 2012)
NA20852	R5	GIH	South Asia - West	(Abecasis et al. 2012)
AY713991	R5	Uttar Pradesh	South Asia - Central	(Palanichamy et al. 2004)
AY713997	R5	Uttar Pradesh	South Asia - Central	(Palanichamy et al. 2004)
AY713989	R5	Uttar Pradesh	South Asia - Central	(Palanichamy et al. 2004)
AY714000	R5	Uttar Pradesh	South Asia - Central	(Palanichamy et al. 2004)
AY714002	R5	Uttar Pradesh	South Asia - Central	(Palanichamy et al. 2004)
JX094847	R5	unknown	unknown	Family Tree
FJ004829	R5	Orissa	South Asia - East	(Chaubey et al. 2008)
FJ004833	R5	Sri Lanka	South Asia - South	(Chaubey et al. 2008)
FJ004812	R5	Gujarat	South Asia - West	(Chaubey et al. 2008)
FJ004834	R5	Kerala	South Asia - South	(Chaubey et al. 2008)
FJ004828	R5	Kerala	South Asia - South	(Chaubey et al. 2008)
FJ004814	R5	Tamil Nadu	South Asia - South	(Chaubey et al. 2008)
FJ004815	R5	Tamil Nadu	South Asia - South	(Chaubey et al. 2008)
GU480010	R5	Madhya Pradesh	South Asia - Central	(Sharma et al. 2012)
HG03931	R5	BEB	South Asia - East	(Abecasis et al. 2012)
HG03836	R5	STU	South Asia - South	(Abecasis et al. 2012)
HG03760	R5	STU	South Asia - South	(Abecasis et al. 2012)
HG04035	R5	STU	South Asia - South	(Abecasis et al. 2012)
KC911551	R5	Iran: Persian	Southwest Asia	(Derenko et al. 2013)
HG03770	R5	ITU	South Asia - South	(Abecasis et al. 2012)
HM036560	R5	Great Himalayas: Ladakh tribe	South Asia - Central	Sharma et al. (unpublished)
HM036562	R5	Great Himalayas: Ladakh tribe	South Asia - Central	Sharma et al. (unpublished)
AY713994	R6	Uttar Pradesh	South Asia - Central	(Palanichamy et al. 2004)
AY714028	R6	Uttar Pradesh	South Asia - Central	(Palanichamy et al. 2004)
JF742197	R6	Nepal: Kathmandu	South Asia - Central	(Wang et al. 2012)
HG03821	R6	BEB	South Asia - East	(Abecasis et al. 2012)
HG04189	R6	BEB	South Asia - East	(Abecasis et al. 2012)

Table S1. (*continued*)

Sample	Haplogroup	Origin (Population)	Region	Reference
HG03689	R6	STU	South Asia - South	(Abecasis et al. 2012)
HG03789	R6	ITU	South Asia - South	(Abecasis et al. 2012)
JX289095	R6	Myanmar	Southeast Asia	(Summerer et al. 2014)
GU480008	R6	Madhya Pradesh	South Asia - Central	(Sharma et al. 2012)
AY714019	R6	Andhra Pradesh	South Asia - South	(Palanichamy et al. 2004)
HG03491	R6	PJL	South Asia - West	(Abecasis et al. 2012)
tha166	R6	Thailand	Southeast Asia	This study
JQ704804	R6	unknown	unknown	(Behar et al. 2012)
KC577359	R6	Mauritius	Africa	(Fregel et al. 2014)
HG03604	R6	BEB	South Asia - East	(Abecasis et al. 2012)
HG02600	R6	PJL	South Asia - West	(Abecasis et al. 2012)
GU480018	R6	Madhya Pradesh	South Asia - Central	(Sharma et al. 2012)
ML9	R6	Myanmar	Southeast Asia	This study
FJ004819	R6	Andhra Pradesh	South Asia - South	(Chaubey et al. 2008)
FJ004816	R6	Andhra Pradesh	South Asia - South	(Chaubey et al. 2008)
HG03920	R6	BEB	South Asia - East	(Abecasis et al. 2012)
HG03837	R6	STU	South Asia - South	(Abecasis et al. 2012)
HG03741	R7	STU	South Asia - South	(Abecasis et al. 2012)
HG03965	R7	ITU	South Asia - South	(Abecasis et al. 2012)
FJ770943	R7	Andhra Pradesh	South Asia - South	(Fornarino et al. 2009)
HG03850	R7	STU	South Asia - South	(Abecasis et al. 2012)
AY714030	R7	Andhra Pradesh	South Asia - South	(Palanichamy et al. 2004)
GU480017	R7	Madhya Pradesh	South Asia - Central	(Sharma et al. 2012)
FJ004835	R7	Andhra Pradesh	South Asia - South	(Chaubey et al. 2008)
FJ004805	R7	Jharkhand	South Asia - East	(Chaubey et al. 2008)
FJ004832	R7	Chhattisgarh	South Asia - Central	(Chaubey et al. 2008)
FJ770970	R7	Pakistan	South Asia - West	(Chaubey et al. 2008)
FJ004823	R7	Pakistan	South Asia - West	(Chaubey et al. 2008)
FJ004831	R7	Jharkhand	South Asia - East	(Chaubey et al. 2008)
FJ004813	R7	Jharkhand	South Asia - East	(Chaubey et al. 2008)
FJ004809	R7	Jharkhand	South Asia - East	(Chaubey et al. 2008)
FJ004804	R7	Jharkhand	South Asia - East	(Chaubey et al. 2008)
FJ004810	R7	Bihar	South Asia - East	(Chaubey et al. 2008)
HG04227	R7	STU	South Asia - South	(Abecasis et al. 2012)
FJ004817	R7	Andhra Pradesh	South Asia - South	(Chaubey et al. 2008)
AY714024	R7	Uttar Pradesh	South Asia - Central	(Palanichamy et al. 2004)
FJ004811	R7	Madhya Pradesh	South Asia - Central	(Chaubey et al. 2008)
FJ004830	R7	Orissa	South Asia - East	(Chaubey et al. 2008)
FJ004821	R7	Andhra Pradesh	South Asia - South	(Chaubey et al. 2008)
GU170816	R7	Tamil Nadu	South Asia - South	(Rani et al. 2010)
GU170821	R7	Tamil Nadu	South Asia - South	(Rani et al. 2010)
GU170819	R7	Tamil Nadu	South Asia - South	(Rani et al. 2010)

Table S1. (*continued*)

Sample	Haplogroup	Origin (Population)	Region	Reference
HG03947	R7	STU	South Asia - South	(Abecasis et al. 2012)
HG04029	R7	STU	South Asia - South	(Abecasis et al. 2012)
HG03851	R7	STU	South Asia - South	(Abecasis et al. 2012)
KK27	R7	Borneo, East Malaysia: Kota Kinabalu	Southeast Asia	This study
FJ467953	R8	Andhra Pradesh	South Asia - South	(Thangaraj et al. 2009)
JF742196	R8	Nepal: Kathmandu	South Asia - Central	(Wang et al. 2012)
JF742200	R8	Nepal: Kathmandu	South Asia - Central	(Wang et al. 2012)
FJ467943	R8	Jharkhand	South Asia - East	(Thangaraj et al. 2009)
KC911621	R8	Iran: Qashqai	Southwest Asia	(Derenko et al. 2013)
FJ467978	R8	Madhya Pradesh	South Asia - Central	(Thangaraj et al. 2009)
AY714012	R8	Uttar Pradesh	South Asia - Central	(Palanichamy et al. 2004)
FJ467951	R8	Orissa	South Asia - East	(Thangaraj et al. 2009)
FJ467979	R8	Andhra Pradesh	South Asia - South	(Thangaraj et al. 2009)
FJ467949	R8	Andhra Pradesh	South Asia - South	(Thangaraj et al. 2009)
FJ467944	R8	Madhya Pradesh	South Asia - Central	(Thangaraj et al. 2009)
FJ467955	R8	Andhra Pradesh	South Asia - South	(Thangaraj et al. 2009)
FJ467966	R8	Madhya Pradesh	South Asia - Central	(Thangaraj et al. 2009)
GU480002	R8	Madhya Pradesh	South Asia - Central	(Sharma et al. 2012)
FJ467942	R8	Gujarat	South Asia - West	(Thangaraj et al. 2009)
FJ467956	R8	Uttar Pradesh	South Asia - Central	(Thangaraj et al. 2009)
FJ467947	R8	Orissa	South Asia - East	(Thangaraj et al. 2009)
FJ467964	R8	Chhattisgarh	South Asia - Central	(Thangaraj et al. 2009)
FJ467965	R8	Chhattisgarh	South Asia - Central	(Thangaraj et al. 2009)
FJ004808	R8	Maharashtra	South Asia - South	(Chaubey et al. 2008)
FJ467952	R8	Jharkhand	South Asia - East	(Thangaraj et al. 2009)
FJ004818	R8	Andhra Pradesh	South Asia - South	(Chaubey et al. 2008)
FJ004820	R8	Andhra Pradesh	South Asia - South	(Chaubey et al. 2008)
FJ467993	R8	Gujarat	South Asia - West	(Thangaraj et al. 2009)
FJ467976	R8	Orissa	South Asia - East	(Thangaraj et al. 2009)
FJ467983	R8	Orissa	South Asia - East	(Thangaraj et al. 2009)
FJ467971	R8	Orissa	South Asia - East	(Thangaraj et al. 2009)
FJ467982	R8	Andhra Pradesh	South Asia - South	(Thangaraj et al. 2009)
FJ467975	R8	Orissa	South Asia - East	(Thangaraj et al. 2009)
FJ467974	R8	Orissa	South Asia - East	(Thangaraj et al. 2009)
FJ004838	R8	Andhra Pradesh	South Asia - South	(Chaubey et al. 2008)
AY714011	R8	Meghalaya	South Asia - East	(Palanichamy et al. 2004)
FJ467984	R8	Madhya Pradesh	South Asia - Central	(Thangaraj et al. 2009)
FJ467972	R8	Orissa	South Asia - East	(Thangaraj et al. 2009)
FJ467973	R8	Orissa	South Asia - East	(Thangaraj et al. 2009)
FJ467968	R8	Orissa	South Asia - East	(Thangaraj et al. 2009)
FJ467969	R8	Orissa	South Asia - East	(Thangaraj et al. 2009)

Table S1. (*continued*)

Sample	Haplogroup	Origin (Population)	Region	Reference
FJ467970	R8	Orissa	South Asia - East	(Thangaraj et al. 2009)
FJ467950	R8	Andhra Pradesh	South Asia - South	(Thangaraj et al. 2009)
NA21144	R8	GIH	South Asia - West	(Abecasis et al. 2012)
FJ467945	R8	Andhra Pradesh	South Asia - South	(Thangaraj et al. 2009)
FJ004836	R8	Andhra Pradesh	South Asia - South	(Chaubey et al. 2008)
FJ467980	R8	Andhra Pradesh	South Asia - South	(Thangaraj et al. 2009)
FJ467986	R8	Andhra Pradesh	South Asia - South	(Thangaraj et al. 2009)
FJ467985	R8	Andhra Pradesh	South Asia - South	(Thangaraj et al. 2009)
FJ467946	R8	Andhra Pradesh	South Asia - South	(Thangaraj et al. 2009)
FJ467967	R8	Andhra Pradesh	South Asia - South	(Thangaraj et al. 2009)
FJ467981	R8	Andhra Pradesh	South Asia - South	(Thangaraj et al. 2009)
FJ467941	R8	Andhra Pradesh	South Asia - South	(Thangaraj et al. 2009)
FJ467961	R8	Gujarat	South Asia - West	(Thangaraj et al. 2009)
FJ467962	R8	Gujarat	South Asia - West	(Thangaraj et al. 2009)
FJ467963	R8	Gujarat	South Asia - West	(Thangaraj et al. 2009)
FJ467960	R8	Gujarat	South Asia - West	(Thangaraj et al. 2009)
FJ467958	R8	Gujarat	South Asia - West	(Thangaraj et al. 2009)
FJ467959	R8	Gujarat	South Asia - West	(Thangaraj et al. 2009)
FJ467957	R8	Gujarat	South Asia - West	(Thangaraj et al. 2009)
FJ467948	R8	Tamil Nadu	South Asia - South	(Thangaraj et al. 2009)
HG03864	R8	ITU	South Asia - South	(Abecasis et al. 2012)
FJ467977	R8	Jharkhand	South Asia - East	(Thangaraj et al. 2009)
AY714009	R8	Uttar Pradesh	South Asia - Central	(Palanichamy et al. 2004)
FJ467954	R8	Andhra Pradesh	South Asia - South	(Thangaraj et al. 2009)
FJ467940	R8	Andhra Pradesh	South Asia - South	(Thangaraj et al. 2009)
HM156676	R8	Andhra Pradesh	South Asia - South	(Govindaraj et al. 2011)
HG03736	R8	STU	South Asia - South	(Abecasis et al. 2012)
FJ467989	R8	Orissa	South Asia - East	(Thangaraj et al. 2009)
FJ467991	R8	Orissa	South Asia - East	(Thangaraj et al. 2009)
FJ467992	R8	Orissa	South Asia - East	(Thangaraj et al. 2009)
FJ467990	R8	Orissa	South Asia - East	(Thangaraj et al. 2009)
FJ467987	R8	Orissa	South Asia - East	(Thangaraj et al. 2009)
FJ467988	R8	Orissa	South Asia - East	(Thangaraj et al. 2009)
AY714006	R30	Andhra Pradesh	South Asia - South	(Palanichamy et al. 2004)
HG03887	R30	STU	South Asia - South	(Abecasis et al. 2012)
HG04061	R30	ITU	South Asia - South	(Abecasis et al. 2012)
AY714001	R30	Andhra Pradesh	South Asia - South	(Palanichamy et al. 2004)
FJ770961	R30	Nepal	South Asia - Central	(Fornarino et al. 2009)
PAD90	R30	Indonesia: Padang	Southeast Asia	This study
FJ004824	R30	Sri Lanka	South Asia - South	(Chaubey et al. 2008)
HG03645	R30	STU	South Asia - South	(Abecasis et al. 2012)
HG03949	R30	STU	South Asia - South	(Abecasis et al. 2012)

Table S1. (*continued*)

Sample	Haplogroup	Origin (Population)	Region	Reference
HG03872	R30	ITU	South Asia - South	(Abecasis et al. 2012)
KC911324	R30	Iran: Persian	Southwest Asia	(Derenko et al. 2013)
KC911618	R30	Iran: Persian	Southwest Asia	(Derenko et al. 2013)
HG04018	R30	ITU	South Asia - South	(Abecasis et al. 2012)
GU170818	R30	Tamil Nadu	South Asia - South	(Rani et al. 2010)
NA21110	R30	GIH	South Asia - West	(Abecasis et al. 2012)
HG03937	R30	BEB	South Asia - East	(Abecasis et al. 2012)
HG03911	R30	BEB	South Asia - East	(Abecasis et al. 2012)
AY714032	R30	Uttar Pradesh	South Asia - Central	(Palanichamy et al. 2004)
HG01593	R30	PJL	South Asia - West	(Abecasis et al. 2012)
AY714047	R30	Uttar Pradesh	South Asia - Central	(Palanichamy et al. 2004)
FJ004827	R30	Punjab	South Asia - West	(Chaubey et al. 2008)
AY714050	R30	Uttar Pradesh	South Asia - Central	(Palanichamy et al. 2004)
HG02697	R30	PJL	South Asia - West	(Abecasis et al. 2012)
KF038316	R30	Gujarat: Surat	South Asia - West	Wilson (direct submission)
HG03714	R30	ITU	South Asia - South	(Abecasis et al. 2012)
HG03679	R30	STU	South Asia - South	(Abecasis et al. 2012)
HG03680	R30	STU	South Asia - South	(Abecasis et al. 2012)
NA21102	R30	GIH	South Asia - West	(Abecasis et al. 2012)
NA21105	R30	GIH	South Asia - West	(Abecasis et al. 2012)
NA20902	R30	GIH	South Asia - West	(Abecasis et al. 2012)
HG03788	R30	ITU	South Asia - South	(Abecasis et al. 2012)
HG03874	R30	ITU	South Asia - South	(Abecasis et al. 2012)
NA20856	R30	GIH	South Asia - West	(Abecasis et al. 2012)
NA21115	R30	GIH	South Asia - West	(Abecasis et al. 2012)
FJ004807	R30	Maharashtra	South Asia - South	(Chaubey et al. 2008)
GU170815	R30	Tamil Nadu	South Asia - South	(Rani et al. 2010)
AY714021	R31	Uttar Pradesh	South Asia - Central	(Palanichamy et al. 2004)
FJ004826	R31	Rajasthan	South Asia - West	(Chaubey et al. 2008)
FJ004825	R31	Rajasthan	South Asia - West	(Chaubey et al. 2008)
NA21137	R31	GIH	South Asia - West	(Abecasis et al. 2012)
NA20861	R31	GIH	South Asia - West	(Abecasis et al. 2012)
NA21089	R31	GIH	South Asia - West	(Abecasis et al. 2012)
JQ705959	R31	unknown	unknown	(Behar et al. 2012)
AY714046	R31	Andhra Pradesh	South Asia - South	(Palanichamy et al. 2004)
AY714048	R31	Andhra Pradesh	South Asia - South	(Palanichamy et al. 2004)
HG03755	R31	STU	South Asia - South	(Abecasis et al. 2012)
HG03738	R31	STU	South Asia - South	(Abecasis et al. 2012)
HG03750	R31	STU	South Asia - South	(Abecasis et al. 2012)
HG03854	R31	STU	South Asia - South	(Abecasis et al. 2012)
AY713992	U2	Uttar Pradesh	South Asia - Central	(Palanichamy et al. 2004)
HG03742	U2	ITU	South Asia - South	(Abecasis et al. 2012)

Table S1. (*continued*)

Sample	Haplogroup	Origin (Population)	Region	Reference
HG03985	U2	STU	South Asia - South	(Abecasis et al. 2012)
HG04002	U2	ITU	South Asia - South	(Abecasis et al. 2012)
AY713990	U2	Uttar Pradesh	South Asia - Central	(Palanichamy et al. 2004)
AY882379	U2	Pakistan	South Asia - West	(Achilli et al. 2005)
HG03829	U2	BEB	South Asia - East	(Abecasis et al. 2012)
NA20866	U2	GIH	South Asia - West	(Abecasis et al. 2012)
JX488759	U2	Eastern Indian	South Asia - East	Family Tree
HG03653	U2	PJL	South Asia - West	(Abecasis et al. 2012)
NA20875	U2	GIH	South Asia - West	(Abecasis et al. 2012)
NA20904	U2	GIH	South Asia - West	(Abecasis et al. 2012)
HG03960	U2	ITU	South Asia - South	(Abecasis et al. 2012)
AY714020	U2	Meghalaya	South Asia - East	(Palanichamy et al. 2004)
HG03861	U2	ITU	South Asia - South	(Abecasis et al. 2012)
WA8	U2	Myanmar	Southeast Asia	This study
HG04106	U2	STU	South Asia - South	(Abecasis et al. 2012)
HG03695	U2	STU	South Asia - South	(Abecasis et al. 2012)
HG03754	U2	STU	South Asia - South	(Abecasis et al. 2012)
HG04107	U2	STU	South Asia - South	(Abecasis et al. 2012)
NA21119	U2	GIH	South Asia - West	(Abecasis et al. 2012)
HG03767	U2	PJL	South Asia - West	(Abecasis et al. 2012)
AY714025	U2	Uttar Pradesh	South Asia - Central	(Palanichamy et al. 2004)
HG03694	U2	STU	South Asia - South	(Abecasis et al. 2012)
HG03823	U2	BEB	South Asia - East	(Abecasis et al. 2012)
HG03826	U2	BEB	South Asia - East	(Abecasis et al. 2012)
HG04038	U2	STU	South Asia - South	(Abecasis et al. 2012)
HG04212	U2	ITU	South Asia - South	(Abecasis et al. 2012)
NA21095	U2	GIH	South Asia - West	(Abecasis et al. 2012)
HM156680	U2	Andhra Pradesh: Mahabubnagar	South Asia - South	(Govindaraj et al. 2011)
HM156685	U2	Andhra Pradesh: Mahabubnagar	South Asia - South	(Govindaraj et al. 2011)
HQ153103	U2	Sri Lanka	South Asia - South	Illeperuma & Bamshad (unpublished)
AY714027	U2	Andhra Pradesh	South Asia - South	(Palanichamy et al. 2004)
HG04200	U2	ITU	South Asia - South	(Abecasis et al. 2012)
NA20887	U2	GIH	South Asia - West	(Abecasis et al. 2012)
AY882380	U2	Pakistan	South Asia - West	(Achilli et al. 2005)
HG03703	U2	PJL	South Asia - West	(Abecasis et al. 2012)
HG04222	U2	ITU	South Asia - South	(Abecasis et al. 2012)
HG02603	U2	PJL	South Asia - West	(Abecasis et al. 2012)
HG03603	U2	BEB	South Asia - East	(Abecasis et al. 2012)
HG03908	U2	BEB	South Asia - East	(Abecasis et al. 2012)
HG03802	U2	BEB	South Asia - East	(Abecasis et al. 2012)
HG04158	U2	BEB	South Asia - East	(Abecasis et al. 2012)

Table S1. (*continued*)

Sample	Haplogroup	Origin (Population)	Region	Reference
HG04214	U2	ITU	South Asia - South	(Abecasis et al. 2012)
AY714010	U2	West Bengal	South Asia - East	(Palanichamy et al. 2004)
KC911315	U2	Iran: Persian	Southwest Asia	(Derenko et al. 2013)
AY882381	U2	Pakistan	South Asia - West	(Achilli et al. 2005)
HG03619	U2	PJL	South Asia - West	(Abecasis et al. 2012)
NA21111	U2	GIH	South Asia - West	(Abecasis et al. 2012)
AY714005	U2	Andhra Pradesh	South Asia - South	(Palanichamy et al. 2004)
HG03817	U2	BEB	South Asia - East	(Abecasis et al. 2012)
HG03691	U2	STU	South Asia - South	(Abecasis et al. 2012)
HG04099	U2	STU	South Asia - South	(Abecasis et al. 2012)
HM036565	U2	Great Himalayas: Ladakh tribe	South Asia - Central	Sharma et al. (unpublished)
HM036556	U2	Great Himalayas: Ladakh tribe	South Asia - Central	Sharma et al. (unpublished)
KC911602	U2	Iran: Persian	Southwest Asia	(Derenko et al. 2013)
JQ706041	U2	Iran	Southwest Asia	(Behar et al. 2012)
KC911489	U2	Iran: Persian	Southwest Asia	(Derenko et al. 2013)
KC911405	U2	Iran: Bakhtiari	Southwest Asia	(Derenko et al. 2013)
KC911513	U2	Iran: Qashqai	Southwest Asia	(Derenko et al. 2013)
KC911479	U2	Iran: Persian	Southwest Asia	(Derenko et al. 2013)
AY714040	U2	Andhra Pradesh: Thogataveera	South Asia - South	(Palanichamy et al. 2004)
KC911567	U2	Azerbaijan: Azeri	Southwest Asia	(Derenko et al. 2013)
HM156683	U2	Andhra Pradesh: Mahabubnagar	South Asia - South	(Govindaraj et al. 2011)
HM156688	U2	Andhra Pradesh: Mahabubnagar	South Asia - South	(Govindaraj et al. 2011)

References:

Abecasis GR, Auton A, Brooks LD, et al (2012) An integrated map of genetic variation from 1,092 human genomes. Nature 491:56–65.

Achilli A, Rengo C, Battaglia V, et al (2005) Saami and Berbers--an unexpected mitochondrial DNA link. Am J Hum Genet 76:883–6.

Al-Zahery N, Pala M, Battaglia V, et al (2011) In search of the genetic footprints of Sumerians: a survey of Y-chromosome and mtDNA variation in the Marsh Arabs of Iraq. BMC Evol Biol 11:288.

Barik SS, Sahani R, Prasad BVR, et al (2008) Detailed mtDNA genotypes permit a reassessment of the settlement and population structure of the Andaman Islands. Am J Phys Anthropol 136:19–27.

Behar DM, Metspalu E, Kivisild T, et al (2008) Counting the founders: the matrilineal genetic ancestry of the Jewish Diaspora. PLoS One 3:e2062.

Behar DM, van Oven M, Rosset S, et al (2012) A “Copernican” reassessment of the human mitochondrial DNA tree from its root. Am J Hum Genet 90:675–84.

Chandrasekar A, Kumar S, Sreenath J, et al (2009) Updating phylogeny of mitochondrial DNA macrohaplogroup m in India: dispersal of modern human in South Asian corridor. PLoS One 4:e7447.

Chaubey G, Karmin M, Metspalu E, et al (2008) Phylogeography of mtDNA haplogroup R7 in the Indian peninsula. BMC Evol Biol 8:227.

Derenko M, Malyarchuk B, Bahmanimehr A, et al (2013) Complete Mitochondrial DNA Diversity in Iranians. PLoS One 8:e80673.

Eaaswarkhanth M, Haque I, Ravesh Z, et al (2010) Traces of sub-Saharan and Middle Eastern lineages in Indian Muslim populations. Eur J Hum Genet 18:354–63.

Fornarino S, Pala M, Battaglia V, et al (2009) Mitochondrial and Y-chromosome diversity of the Tharus (Nepal): a reservoir of genetic variation. BMC Evol Biol 9:154.

Fregel R, Seetah K, Betancor E, et al (2014) Multiple ethnic origins of mitochondrial DNA lineages for the population of Mauritius. PLoS One 9:e93294.

Gómez-Carballa A, Pardo-Seco J, Fachal L, et al (2013) Indian signatures in the westernmost edge of the European Romani diaspora: new insight from mitogenomes. PLoS One 8:e75397.

Govindaraj P, Khan NA, Gopalakrishna P, et al (2011) Mitochondrial dysfunction and genetic heterogeneity in chronic periodontitis. Mitochondrion 11:504–12.

Hartmann A, Thieme M, Nanduri LK, et al (2009) Validation of microarray-based resequencing of 93 worldwide mitochondrial genomes. Hum Mutat 30:115–22.

Ingman M, Gyllensten U (2003) Mitochondrial genome variation and evolutionary history of Australian and New Guinean aborigines. Genome Res 13:1600–6.

Ji F, Sharpley MS, Derbeneva O, et al (2012) Mitochondrial DNA variant associated with Leber hereditary optic neuropathy and high-altitude Tibetans. Proc Natl Acad Sci U S A 109:7391–6.

Kang L, Zheng H-X, Chen F, et al (2013) mtDNA lineage expansions in Sherpa population suggest adaptive evolution in Tibetan highlands. Mol Biol Evol 30:2579–87.

Kong Q-P, Sun C, Wang H-W, et al (2011) Large-scale mtDNA screening reveals a surprising matrilineal complexity in east Asia and its implications to the peopling of the region. Mol Biol Evol 28:513–22.

Kumar S, Padmanabham PBS V, Ravuri RR, et al (2008) The earliest settlers' antiquity and evolutionary history of Indian populations: evidence from M2 mtDNA lineage. BMC Evol Biol 8:230.

Kumar S, Ravuri RR, Koneru P, et al (2009) Reconstructing Indian-Australian phylogenetic link. BMC Evol Biol 9:173.

Li S, Besenbacher S, Li Y, et al (2014) Variation and association to diabetes in 2000 full mtDNA sequences mined from an exome study in a Danish population. Eur J Hum Genet 22:1040–5.

Loo J-H, Trejaut JA, Yen J-C, et al (2014) Mitochondrial DNA association study of type 2 diabetes with or without ischemic stroke in Taiwan. BMC Res Notes 7:223.

Maca-Meyer N, Gonzalez A, Larruga J, et al (2001) Major genomic mitochondrial lineages delineate early human expansions. BMC Genet 2:13.

Macaulay V, Hill C, Achilli A, et al (2005) Single, rapid coastal settlement of Asia revealed by analysis of complete mitochondrial genomes. Science 308:1034–6.

Palanichamy MG, Mitra B, Debnath M, et al (2014) Tamil merchant in ancient Mesopotamia. PLoS One 9:e109331.

Palanichamy MG, Sun C, Agrawal S, et al (2004) Phylogeny of mitochondrial DNA macrohaplogroup N in India, based on complete sequencing: implications for the peopling of South Asia. Am J Hum Genet 75:966–78.

Rajkumar R, Banerjee J, Gunturi HB, et al (2005) Phylogeny and antiquity of M macrohaplogroup inferred from complete mt DNA sequence of Indian specific lineages. BMC Evol Biol 5:26.

Rani DS, Dhandapany PS, Nallari P, et al (2010) Mitochondrial DNA haplogroup "R" is associated with Noonan

syndrome of south India. Mitochondrion 10:166–73.

Schönberg A, Theunert C, Li M, et al (2011) High-throughput sequencing of complete human mtDNA genomes from the Caucasus and West Asia: high diversity and demographic inferences. Eur J Hum Genet 19:988–94.

Sharma G, Tamang R, Chaudhary R, et al (2012) Genetic affinities of the central Indian tribal populations. PLoS One 7:e32546.

Summerer M, Horst J, Erhart G, et al (2014) Large-scale mitochondrial DNA analysis in Southeast Asia reveals evolutionary effects of cultural isolation in the multi-ethnic population of Myanmar. BMC Evol Biol 14:17.

Sun C, Kong Q-P, Palanichamy MG, et al (2006) The dazzling array of basal branches in the mtDNA macrohaplogroup M from India as inferred from complete genomes. Mol Biol Evol 23:683–90.

Thangaraj K, Chaubey G, Kivisild T, et al (2005) Reconstructing the origin of Andaman Islanders. Science 308:996.

Thangaraj K, Chaubey G, Singh VK, et al (2006) In situ origin of deep rooting lineages of mitochondrial Macrohaplogroup “M” in India. BMC Genomics 7:151.

Thangaraj K, Nandan A, Sharma V, et al (2009) Deep rooting in-situ expansion of mtDNA Haplogroup R8 in South Asia. PLoS One 4:e6545.

Wang H-W, Li Y-C, Sun F, et al (2012) Revisiting the role of the Himalayas in peopling Nepal: insights from mitochondrial genomes. J Hum Genet 57:228–34.

Wang H-W, Mitra B, Chaudhuri TK, et al (2011) Mitochondrial DNA evidence supports northeast Indian origin of the aboriginal Andamanese in the Late Paleolithic. J Genet Genomics 38:117–22.

Zhang X, Qi X, Yang Z, et al (2013) Analysis of mitochondrial genome diversity identifies new and ancient maternal lineages in Cambodian aborigines. Nat Commun 4:1–11.

Table S2. List of non-autochthonous complete modern mtDNA sequences used in our analyses.

Population codes: BEB – Bengali from Bangladesh; GIH – Gujarati Indian from Houston, Texas; ITU – Indian Telugu from the UK; PJL – Punjabi from Lahore, Pakistan; STU – Sri Lankan Tamil from the UK; TSI – Tuscans from Italy; KHV – Kinh from Ho Chi Minh City, Vietnam; CDX – Chinese Dai from Xishuangbanna, China; CEU – Utah Residents (CEPH) with Northern and Western Ancestry.

Sample	Haplogroup	Origin (Population)	Region	Reference
EU597524	H1	Czech Republic	Europe	(Hartmann et al. 2009)
EU747355	H1	Germany	Europe	Family Tree
JQ702008	H1	Germany	Europe	(Behar et al. 2012)
JQ702174	H1	unknown	unknown	(Behar et al. 2012)
JQ702182	H1	unknown	unknown	(Behar et al. 2012)
JQ704245	H1	Ireland	Europe	(Behar et al. 2012)
JQ704536	H1	unknown	unknown	(Behar et al. 2012)
JX885690	H1	England	Europe	Family Tree
AY713977	H1	India: Andhra Pradesh	South Asia - South	(Palanichamy et al. 2004)
AY738961	H2	Iraq	Southwest Asia	(Achilli et al. 2004)
HQ659667	H2	Germany	Europe	Family Tree
JQ702621	H2	unknown	unknown	(Behar et al. 2012)
JQ704274	H2	unknown	unknown	(Behar et al. 2012)
JQ704530	H2	unknown	unknown	(Behar et al. 2012)
NA07051	H2	CEU	Europe	(Sudmant et al. 2015)
NA20585	H2	TSI	Europe	(Sudmant et al. 2015)
NA20802	H2	TSI	Europe	(Sudmant et al. 2015)
EU597574	H2	Pakistan: Brahui	South Asia - West	(Hartmann et al. 2009)
HG01586	H2	PJL	South Asia - West	(Sudmant et al. 2015)
HG04039	H2	STU	South Asia - South	(Sudmant et al. 2015)
KJ446362	H2	Pakistan: Pathan	South Asia - West	HGDP - Zheng, et al. (unpublished)
KP763834	H2	India	South Asia	(Palanichamy et al. 2015)
NA20872	H2	GIH	South Asia - West	(Sudmant et al. 2015)
KJ856744	H2	Altai	Central Asia/Siberia	(Derenko et al. 2014)
KJ856726	H2	Altai	Central Asia/Siberia	(Derenko et al. 2014)
KJ856720	H2	Khamnigan	Central Asia/Siberia	(Derenko et al. 2014)
KF161829	H2	Denmark	Europe	(Li et al. 2014)
KJ446360	H2	Russia	Europe	HGDP - Zheng, et al. (unpublished)
KC990675	H2	Possibly India	South Asia	Ramanan et al. (unpublished)
JQ705227	H2	unknown	unknown	(Behar et al. 2012)
JQ702943	H2	Greece	Europe	(Behar et al. 2012)
EU600329	H6	Druze	Southwest Asia	(Shlush et al. 2008)
FJ348203	H6	Hutterite	Europe	HGDP - Zheng, et al. (unpublished)
JQ702285	H6	unknown	unknown	(Behar et al. 2012)
NA20850	H6	GIH	South Asia - West	(Sudmant et al. 2015)
HM852826	H7	Iran	Southwest Asia	(Schönberg et al. 2011)
JQ702601	H7	Germany	Europe	(Behar et al. 2012)
JQ702990	H7	unknown	unknown	(Behar et al. 2012)
JQ704890	H7	unknown	unknown	(Behar et al. 2012)
KC911403	H7	Iran: Persian	Southwest Asia	(Derenko et al. 2013)

Table S2. (*continued*)

Sample	Haplogroup	Origin (Population)	Region	Reference
NA20870	H7	GIH	South Asia - West	(Sudmant et al. 2015)
AY713978	H9	India: Uttar Pradesh	South Asia - Central	(Palanichamy et al. 2004)
AY738969	H9	Italy	Europe	(Achilli et al. 2004)
JQ705022	H9	Germany	Europe	(Behar et al. 2012)
AY339409	H13	Finland	Europe	(Finnilä et al. 2001)
AY495107	H13	Europe	Europe	(Coble et al. 2004)
AY495149	H13	Europe	Europe	(Coble et al. 2004)
EF556150	H13	Israel: Jew	Southwest Asia	(Behar et al. 2008a)
EU597515	H13	Palestine	Southwest Asia	(Hartmann et al. 2009)
AY713980	H13	India: Andhra Pradesh	South Asia - South	(Palanichamy et al. 2004)
AY713982	H13	India: Uttar Pradesh	South Asia - Central	(Palanichamy et al. 2004)
JQ702112	H13	England	Europe	(Behar et al. 2012)
JQ703858	H13	Ireland	Europe	(Behar et al. 2012)
KC911327	H13	Iran: Persian	Southwest Asia	(Derenko et al. 2013)
KC911369	H13	Iran: Qashqai	Southwest Asia	(Derenko et al. 2013)
KC911443	H13	Iran: Indian	Southwest Asia	(Derenko et al. 2013)
KC911454	H13	Russia: Saratov Region	Europe	(Derenko et al. 2013)
HG01589	H13	PJL	South Asia - West	(Sudmant et al. 2015)
HG02651	H13	PJL	South Asia - West	(Sudmant et al. 2015)
HG03692	H13	STU	South Asia - South	(Sudmant et al. 2015)
HG03844	H13	STU	South Asia - South	(Sudmant et al. 2015)
HG03849	H13	STU	South Asia - South	(Sudmant et al. 2015)
HG03995	H13	STU	South Asia - South	(Sudmant et al. 2015)
KJ446338	H13	Pakistan: Balochi	South Asia - West	HGDP - Zheng, et al. (unpublished)
KJ446339	H13	Pakistan: Balochi	South Asia - West	HGDP - Zheng, et al. (unpublished)
KJ446340	H13	Pakistan: Brahui	South Asia - West	HGDP - Zheng, et al. (unpublished)
KJ446341	H13	Pakistan: Balochi	South Asia - West	HGDP - Zheng, et al. (unpublished)
AY713995	H15	India: Uttar Pradesh	South Asia - Central	(Palanichamy et al. 2004)
AY495146	H15	Europe	Europe	(Coble et al. 2004)
HM852832	H15	Iran	Southwest Asia	(Schönberg et al. 2011)
JQ704364	H15	unknown	unknown	(Behar et al. 2012)
KC911289	H15	Iran: Azeri	Southwest Asia	(Derenko et al. 2013)
KC911292	H15	Iran: Persian	Southwest Asia	(Derenko et al. 2013)
EU600335	H29	Druze	Southwest Asia	(Shlush et al. 2008)
EU600343	H29	Druze	Southwest Asia	(Shlush et al. 2008)
JQ704540	H29	Italy	Europe	(Behar et al. 2012)
KC911535	H29	Iran: Azeri	Southwest Asia	(Derenko et al. 2013)
NA20896	H29	GIH	South Asia - West	(Sudmant et al. 2015)
NA21098	H29	GIH	South Asia - West	(Sudmant et al. 2015)
AY713981	HV	India: Andhra Pradesh	South Asia - South	(Palanichamy et al. 2004)
EF222251	HV	Poland	Europe	(Malyarchuk et al. 2008b)

Table S2. (*continued*)

Sample	Haplogroup	Origin (Population)	Region	Reference
EU545435	HV	Russia	Europe	(Malyarchuk et al. 2008b)
HM852792	HV	Azeri	Southwest Asia	(Schönberg et al. 2011)
HM852816	HV	Iran	Southwest Asia	(Schönberg et al. 2011)
HQ287727	HV	Armenia	Southwest Asia	Family Tree
HQ436102	HV	Armenia	Southwest Asia	Family Tree
JF313325	HV	Sweden	Europe	Family Tree
KC765916	HV	Bulgaria	Europe	Family Tree
KC911391	HV	Iran: Persian	Southwest Asia	(Derenko et al. 2013)
KC911408	HV	Iran: Persian	Southwest Asia	(Derenko et al. 2013)
KC911439	HV	Iran: Persian	Southwest Asia	(Derenko et al. 2013)
KC911472	HV	Iran: Persian	Southwest Asia	(Derenko et al. 2013)
HG02491	HV	PJL	South Asia - West	(Sudmant et al. 2015)
HG02661	HV	PJL	South Asia - West	(Sudmant et al. 2015)
HG03235	HV	PJL	South Asia - West	(Sudmant et al. 2015)
HM776579	HV	India: Tamil Nadu	South Asia - South	Family Tree
DQ523620	HV0	Sardinia	Europe	(Fraumene et al. 2006)
JQ705544	HV0	unknown	unknown	(Behar et al. 2012)
AY713986	HV2	India: Uttar Pradesh	South Asia - Central	(Palanichamy et al. 2004)
HM852806	HV2	Azeri	Southwest Asia	(Schönberg et al. 2011)
KC911372	HV2	Iran: Persian	Southwest Asia	(Derenko et al. 2013)
KC911452	HV2	Iran: Qashqai	Southwest Asia	(Derenko et al. 2013)
NA20867	HV2	GIH	South Asia - West	(Sudmant et al. 2015)
AY713976	HV12	India: Andhra Pradesh	South Asia - South	(Palanichamy et al. 2004)
AY713987	HV12	India: Andhra Pradesh	South Asia - South	(Palanichamy et al. 2004)
HM852785	HV12	Armenia	Southwest Asia	(Schönberg et al. 2011)
KC911301	HV12	Iran: Persian	Southwest Asia	(Derenko et al. 2013)
KC911424	HV12	Iran: Qashqai	Southwest Asia	(Derenko et al. 2013)
KC911482	HV12	Iran: Mazandarani	Southwest Asia	(Derenko et al. 2013)
HG03009	HV12	BEB	South Asia - East	(Sudmant et al. 2015)
NA21112	HV12	GIH	South Asia - West	(Sudmant et al. 2015)
HM852828	HV13	Iran	Southwest Asia	(Schönberg et al. 2011)
JF700125	HV13	Armenia	Southwest Asia	Family Tree
KC911298	HV13	Iran: Persian	Southwest Asia	(Derenko et al. 2013)
HG02652	HV13	PJL	South Asia - West	(Sudmant et al. 2015)
AY713988	HV14	India: Andhra Pradesh	South Asia - South	(Palanichamy et al. 2004)
KC911456	HV14	Iran: Persian	Southwest Asia	(Derenko et al. 2013)
NA18743	HV14	Han Chinese	East Asia	(Sudmant et al. 2015)
HG03873	HV14	ITU	South Asia - South	(Sudmant et al. 2015)
HG03890	HV14	STU	South Asia - South	(Sudmant et al. 2015)
HG03895	HV14	STU	South Asia - South	(Sudmant et al. 2015)
HG03900	HV14	STU	South Asia - South	(Sudmant et al. 2015)
HG03998	HV14	STU	South Asia - South	(Sudmant et al. 2015)
HG03999	HV14	STU	South Asia - South	(Sudmant et al. 2015)
HG04003	HV14	STU	South Asia - South	(Sudmant et al. 2015)

Table S2. (*continued*)

Sample	Haplogroup	Origin (Population)	Region	Reference
HG04225	HV14	ITU	South Asia - South	(Sudmant et al. 2015)
KJ446056	I	Palestine	Southwest Asia	HGDP - Zheng, et al. (unpublished)
KJ446060	I	Palestine	Southwest Asia	HGDP - Zheng, et al. (unpublished)
AY714041	I1	India: Uttar Pradesh	South Asia - Central	(Palanichamy et al. 2004)
AY195769	I1	unknown	unknown	(Mishmar et al. 2003)
EF556153	I1	Tunisia: Jew	Africa	(Behar et al. 2008a)
FJ234984	I1	Armenia	Southwest Asia	Family Tree
JQ245776	I1	Turkey: Kurd	Southwest Asia	(Fernandes et al. 2012)
JQ705376	I1	unknown	unknown	(Behar et al. 2012)
JQ705840	I1	England	Europe	(Behar et al. 2012)
HG03727	I1	ITU	South Asia - South	(Sudmant et al. 2015)
KJ446055	I1	Pakistan: Burusho	South Asia - West	HGDP - Zheng, et al. (unpublished)
JQ245724	I5	Russia: North Ossetia	Southwest Asia	(Fernandes et al. 2012)
KJ446057	I5	Israel: Bedouin	Southwest Asia	HGDP - Zheng, et al. (unpublished)
AY714033	J1	India: Andhra Pradesh	South Asia - South	(Palanichamy et al. 2004)
AY714034	J1	India: Andhra Pradesh	South Asia - South	(Palanichamy et al. 2004)
AY714035	J1	India: Uttar Pradesh	South Asia - Central	(Palanichamy et al. 2004)
AY495202	J1	Europe	Europe	(Coble et al. 2004)
AY495216	J1	Europe	Europe	(Coble et al. 2004)
AY495234	J1	Europe	Europe	(Coble et al. 2004)
AY495235	J1	Europe	Europe	(Coble et al. 2004)
EF556155	J1	Iran: Jew	Southwest Asia	(Behar et al. 2008a)
EF583177	J1	Slovakia	Europe	(Malyarchuk et al. 2008a)
FJ348177	J1	Hutterite	Europe	HGDP - Zheng, et al. (unpublished)
FJ502349	J1	unknown	unknown	Family Tree
HM852779	J1	Armenia	Southwest Asia	(Schönberg et al. 2011)
HM852784	J1	Armenia	Southwest Asia	(Schönberg et al. 2011)
HM852838	J1	Iran	Southwest Asia	(Schönberg et al. 2011)
JF929909	J1	Armenia	Southwest Asia	Family Tree
JN663354	J1	Armenia	Southwest Asia	Family Tree
JQ703029	J1	unknown	unknown	(Behar et al. 2012)
JQ703802	J1	unknown	unknown	(Behar et al. 2012)
JQ703825	J1	unknown	unknown	(Behar et al. 2012)
JQ705141	J1	unknown	unknown	(Behar et al. 2012)
JQ705164	J1	Scotland	Europe	(Behar et al. 2012)
JQ797765	J1	Kuwait	Southwest Asia	(Pala et al. 2012)
JQ797770	J1	Iran	Southwest Asia	(Pala et al. 2012)
JQ797775	J1	Greece	Europe	(Pala et al. 2012)
KC911407	J1	Iran: Armenian	Southwest Asia	(Derenko et al. 2013)
KC911461	J1	Iran: Persian	Southwest Asia	(Derenko et al. 2013)

Table S2. (*continued*)

Sample	Haplogroup	Origin (Population)	Region	Reference
KC911496	J1	Iran: Gilak	Southwest Asia	(Derenko et al. 2013)
KJ445884	J1	Israel: Druze	Southwest Asia	HGDP - Zheng, et al. (unpublished)
KJ445886	J1	Palestine	Southwest Asia	HGDP - Zheng, et al. (unpublished)
KJ445907	J1	Israel: Bedouin	Southwest Asia	HGDP - Zheng, et al. (unpublished)
HG03744	J1	STU	South Asia - South	(Sudmant et al. 2015)
HG03833	J1	BEB	South Asia - East	(Sudmant et al. 2015)
HG03868	J1	ITU	South Asia - South	(Sudmant et al. 2015)
HG03902	J1	BEB	South Asia - East	(Sudmant et al. 2015)
KJ445885	J1	Pakistan: Pathan	South Asia - West	HGDP - Zheng, et al. (unpublished)
KJ445899	J1	Pakistan: Sindhi	South Asia - West	HGDP - Zheng, et al. (unpublished)
KJ445900	J1	Pakistan: Burusho	South Asia - West	HGDP - Zheng, et al. (unpublished)
KJ445901	J1	Pakistan: Burusho	South Asia - West	HGDP - Zheng, et al. (unpublished)
KJ445902	J1	Pakistan: Pathan	South Asia - West	HGDP - Zheng, et al. (unpublished)
KJ445904	J1	Pakistan: Brahui	South Asia - West	HGDP - Zheng, et al. (unpublished)
KJ445905	J1	Pakistan: Balochi	South Asia - West	HGDP - Zheng, et al. (unpublished)
KJ445906	J1	Pakistan: Balochi	South Asia - West	HGDP - Zheng, et al. (unpublished)
NA21126	J1	GIH	South Asia - West	(Sudmant et al. 2015)
JQ704041	J2	Ireland	Europe	(Behar et al. 2012)
HM852886	K	Georgia	Southwest Asia	(Schönberg et al. 2011)
AY714044	K1	India: Uttar Pradesh	South Asia - Central	(Palanichamy et al. 2004)
AF382005	K1	Spain	Europe	(Maca-Meyer et al. 2001)
EU259093	K1	unknown	unknown	Family Tree
HM852762	K1	Armenia	Southwest Asia	(Schönberg et al. 2011)
HQ586011	K1	USA	America	Family Tree
JN048471	K1	Armenia	Southwest Asia	Family Tree
JQ702086	K1	unknown	unknown	(Behar et al. 2012)
JQ702624	K1	unknown	unknown	(Behar et al. 2012)
JQ703522	K1	Syria	Southwest Asia	(Behar et al. 2012)
JQ706042	K1	Iran	Southwest Asia	(Behar et al. 2012)
JX273286	K1	Russia	Europe	(Costa et al. 2013)
KC911418	K1	Iran: Azeri	Southwest Asia	(Derenko et al. 2013)
KC911440	K1	Iran: Persian	Southwest Asia	(Derenko et al. 2013)
HG04014	K1	ITU	South Asia - South	(Sudmant et al. 2015)
AY714017	K2	India: Andhra Pradesh	South Asia - South	(Palanichamy et al. 2004)
EU597528	K2	Pakistan: Pathan	South Asia - West	(Hartmann et al. 2009)
AY495241	K2	Europe	Europe	(Coble et al. 2004)
AY495247	K2	Europe	Europe	(Coble et al. 2004)

Table S2. (*continued*)

Sample	Haplogroup	Origin (Population)	Region	Reference
DQ301796	K2	Yemen: Jew	Southwest Asia	(Behar et al. 2006)
JQ703018	K2	Ireland	Europe	(Behar et al. 2012)
JX021502	K2	Germany	Europe	Family Tree
JX273250	K2	Turkey	Southwest Asia	(Costa et al. 2013)
KC911393	K2	Iran: Persian	Southwest Asia	(Derenko et al. 2013)
KJ445985	K2	Pakistan: Sindhi	South Asia - West	HGDP - Zheng, et al. (unpublished)
NA20910	K2	GIH	South Asia - West	(Sudmant et al. 2015)
DQ341069	L3	Ethiopia	Africa	(Torroni et al. 2006)
EU092923	L3	Yemen	Southwest Asia	(Behar et al. 2008b)
JN655780	L3	Ethiopia	Africa	(Soares et al. 2012)
JN655785	L3	Ethiopia	Africa	(Soares et al. 2012)
NA21118	L3	GIH	South Asia - West	(Sudmant et al. 2015)
NA21142	L3	GIH	South Asia - West	(Sudmant et al. 2015)
AY714008	N1	India: Andhra Pradesh	South Asia - South	(Palanichamy et al. 2004)
JF298212	N1	Armenia	Southwest Asia	Family Tree
JQ245734	N1	United Arab Emirates: Dubai	Southwest Asia	(Fernandes et al. 2012)
JQ245766	N1	Turkey	Southwest Asia	(Fernandes et al. 2012)
JQ245777	N1	Yemen	Southwest Asia	(Fernandes et al. 2012)
KC911323	N1	Iran: Azeri	Southwest Asia	(Derenko et al. 2013)
KJ446050	N1	Israel: Bedouin	Southwest Asia	HGDP - Zheng, et al. (unpublished)
HG03698	N1	STU	South Asia - South	(Sudmant et al. 2015)
HG04238	N1	ITU	South Asia - South	(Sudmant et al. 2015)
KJ446052	N1	Pakistan: Balochi	South Asia - West	HGDP - Zheng, et al. (unpublished)
KJ446053	N1	Pakistan: Burusho	South Asia - West	HGDP - Zheng, et al. (unpublished)
KJ446070	N1	Pakistan: Balochi	South Asia - West	HGDP - Zheng, et al. (unpublished)
KP763833	N1	India	South Asia	(Palanichamy et al. 2015)
KP763844	N1	India	South Asia	(Palanichamy et al. 2015)
KC911431	N2	Iran: Persian	Southwest Asia	(Derenko et al. 2013)
HG04047	N5	STU	South Asia - South	(Sudmant et al. 2015)
GQ301867	N21	Vietnam	Southeast Asia	(Peng et al. 2010)
GQ301885	N21	Thailand	Southeast Asia	(Peng et al. 2010)
GQ301887	N21	China	East Asia	(Peng et al. 2010)
HG03943	N21	STU	South Asia - South	(Sudmant et al. 2015)
AY713999	R0	India: Uttar Pradesh	South Asia - Central	(Palanichamy et al. 2004)
EU597493	R0	Pakistan: Kalash	South Asia - West	(Hartmann et al. 2009)
HG02657	R0	PJL	South Asia - West	(Sudmant et al. 2015)
HG02072	R0	KHV	Southeast Asia	(Sudmant et al. 2015)
HG02399	R0	CDX	East Asia	(Sudmant et al. 2015)
HM185249	R0	Yemen	Southwest Asia	(Cerný et al. 2011)
HM852825	R0	Iran	Southwest Asia	(Schönberg et al. 2011)

Table S2. (*continued*)

Sample	Haplogroup	Origin (Population)	Region	Reference
KC911373	R0	Iran: Persian	Southwest Asia	(Derenko et al. 2013)
KJ446215	R0	Israel: Bedouin	Southwest Asia	HGDP - Zheng, et al. (unpublished)
KJ446216	R0	Palestine	Southwest Asia	HGDP - Zheng, et al. (unpublished)
KJ446217	R0	Palestine	Southwest Asia	HGDP - Zheng, et al. (unpublished)
KJ446218	R0	Palestine	Southwest Asia	HGDP - Zheng, et al. (unpublished)
KJ446219	R0	Palestine	Southwest Asia	HGDP - Zheng, et al. (unpublished)
KJ446220	R0	Palestine	Southwest Asia	HGDP - Zheng, et al. (unpublished)
KJ446221	R0	Israel: Bedouin	Southwest Asia	HGDP - Zheng, et al. (unpublished)
KJ446222	R0	Israel: Bedouin	Southwest Asia	HGDP - Zheng, et al. (unpublished)
KJ446223	R0	Israel: Druze	Southwest Asia	HGDP - Zheng, et al. (unpublished)
KJ446225	R0	Palestine	Southwest Asia	HGDP - Zheng, et al. (unpublished)
KJ446226	R0	Israel: Bedouin	Southwest Asia	HGDP - Zheng, et al. (unpublished)
KJ446227	R0	Israel: Bedouin	Southwest Asia	HGDP - Zheng, et al. (unpublished)
KJ446228	R0	Israel: Bedouin	Southwest Asia	HGDP - Zheng, et al. (unpublished)
KJ446206	R0	Pakistan: Kalash	South Asia - West	HGDP - Zheng, et al. (unpublished)
KJ446207	R0	Pakistan: Kalash	South Asia - West	HGDP - Zheng, et al. (unpublished)
KJ446208	R0	Pakistan: Kalash	South Asia - West	HGDP - Zheng, et al. (unpublished)
KJ446209	R0	Pakistan: Kalash	South Asia - West	HGDP - Zheng, et al. (unpublished)
KJ446211	R0	Pakistan: Kalash	South Asia - West	HGDP - Zheng, et al. (unpublished)
KJ446212	R0	Pakistan: Kalash	South Asia - West	HGDP - Zheng, et al. (unpublished)
KJ446213	R0	Pakistan: Kalash	South Asia - West	HGDP - Zheng, et al. (unpublished)
KJ446214	R0	Pakistan: Kalash	South Asia - West	HGDP - Zheng, et al. (unpublished)
KJ446224	R0	Pakistan: Pathan	South Asia - West	HGDP - Zheng, et al. (unpublished)
AY714045	R1	India: Uttar Pradesh	South Asia - Central	(Palanichamy et al. 2004)
AF381997	R1	Jordan	Southwest Asia	(Maca-Meyer et al. 2001)
EU545437	R1	Russia	Europe	(Malyarchuk et al. 2008b)
EU545445	R1	Russia	Europe	(Malyarchuk et al. 2008b)
HM852786	R1	Azeri	Southwest Asia	(Schönberg et al. 2011)
HQ602771	R1	Armenia	Southwest Asia	Family Tree
JQ705295	R1	Sweden	Europe	(Behar et al. 2012)

Table S2. (*continued*)

Sample	Haplogroup	Origin (Population)	Region	Reference
JQ705561	R1	England	Europe	(Behar et al. 2012)
AY714007	R2	India: Uttar Pradesh	South Asia - Central	(Palanichamy et al. 2004)
EU597551	R2	Pakistan: Brahui	South Asia - West	(Hartmann et al. 2009)
EF556167	R2	Yemen: Jew	Southwest Asia	(Behar et al. 2008a)
HM030516	R2	Mongolian	East Asia	(Kong et al. 2011)
HM852870	R2	Turk	Southwest Asia	(Schönberg et al. 2011)
JN581649	R2	Italy	Europe	(Bertolin et al. 2011)
JX155264	R2	Oman	Southwest Asia	(Al-Abri et al. 2012)
JX155265	R2	Oman	Southwest Asia	(Al-Abri et al. 2012)
JX155266	R2	Oman	Southwest Asia	(Al-Abri et al. 2012)
JX155267	R2	Oman	Southwest Asia	(Al-Abri et al. 2012)
JX155268	R2	Oman	Southwest Asia	(Al-Abri et al. 2012)
JX155269	R2	Oman	Southwest Asia	(Al-Abri et al. 2012)
JX155270	R2	Oman	Southwest Asia	(Al-Abri et al. 2012)
JX155271	R2	Oman	Southwest Asia	(Al-Abri et al. 2012)
JX155272	R2	Oman	Southwest Asia	(Al-Abri et al. 2012)
KC911280	R2	Iran: Persian	Southwest Asia	(Derenko et al. 2013)
KC911319	R2	Iran: Qashqai	Southwest Asia	(Derenko et al. 2013)
KC911337	R2	Iran: Persian	Southwest Asia	(Derenko et al. 2013)
KC911374	R2	Iran: Persian	Southwest Asia	(Derenko et al. 2013)
KC911379	R2	Iran: Qashqai	Southwest Asia	(Derenko et al. 2013)
KC911485	R2	Iran: Persian	Southwest Asia	(Derenko et al. 2013)
KC911495	R2	Iran: Persian	Southwest Asia	(Derenko et al. 2013)
KC911541	R2	Iran: Qashqai	Southwest Asia	(Derenko et al. 2013)
KC911627	R2	Iran: Persian	Southwest Asia	(Derenko et al. 2013)
KJ446063	R2	Pakistan: Makrani	South Asia - West	HGDP - Zheng, et al. (unpublished)
KJ446065	R2	Pakistan: Makrani	South Asia - West	HGDP - Zheng, et al. (unpublished)
KJ446066	R2	Pakistan: Balochi	South Asia - West	HGDP - Zheng, et al. (unpublished)
KJ446067	R2	Pakistan: Balochi	South Asia - West	HGDP - Zheng, et al. (unpublished)
KJ446069	R2	Pakistan: Balochi	South Asia - West	HGDP - Zheng, et al. (unpublished)
NA20877	R2	GIH	South Asia - West	(Sudmant et al. 2015)
NA20892	R2	GIH	South Asia - West	(Sudmant et al. 2015)
NA20899	R2	GIH	South Asia - West	(Sudmant et al. 2015)
NA21086	R2	GIH	South Asia - West	(Sudmant et al. 2015)
NA21091	R2	GIH	South Asia - West	(Sudmant et al. 2015)
NA21093	R2	GIH	South Asia - West	(Sudmant et al. 2015)
NA21117	R2	GIH	South Asia - West	(Sudmant et al. 2015)
NA21123	R2	GIH	South Asia - West	(Sudmant et al. 2015)
NA21141	R2	GIH	South Asia - West	(Sudmant et al. 2015)
KC911379	R2'JT	Iran: Qashqai	Southwest Asia	(Derenko et al. 2013)

Table S2. (*continued*)

Sample	Haplogroup	Origin (Population)	Region	Reference
AY714015	T1	India: Andhra Pradesh	South Asia - South	(Palanichamy et al. 2004)
AY714036	T1	India: Uttar Pradesh	South Asia - Central	(Palanichamy et al. 2004)
AY495288	T1	Europe	Europe	(Coble et al. 2004)
AY495289	T1	Europe	Europe	(Coble et al. 2004)
AY495290	T1	Europe	Europe	(Coble et al. 2004)
AY495293	T1	Europe	Europe	(Coble et al. 2004)
AY495295	T1	Europe	Europe	(Coble et al. 2004)
AY495296	T1	Europe	Europe	(Coble et al. 2004)
AY495297	T1	Europe	Europe	(Coble et al. 2004)
DQ358975	T1	unknown	unknown	(Detjen et al. 2007)
FJ348197	T1	Hutterite	Europe	HGDP - Zheng, et al. (unpublished)
HM184912	T1	Czech Republic	Europe	Family Tree
HM852798	T1	Azeri	Southwest Asia	(Schönberg et al. 2011)
JF831941	T1	unknown	unknown	Family Tree
JQ702340	T1	unknown	unknown	(Behar et al. 2012)
JQ702556	T1	England	Europe	(Behar et al. 2012)
JQ702716	T1	Ireland	Europe	(Behar et al. 2012)
JQ705353	T1	unknown	unknown	(Behar et al. 2012)
JQ705463	T1	unknown	unknown	(Behar et al. 2012)
KC911320	T1	Iran: Persian	Southwest Asia	(Derenko et al. 2013)
KC911343	T1	Iran: Persian	Southwest Asia	(Derenko et al. 2013)
KC911473	T1	Iran: Qashqai	Southwest Asia	(Derenko et al. 2013)
KJ445831	T1	Palestine	Southwest Asia	HGDP - Zheng, et al. (unpublished)
HG04093	T1	ITU	South Asia - South	(Sudmant et al. 2015)
KJ445834	T1	Pakistan: Brahui	South Asia - West	HGDP - Zheng, et al. (unpublished)
KJ445835	T1	Pakistan: Brahui	South Asia - West	HGDP - Zheng, et al. (unpublished)
KJ445839	T1	Pakistan: Pathan	South Asia - West	HGDP - Zheng, et al. (unpublished)
NA20890	T1	GIH	South Asia - West	(Sudmant et al. 2015)
AY714016	T2	India: Uttar Pradesh	South Asia - Central	(Palanichamy et al. 2004)
AY714022	T2	India: Andhra Pradesh	South Asia - South	(Palanichamy et al. 2004)
AY714029	T2	India: Uttar Pradesh	South Asia - Central	(Palanichamy et al. 2004)
AY714037	T2	India: Uttar Pradesh	South Asia - Central	(Palanichamy et al. 2004)
HG02604	T2	PJL	South Asia - West	(Sudmant et al. 2015)
HG02649	T2	PJL	South Asia - West	(Sudmant et al. 2015)
HG03625	T2	PJL	South Asia - West	(Sudmant et al. 2015)
HG03784	T2	ITU	South Asia - South	(Sudmant et al. 2015)
HG04023	T2	ITU	South Asia - South	(Sudmant et al. 2015)
AY495272	T2	Europe	Europe	(Coble et al. 2004)
AY495298	T2	Europe	Europe	(Coble et al. 2004)
AY495299	T2	Europe	Europe	(Coble et al. 2004)

Table S2. (*continued*)

Sample	Haplogroup	Origin (Population)	Region	Reference
AY495301	T2	Europe	Europe	(Coble et al. 2004)
AY495304	T2	Europe	Europe	(Coble et al. 2004)
HG04025	T2	ITU	South Asia - South	(Sudmant et al. 2015)
EF060363	T2	Italy	Europe	(La Morgia et al. 2008)
FJ238094	T2	Armenia	Southwest Asia	Family Tree
FJ656215	T2	Russia	Europe	HGDP - Zheng, et al. (unpublished)
GU565218	T2	unknown	unknown	Family Tree
HM852766	T2	Armenia	Southwest Asia	(Schönberg et al. 2011)
HM852781	T2	Armenia	Southwest Asia	(Schönberg et al. 2011)
HM852899	T2	Georgia	Southwest Asia	(Schönberg et al. 2011)
JQ702108	T2	Germany	Europe	(Behar et al. 2012)
JQ702594	T2	Scotland	Europe	(Behar et al. 2012)
JQ703754	T2	Scotland	Europe	(Behar et al. 2012)
JQ705133	T2	Ireland	Europe	(Behar et al. 2012)
KC911414	T2	Iran: Persian	Southwest Asia	(Derenko et al. 2013)
KC911414	T2	Iran: Persian	Southwest Asia	(Derenko et al. 2013)
KJ445844	T2	Palestine	Southwest Asia	HGDP - Zheng, et al. (unpublished)
KJ445845	T2	Israel: Bedouin	Southwest Asia	HGDP - Zheng, et al. (unpublished)
KJ445849	T2	Palestine	Southwest Asia	HGDP - Zheng, et al. (unpublished)
KJ445856	T2	Palestine	Southwest Asia	HGDP - Zheng, et al. (unpublished)
KJ445862	T2	Russia: Adygei, Caucasus	Southwest Asia	HGDP - Zheng, et al. (unpublished)
KJ445863	T2	Russia: Adygei, Caucasus	Southwest Asia	HGDP - Zheng, et al. (unpublished)
KJ445851	T2	Pakistan: Sindhi	South Asia - West	HGDP - Zheng, et al. (unpublished)
KJ445855	T2	Pakistan: Burusho	South Asia - West	HGDP - Zheng, et al. (unpublished)
KJ445860	T2	Pakistan: Kalash	South Asia - West	HGDP - Zheng, et al. (unpublished)
KJ445861	T2	Pakistan: Brahui	South Asia - West	HGDP - Zheng, et al. (unpublished)
NA20901	T2	GIH	South Asia - West	(Sudmant et al. 2015)
AY289073	U1	India: Koraga	South Asia - South	(Ingman and Gyllensten 2003)
AY714038	U1	India: Uttar Pradesh	South Asia - Central	(Palanichamy et al. 2004)
AY714042	U1	India: Andhra Pradesh	South Asia - South	(Palanichamy et al. 2004)
EF556194	U1	Cochin Jew	South Asia	(Behar et al. 2008a)
HG03780	U1	ITU	South Asia - South	(Sudmant et al. 2015)
HG03862	U1	ITU	South Asia - South	(Sudmant et al. 2015)
HG03886	U1	STU	South Asia - South	(Sudmant et al. 2015)
HG04098	U1	ITU	South Asia - South	(Sudmant et al. 2015)
HM156682	U1	India	South Asia	(Govindaraj et al. 2011)

Table S2. (*continued*)

Sample	Haplogroup	Origin (Population)	Region	Reference
AY882396	U1	Adygei	Southwest Asia	(Achilli et al. 2004)
EF556161	U1	Iran: Jew	Southwest Asia	(Behar et al. 2008a)
EF661008	U1	Italy	Europe	(Gasparre et al. 2007)
FJ748753	U1	Tibet	East Asia	(Ji et al. 2012)
GU218692	U1	Greece	Europe	Family Tree
HM852789	U1	Azeri	Southwest Asia	(Schönberg et al. 2011)
HM852790	U1	Azeri	Southwest Asia	(Schönberg et al. 2011)
HM852847	U1	Turk	Southwest Asia	(Schönberg et al. 2011)
KC477757	U1	Iraq - Kurd	Southwest Asia	Family Tree
KC911306	U1	Iran: Persian	Southwest Asia	(Derenko et al. 2013)
KC911344	U1	Iran: Armenian	Southwest Asia	(Derenko et al. 2013)
KC911457	U1	Iran: Azeri	Southwest Asia	(Derenko et al. 2013)
KC911527	U1	Iran: Qashqai	Southwest Asia	(Derenko et al. 2013)
KJ445918	U1	Russia: Adygei, Caucasus	Southwest Asia	HGDP - Zheng, et al. (unpublished)
KJ445919	U1	Israel: Druze	Southwest Asia	HGDP - Zheng, et al. (unpublished)
KJ445921	U1	Palestine	Southwest Asia	HGDP - Zheng, et al. (unpublished)
KJ445920	U1	Pakistan: Hazara	South Asia - West	HGDP - Zheng, et al. (unpublished)
NA21097	U1	GIH	South Asia - West	(Sudmant et al. 2015)
AY714026	U2	India: Andhra Pradesh	South Asia - South	(Palanichamy et al. 2004)
AY714040	U2	India: Andhra Pradesh	South Asia - South	(Palanichamy et al. 2004)
AY714049	U2	India: Andhra Pradesh	South Asia - South	(Palanichamy et al. 2004)
HG03960	U2	ITU	South Asia - South	(Sudmant et al. 2015)
HM156683	U2	India	South Asia	(Govindaraj et al. 2011)
HM156688	U2	India	South Asia	(Govindaraj et al. 2011)
AF381995	U2	Jordan	Southwest Asia	(Maca-Meyer et al. 2001)
EF528162	U2	unknown	unknown	Family Tree
EF661006	U2	Italy	Europe	(Gasparre et al. 2007)
FJ493504	U2	Russia	Europe	(Sukernik et al. 2012)
FJ828532	U2	unknown	unknown	Family Tree
JQ701890	U2	unknown	unknown	(Behar et al. 2012)
JQ701947	U2	unknown	unknown	(Behar et al. 2012)
JQ702004	U2	unknown	unknown	(Behar et al. 2012)
JQ702106	U2	unknown	unknown	(Behar et al. 2012)
JQ702663	U2	unknown	unknown	(Behar et al. 2012)
JQ705559	U2	unknown	unknown	(Behar et al. 2012)
JQ705711	U2	England	Europe	(Behar et al. 2012)
JQ705900	U2	England	Europe	(Behar et al. 2012)
KC911479	U2	Iran: Persian	Southwest Asia	(Derenko et al. 2013)
KC911513	U2	Iran: Qashqai	Southwest Asia	(Derenko et al. 2013)
NA20904	U2	GIH	South Asia - West	(Sudmant et al. 2015)
AY714023	U3	India: Uttar Pradesh	South Asia - Central	(Palanichamy et al. 2004)

Table S2. (*continued*)

Sample	Haplogroup	Origin (Population)	Region	Reference
HM852797	U3	Azeri	Southwest Asia	(Schönberg et al. 2011)
HM852819	U3	Iran	Southwest Asia	(Schönberg et al. 2011)
HM852891	U3	Georgia	Southwest Asia	(Schönberg et al. 2011)
JQ704130	U3	Hungary	Europe	(Behar et al. 2012)
KC911459	U3	Iran: Qashqai	Southwest Asia	(Derenko et al. 2013)
KC911334	U4	Iran: Qashqai	Southwest Asia	(Derenko et al. 2013)
HG03897	U5	STU	South Asia - South	(Sudmant et al. 2015)
HG04159	U5	BEB	South Asia - East	(Sudmant et al. 2015)
JF906114	U5	India	South Asia	Family Tree
DQ156210	U5	Spain	Europe	(Montiel-Sosa et al. 2006)
EU597527	U5	Palestine	Southwest Asia	(Hartmann et al. 2009)
GU296543	U5	Poland	Europe	(Malyarchuk et al. 2010b)
GU296570	U5	Czech Republic	Europe	(Malyarchuk et al. 2010b)
GU296581	U5	Belarus	Europe	(Malyarchuk et al. 2010b)
GU296583	U5	Belarus	Europe	(Malyarchuk et al. 2010b)
HM852782	U5	Armenia	Southwest Asia	(Schönberg et al. 2011)
JQ582984	U5	England	Europe	Family Tree
JQ702310	U5	unknown	unknown	(Behar et al. 2012)
JQ702913	U5	unknown	unknown	(Behar et al. 2012)
JQ704112	U5	Scotland	Europe	(Behar et al. 2012)
JQ705429	U5	England	Europe	(Behar et al. 2012)
KC569552	U5	USA	Europe	Family Tree
KC911325	U5	Iran: Qashqai	Southwest Asia	(Derenko et al. 2013)
KC911503	U5	Iran: Qashqai	Southwest Asia	(Derenko et al. 2013)
KC911532	U5	Iran: Qashqai	Southwest Asia	(Derenko et al. 2013)
NA20854	U5	GIH	South Asia - West	(Sudmant et al. 2015)
AY714004	U7	India: Uttar Pradesh	South Asia - Central	(Palanichamy et al. 2004)
AY714013	U7	India: Uttar Pradesh	South Asia - Central	(Palanichamy et al. 2004)
AY714014	U7	India: Uttar Pradesh	South Asia - Central	(Palanichamy et al. 2004)
AY882391	U7	Pakistan	South Asia - West	(Achilli et al. 2004)
GU213243	U7	India: Uttar Pradesh	South Asia - Central	(Palanichamy et al. 2015)
GU213244	U7	India: Tamil Nadu	South Asia - South	(Palanichamy et al. 2015)
GU213245	U7	Bangladesh	South Asia - East	(Palanichamy et al. 2015)
GU213246	U7	Bangladesh	South Asia - East	(Palanichamy et al. 2015)
GU213247	U7	India: Uttar Pradesh	South Asia - Central	(Palanichamy et al. 2015)
GU213248	U7	India: Uttar Pradesh	South Asia - Central	(Palanichamy et al. 2015)
GU213249	U7	India: Tamil Nadu	South Asia - South	(Palanichamy et al. 2015)
GU213250	U7	India: Tamil Nadu	South Asia - South	(Palanichamy et al. 2015)
GU213251	U7	India: West Bengal	South Asia - East	(Palanichamy et al. 2015)
GU213252	U7	India: West Bengal	South Asia - East	(Palanichamy et al. 2015)
GU213253	U7	India: Tamil Nadu	South Asia - South	(Palanichamy et al. 2015)
GU213254	U7	India: Tamil Nadu	South Asia - South	(Palanichamy et al. 2015)
GU480003	U7	India: Madhya Pradesh	South Asia - Central	(Sharma et al. 2012)
HG02494	U7	PJL	South Asia - West	(Sudmant et al. 2015)

Table S2. (*continued*)

Sample	Haplogroup	Origin (Population)	Region	Reference
HG02648	U7	PJL	South Asia - West	(Sudmant et al. 2015)
HG02694	U7	PJL	South Asia - West	(Sudmant et al. 2015)
HG02724	U7	PJL	South Asia - West	(Sudmant et al. 2015)
HG02731	U7	PJL	South Asia - West	(Sudmant et al. 2015)
HG02775	U7	PJL	South Asia - West	(Sudmant et al. 2015)
HG03681	U7	STU	South Asia - South	(Sudmant et al. 2015)
HG03696	U7	STU	South Asia - South	(Sudmant et al. 2015)
HG03711	U7	STU	South Asia - South	(Sudmant et al. 2015)
HG04056	U7	ITU	South Asia - South	(Sudmant et al. 2015)
HG04134	U7	BEB	South Asia - East	(Sudmant et al. 2015)
AF382011	U7	Spain	Europe	(Maca-Meyer et al. 2001)
AY339547	U7	Finland	Europe	(Finnilä et al. 2001)
AY339548	U7	Finland	Europe	(Finnilä et al. 2001)
KJ445973	U7	Pakistan: Brahui	South Asia - West	HGDP - Zheng, et al. (unpublished)
KJ445974	U7	Pakistan: Brahui	South Asia - West	HGDP - Zheng, et al. (unpublished)
EF556179	U7	Iran: Jew	Southwest Asia	(Behar et al. 2008a)
EU445683	U7	Italy: Isle of Elba	Europe	(Brisighelli et al. 2009)
EU445684	U7	Italy: Isle of Elba	Europe	(Brisighelli et al. 2009)
EU445685	U7	Italy: Isle of Elba	Europe	(Brisighelli et al. 2009)
EU445686	U7	Italy: Isle of Elba	Europe	(Brisighelli et al. 2009)
EU445687	U7	Italy: Isle of Elba	Europe	(Brisighelli et al. 2009)
EU445688	U7	Italy: Isle of Elba	Europe	(Brisighelli et al. 2009)
EU445689	U7	Italy: Isle of Elba	Europe	(Brisighelli et al. 2009)
EU445690	U7	Italy: Isle of Elba	Europe	(Brisighelli et al. 2009)
EU445691	U7	Italy: Isle of Elba	Europe	(Brisighelli et al. 2009), 2009
EU597503	U7	Israel: Bedouin	Southwest Asia	(Hartmann et al. 2009)
FJ858878	U7	Russia: North-West Siberia	East Asia	(Sukernik et al. 2012)
GQ176284	U7	Finland	Europe	Family Tree
GU213236	U7	Russia	Europe	(Palanichamy et al. 2015)
GU213237	U7	Kalmyk	Europe	(Palanichamy et al. 2015)
GU213238	U7	Russia	Europe	(Palanichamy et al. 2015)
GU213239	U7	Iran: Persian	Southwest Asia	(Palanichamy et al. 2015)
GU213240	U7	Bargut	East Asia	(Palanichamy et al. 2015)
GU213241	U7	Slovakia	Europe	(Palanichamy et al. 2015)
GU213242	U7	Russia	Europe	(Palanichamy et al. 2015)
KJ445975	U7	Pakistan: Burusho	South Asia - West	HGDP - Zheng, et al. (unpublished)
KJ445976	U7	Pakistan: Pathan	South Asia - West	HGDP - Zheng, et al. (unpublished)
KJ445977	U7	Pakistan: Hazara	South Asia - West	HGDP - Zheng, et al. (unpublished)
KJ445979	U7	Pakistan: Brahui	South Asia - West	HGDP - Zheng, et al. (unpublished)

Table S2. (*continued*)

Sample	Haplogroup	Origin (Population)	Region	Reference
KJ445980	U7	Pakistan: Burusho	South Asia - West	HGDP - Zheng, et al. (unpublished)
GU213255	U7	unknown	unknown	(Palanichamy et al. 2015)
GU213256	U7	unknown	unknown	(Palanichamy et al. 2015)
GU213257	U7	unknown	unknown	(Palanichamy et al. 2015)
GU213258	U7	unknown	unknown	(Palanichamy et al. 2015)
GU213259	U7	unknown	unknown	(Palanichamy et al. 2015)
GU327373	U7	unknown	unknown	Zhang, et al. (unpublished)
HM852777	U7	Armenia	Southwest Asia	(Schönberg et al. 2011)
HM852788	U7	Azeri	Southwest Asia	(Schönberg et al. 2011)
HM852791	U7	Azeri	Southwest Asia	(Schönberg et al. 2011)
HM852801	U7	Azeri	Southwest Asia	(Schönberg et al. 2011)
HM852823	U7	Iran	Southwest Asia	(Schönberg et al. 2011)
HM852853	U7	Turk	Southwest Asia	(Schönberg et al. 2011)
JQ701923	U7	unknown	unknown	(Behar et al. 2012)
JQ703913	U7	unknown	unknown	(Behar et al. 2012)
JQ703978	U7	Poland: Ashkenazi Jew	Europe	(Behar et al. 2012)
JQ704100	U7	unknown	unknown	(Behar et al. 2012)
JQ705198	U7	unknown	unknown	(Behar et al. 2012)
JQ705966	U7	Iraq: Kirkuk, Mizrachi	Southwest Asia	(Behar et al. 2012)
KC911278	U7	Iran: Persian	Southwest Asia	(Derenko et al. 2013)
KC911287	U7	Iran: Persian	Southwest Asia	(Derenko et al. 2013)
KC911288	U7	Iran: Persian	Southwest Asia	(Derenko et al. 2013)
KC911299	U7	Iran: Qashqai	Southwest Asia	(Derenko et al. 2013)
KC911347	U7	Iran: Lur	Southwest Asia	(Derenko et al. 2013)
KC911392	U7	Iran: Persian	Southwest Asia	(Derenko et al. 2013)
KC911402	U7	Iran: Lur	Southwest Asia	(Derenko et al. 2013)
KC911415	U7	Iran: Qashqai	Southwest Asia	(Derenko et al. 2013)
KC911448	U7	Iran: Qashqai	Southwest Asia	(Derenko et al. 2013)
KC911455	U7	Iran: Persian	Southwest Asia	(Derenko et al. 2013)
KC911470	U7	Iran: Persian	Southwest Asia	(Derenko et al. 2013)
KC911508	U7	Iran: Persian	Southwest Asia	(Derenko et al. 2013)
KC911509	U7	Iran: Kurd	Southwest Asia	(Derenko et al. 2013)
KC911526	U7	Iran: Persian	Southwest Asia	(Derenko et al. 2013)
KC911553	U7	Iran: Persian	Southwest Asia	(Derenko et al. 2013)
KC911563	U7	Iran: Persian	Southwest Asia	(Derenko et al. 2013)
KC911615	U7	Iran: Persian	Southwest Asia	(Derenko et al. 2013)
KC911620	U7	Iran: Persian	Southwest Asia	(Derenko et al. 2013)
KC911622	U7	Iran: Persian	Southwest Asia	(Derenko et al. 2013)
KJ445972	U7	Israel: Bedouin	Southwest Asia	HGDP - Zheng, et al. (unpublished)
NA18130	U7	Han Chinese	East Asia	(Sudmant et al. 2015)
KP763839	U7	India: Uttar Pradesh	South Asia	(Palanichamy et al. 2015)
NA20862	U7	GIH	South Asia - West	(Sudmant et al. 2015)

Table S2. (*continued*)

Sample	Haplogroup	Origin (Population)	Region	Reference
NA20889	U7	GIH	South Asia - West	(Sudmant et al. 2015)
NA21107	U7	GIH	South Asia - West	(Sudmant et al. 2015)
NA21113	U7	GIH	South Asia - West	(Sudmant et al. 2015)
NA21130	U7	GIH	South Asia - West	(Sudmant et al. 2015)
HM852759	U8	Armenia	Southwest Asia	(Schönberg et al. 2011)
JQ702759	U8	unknown	unknown	(Behar et al. 2012)
AY882390	U9	Pakistan	South Asia - West	(Achilli et al. 2004)
EU597540	U9	Pakistan: Pathan	South Asia - West	(Hartmann et al. 2009)
FJ770944	U9	India: Andhra Pradesh	South Asia	(Fornarino et al. 2009)
HG03777	U9	ITU	South Asia - South	(Sudmant et al. 2015)
HG03916	U9	BEB	South Asia - East	(Sudmant et al. 2015)
HG04118	U9	ITU	South Asia - South	(Sudmant et al. 2015)
KJ445948	U9	Pakistan: Makrani	South Asia - West	HGDP - Zheng, et al. (unpublished)
KP763838	U9	India	South Asia	(Palanichamy et al. 2015)
KP763849	U9	India	South Asia	(Palanichamy et al. 2015)
AY882389	U9	Ethiopia	Africa	(Achilli et al. 2004)
GU990521	U9	USA	America	Family Tree
NA21108	U9	GIH	South Asia - West	(Sudmant et al. 2015)
AY713979	V	India: Uttar Pradesh	South Asia - Central	(Palanichamy et al. 2004)
AY195750	V	unknown	unknown	(Mishmar et al. 2003)
AY495307	V	Europe	Europe	(Coble et al. 2004)
AY495309	V	Europe	Europe	(Coble et al. 2004)
AY495311	V	Europe	Europe	(Coble et al. 2004)
AY495315	V	Europe	Europe	(Coble et al. 2004)
AY495320	V	Europe	Europe	(Coble et al. 2004)
AY495322	V	Europe	Europe	(Coble et al. 2004)
AY495326	V	Europe	Europe	(Coble et al. 2004)
JN630803	V	Spain: Jew	Europe	Family Tree
KC911406	V	Iran: Persian	Southwest Asia	(Derenko et al. 2013)
JQ705658	V2	England	Europe	(Behar et al. 2012)
JQ245778	W	Yemen	Southwest Asia	(Fernandes et al. 2012)
KC911341	W	Iran: Persian	Southwest Asia	(Derenko et al. 2013)
AY714039	W1	India: Uttar Pradesh	South Asia - Central	(Palanichamy et al. 2004)
HG03228	W1	PJL	South Asia - West	(Sudmant et al. 2015)
EU257638	W1	unknown	unknown	Family Tree
EU558696	W1	England	Europe	Family Tree
FJ472633	W1	unknown	unknown	Family Tree
GU123002	W1	Russia	Europe	(Malyarchuk et al. 2010a)
JQ245768	W1	Turkey	Southwest Asia	(Fernandes et al. 2012)
JQ702050	W1	unknown	unknown	(Behar et al. 2012)
JQ702450	W1	unknown	unknown	(Behar et al. 2012)
JQ706021	W1	United Kingdom	Europe	(Behar et al. 2012)
JQ898579	W1	Ashkenazi Jew	Europe	Family Tree

Table S2. (*continued*)

Sample	Haplogroup	Origin (Population)	Region	Reference
KC911433	W1	Iran: Persian	Southwest Asia	(Derenko et al. 2013)
KC911537	W1	Iran: Qashqai	Southwest Asia	(Derenko et al. 2013)
AY714043	W3	India: Uttar Pradesh	South Asia - Central	(Palanichamy et al. 2004)
GU002155	W3	India: Punjab	South Asia	Family Tree
HG03778	W3	ITU	South Asia - South	(Sudmant et al. 2015)
HG03785	W3	ITU	South Asia - South	(Sudmant et al. 2015)
HG03786	W3	ITU	South Asia - South	(Sudmant et al. 2015)
HG03815	W3	BEB	South Asia - East	(Sudmant et al. 2015)
HG04026	W3	ITU	South Asia - South	(Sudmant et al. 2015)
HG04156	W3	BEB	South Asia - East	(Sudmant et al. 2015)
KJ445931	W3	Pakistan: Pathan	South Asia - West	HGDP - Zheng, et al. (unpublished)
KJ445932	W3	Pakistan: Pathan	South Asia - West	HGDP - Zheng, et al. (unpublished)
KJ445933	W3	Pakistan: Sindhi	South Asia - West	HGDP - Zheng, et al. (unpublished)
KJ445934	W3	Pakistan: Pathan	South Asia - West	HGDP - Zheng, et al. (unpublished)
JQ245741	W3	Azerbaijan	Southwest Asia	(Fernandes et al. 2012)
JQ245760	W3	Turkey: Kurd	Southwest Asia	(Fernandes et al. 2012)
JQ702421	W3	unknown	unknown	(Behar et al. 2012)
JQ705313	W3	Austria	Europe	(Behar et al. 2012)
AY714018	W4	India: Uttar Pradesh	South Asia - Central	(Palanichamy et al. 2004)
NA20858	W4	GIH	South Asia - West	(Sudmant et al. 2015)
GU045487	W4	Ulter Scots	Europe	Family Tree
JQ245758	W4	Turkey: Kurd	Southwest Asia	(Fernandes et al. 2012)
HG02734	W6	PJL	South Asia - West	(Sudmant et al. 2015)
HG04239	W6	ITU	South Asia - South	(Sudmant et al. 2015)
NA20851	W6	GIH	South Asia - West	(Sudmant et al. 2015)
JF902025	W6	England	Europe	Family Tree
JQ245723	W6	Russia: North Ossetia	Southwest Asia	(Fernandes et al. 2012)
JQ245736	W6	Russia: North Ossetia	Southwest Asia	(Fernandes et al. 2012)
JQ245769	W6	Turkey	Southwest Asia	(Fernandes et al. 2012)
JQ705793	W6	England	Europe	(Behar et al. 2012)
KC911604	W6	Iran: Persian	Southwest Asia	(Derenko et al. 2013)
HG02733	X	PJL	South Asia - West	(Sudmant et al. 2015)
NA20886	X	GIH	South Asia - West	(Sudmant et al. 2015)
NA20898	X	GIH	South Asia - West	(Sudmant et al. 2015)
EU600318	X	Druze	Southwest Asia	(Shlush et al. 2008)
HM852799	X	Azeri	Southwest Asia	(Schönberg et al. 2011)
JQ245765	X	Turkey	Southwest Asia	(Fernandes et al. 2012)
KC911291	X	Iran: Qashqai	Southwest Asia	(Derenko et al. 2013)

References:

Achilli A, Rengo C, Magri C, et al (2004) The molecular dissection of mtDNA haplogroup H confirms that the Franco-Cantabrian glacial refuge was a major source for the European gene pool. Am J Hum Genet 75:910–918.

Al-Abri A, Podgorná E, Rose JI, et al (2012) Pleistocene-Holocene boundary in Southern Arabia from the perspective of human mtDNA variation. Am J Phys Anthropol 149:291–8.

Behar DM, Metspalu E, Kivisild T, et al (2008a) Counting the founders: the matrilineal genetic ancestry of the Jewish Diaspora. PLoS One 3:e2062.

Behar DM, Metspalu E, Kivisild T, et al (2006) The matrilineal ancestry of Ashkenazi Jewry: portrait of a recent founder event. Am J Hum Genet 78:487–97.

Behar DM, van Oven M, Rosset S, et al (2012) A "Copernican" reassessment of the human mitochondrial DNA tree from its root. Am J Hum Genet 90:675–84.

Behar DM, Villems R, Soodyall H, et al (2008b) The Dawn of Human Matrilineal Diversity. Am J Hum Genet 82:1130–1140.

Bertolin C, Magri C, Barlati S, et al (2011) Analysis of complete mitochondrial genomes of patients with schizophrenia and bipolar disorder. J Hum Genet 56:869–72.

Brisighelli F, Capelli C, Alvarez-Iglesias V, et al (2009) The Etruscan timeline: a recent Anatolian connection. Eur J Hum Genet 17:693–6.

Cerný V, Mulligan CJ, Fernandes V, et al (2011) Internal diversification of mitochondrial haplogroup R0a reveals post-last glacial maximum demographic expansions in South Arabia. Mol Biol Evol 28:71–8.

Coble MD, Just RS, O'Callaghan JE, et al (2004) Single nucleotide polymorphisms over the entire mtDNA genome that increase the power of forensic testing in Caucasians. Int J Legal Med 118:137–46.

Costa MD, Pereira JB, Pala M, et al (2013) A substantial prehistoric European ancestry amongst Ashkenazi maternal lineages. Nat Commun 4:2543.

Derenko M, Malyarchuk B, Bahmanimehr A, et al (2013) Complete Mitochondrial DNA Diversity in Iranians. PLoS One 8:e80673.

Derenko M, Malyarchuk B, Denisova G, et al (2014) Western Eurasian ancestry in modern Siberians based on mitogenomic data. BMC Evol Biol 14:217.

Detjen AK, Tinschert S, Kaufmann D, et al (2007) Analysis of mitochondrial DNA in discordant monozygotic twins with neurofibromatosis type 1. Twin Res Hum Genet 10:486–95.

Fernandes V, Alshamali F, Alves M, et al (2012) The Arabian cradle: mitochondrial relicts of the first steps along the southern route out of Africa. Am J Hum Genet 90:347–55.

Finnilä S, Lehtonen MS, Majamaa K (2001) Phylogenetic network for European mtDNA. Am J Hum Genet 68:1475–84.

Fornarino S, Pala M, Battaglia V, et al (2009) Mitochondrial and Y-chromosome diversity of the Tharus (Nepal): a reservoir of genetic variation. BMC Evol Biol 9:154.

Fraumene C, Belle EMS, Castrì L, et al (2006) High resolution analysis and phylogenetic network construction using complete mtDNA sequences in sardinian genetic isolates. Mol Biol Evol 23:2101–11.

Gasparre G, Porcelli AM, Bonora E, et al (2007) Disruptive mitochondrial DNA mutations in complex I subunits are markers of oncocytic phenotype in thyroid tumors. Proc Natl Acad Sci U S A 104:9001–6.

Govindaraj P, Khan NA, Gopalakrishna P, et al (2011) Mitochondrial dysfunction and genetic heterogeneity in chronic periodontitis. Mitochondrion 11:504–12.

Hartmann A, Thieme M, Nanduri LK, et al (2009) Validation of microarray-based resequencing of 93 worldwide mitochondrial genomes. Hum Mutat 30:115–22.

Ingman M, Gyllensten U (2003) Mitochondrial genome variation and evolutionary history of Australian and New Guinean aborigines. Genome Res 13:1600–6.

Ji F, Sharpley MS, Derbeneva O, et al (2012) Mitochondrial DNA variant associated with Leber hereditary optic neuropathy and high-altitude Tibetans. Proc Natl Acad Sci U S A 109:7391–6.

Kong Q-P, Sun C, Wang H-W, et al (2011) Large-scale mtDNA screening reveals a surprising matrilineal complexity in east Asia and its implications to the peopling of the region. Mol Biol Evol 28:513–22.

La Morgia C, Achilli A, Iommarini L, et al (2008) Rare mtDNA variants in Leber hereditary optic neuropathy families with recurrence of myoclonus. Neurology 70:762–70.

Li S, Besenbacher S, Li Y, et al (2014) Variation and association to diabetes in 2000 full mtDNA sequences mined from an exome study in a Danish population. Eur J Hum Genet 22:1040–5.

Maca-Meyer N, Gonzalez A, Larruga J, et al (2001) Major genomic mitochondrial lineages delineate early human expansions. BMC Genet 2:13.

Malyarchuk BA, Perkova MA, Derenko M V, et al (2008a) Mitochondrial DNA variability in Slovaks, with application to the Roma origin. Ann Hum Genet 72:228–40.

Malyarchuk B, Derenko M, Denisova G, Kravtsova O (2010a) Mitogenomic diversity in Tatars from the Volga-Ural region of Russia. Mol Biol Evol 27:2220–6.

Malyarchuk B, Derenko M, Grzybowski T, et al (2010b) The peopling of Europe from the mitochondrial haplogroup U5 perspective. PLoS One 5:e10285.

Malyarchuk B, Grzybowski T, Derenko M, et al (2008b) Mitochondrial DNA phylogeny in Eastern and Western Slavs. Mol Biol Evol 25:1651–8.

Mishmar D, Ruiz-Pesini E, Golik P, et al (2003) Natural selection shaped regional mtDNA variation in humans. Proc Natl Acad Sci U S A 100:171–6.

Montiel-Sosa F, Ruiz-Pesini E, Enríquez JA, et al (2006) Differences of sperm motility in mitochondrial DNA haplogroup U sublineages. Gene 368:21–7.

Pala M, Olivieri A, Achilli A, et al (2012) Mitochondrial DNA signals of late glacial recolonization of europe from near eastern refugia. Am J Hum Genet 90:915–924.

Palanichamy MG, Mitra B, Zhang C-L, et al (2015) West Eurasian mtDNA lineages in India: an insight into the spread of the Dravidian language and the origins of the caste system. Hum Genet 134:637–47.

Palanichamy MG, Sun C, Agrawal S, et al (2004) Phylogeny of mitochondrial DNA macrohaplogroup N in India, based on complete sequencing: implications for the peopling of South Asia. Am J Hum Genet 75:966–78.

Peng M-S, Quang HH, Dang KP, et al (2010) Tracing the Austronesian footprint in Mainland Southeast Asia: a perspective from mitochondrial DNA. Mol Biol Evol 27:2417–30.

Schönberg A, Theunert C, Li M, et al (2011) High-throughput sequencing of complete human mtDNA genomes from the Caucasus and West Asia: high diversity and demographic inferences. Eur J Hum Genet 19:988–94.

Sharma G, Tamang R, Chaudhary R, et al (2012) Genetic affinities of the central Indian tribal populations. PLoS One 7:e32546.

Shlush LI, Behar DM, Yudkovsky G, et al (2008) The Druze: a population genetic refugium of the Near East. PLoS One 3:e2105.

Soares P, Alshamali F, Pereira JB, et al (2012) The Expansion of mtDNA Haplogroup L3 within and out of Africa. Mol Biol Evol 29:915–27.

Sudmant PH, Rausch T, Gardner EJ, et al (2015) An integrated map of structural variation in 2,504 human genomes. Nature 526:75–81.

Sukernik RI, Volodko N V, Mazunin IO, et al (2012) Mitochondrial genome diversity in the Tubalar, Even, and Ulchi: contribution to prehistory of native Siberians and their affinities to Native Americans. Am J Phys Anthropol 148:123–38.

Torroni A, Achilli A, Macaulay V, et al (2006) Harvesting the fruit of the human mtDNA tree. Trends Genet 22:339–45.

Table S3. Dataset used for the GW analyses. (a) Modern dataset. Populations marked with three asterisks (***) were added to the dataset exclusively for ADMIXTURE and sNMF runs, thereby being absent from the PCA. Population codes: BEB – Bengali from Bangladesh; GIH – Gujarati Indian from Houston, Texas; ITU – Indian Telugu from the UK; PJL – Punjabi from Lahore, Pakistan; STU – Sri Lankan Tamil from the UK; CHB – Han Chinese in Beijing, China; TSI – Tuscans from Italy; IRY – Yoruba in Ibadan, Nigeria. (b) Yamnaya samples.

(a) Modern dataset

Region	Population	Reference	n
Africa	YRI ***	(Sudmant et al. 2015)	108
Europe	TSI ***	(Sudmant et al. 2015)	107
Anatolia + Caucasus	Abkhazians	(Yunusbayev et al. 2012)	20
	Adygei	(Li et al. 2008)	17
	Armenians	(Behar et al. 2010; Yunusbayev et al. 2012)	19
	Azerbaijani Jews	(Behar et al. 2010)	8
	Balkars	(Yunusbayev et al. 2012)	19
	Chechens	(Yunusbayev et al. 2012)	20
	Cypriots	(Behar et al. 2010)	12
	Georgian Jews	(Behar et al. 2010)	4
	Georgians	(Behar et al. 2010)	20
	Kumyks	(Yunusbayev et al. 2012)	13
	Lezgins	(Behar et al. 2010)	18
	Nogais	(Yunusbayev et al. 2012)	16
	North Ossetians	(Yunusbayev et al. 2012)	15
	Turks	(Behar et al. 2010)	19
Central Asia	Tajiks	(Yunusbayev et al. 2012)	15
	Turkmens	(Yunusbayev et al. 2012)	12
	Uzbekistani Jews	(Behar et al. 2010)	2
	Uzbeks	(Behar et al. 2010; Di Cristofaro et al. 2013)	15
Arabia + Near East	Bedouins	(Li et al. 2008)	45
	Druze	(Li et al. 2008)	42
	Iranian Jews	(Behar et al. 2010)	20
	Iranians	(Behar et al. 2010)	4
	Iraqi Jews	(Behar et al. 2010)	11

Table S3. (*continued*)

Region	Population	Reference	n
Arabia + Near East	Jordanians	(Behar et al. 2010)	20
	Lebanese	(Haber et al. 2013)	75
	Lebanese	(Behar et al. 2010)	7
	Palestinians	(Li et al. 2008)	46
	Samaritans	(Chaubey et al. 2011)	3
	Saudis	(Behar et al. 2010)	19
	Syrians	(Behar et al. 2010)	16
	Yemeni	(Behar et al. 2010)	10
	Yemeni Jews	(Behar et al. 2010)	15
South Asia	Asur	(Chaubey et al. 2011)	2
	Balochi	(Li et al. 2008)	24
	BEB + Bengali	(Chaubey et al. 2011; Sudmant et al. 2015)	87 (86+1)
	Bene Israel	(Chaubey et al. 2011)	4
	Bhunjia	(Chaubey et al. 2011)	1
	Bonda	(Chaubey et al. 2011)	4
	Brahmins_TN (South)	(Chaubey et al. 2011)	2
	Brahmins_UP/UTT (Central)	(Chaubey et al. 2011)	9 (8/1)
	Brahui	(Li et al. 2008)	25
	Burusho	(Li et al. 2008)	25
	Chamar	(Chaubey et al. 2011)	10
	Chenchus	(Metspalu et al. 2011)	4
	Cochin Jews	(Behar et al. 2010)	4
	Dharkars	(Chaubey et al. 2011)	12
	Dhurwa	(Chaubey et al. 2011)	1
	Dusadh	(Chaubey et al. 2011)	10
	Gadaba	(Chaubey et al. 2011)	1
	GIH + Gujaratis	(Altshuler et al. 2010; Sudmant et al. 2015)	103 (22+81)
	Gond	(Metspalu et al. 2011)	4
	Halakipikki	(Metspalu et al. 2011)	4
	Ho	(Chaubey et al. 2011)	5
	ITU	(Sudmant et al. 2015)	102
	Juang	(Chaubey et al. 2011)	2
	Kalash	(Li et al. 2008)	23
	Kanjars	(Chaubey et al. 2011)	8
	Kharia	(Chaubey et al. 2011)	2

Table S3. (*continued*)

Region	Population	Reference	n
South Asia	Khasi	(Chaubey et al. 2011)	3
	Kol	(Chaubey et al. 2011)	17
	Kshatriya	(Chaubey et al. 2011)	7
	Kurumba	(Metspalu et al. 2011)	4
	Lambadi	(Chaubey et al. 2011)	1
	Low Caste_TN (South)	(Chaubey et al. 2011)	2
	Low Caste_UP (Central)	(Chaubey et al. 2011)	5
	Makrani	(Li et al. 2008)	25
	Malayan	(Behar et al. 2010)	2
	Mawasi	(Chaubey et al. 2011)	1
	Meena	(Chaubey et al. 2011)	1
	Meghwal	(Chaubey et al. 2011)	1
	Muslim	(Chaubey et al. 2011)	5
	Nihali	(Chaubey et al. 2011)	2
	North Kannadi	(Behar et al. 2010)	9
	Paniya	(Behar et al. 2010)	4
	Pathan	(Li et al. 2008)	22
	Piramalai Kallars	(Chaubey et al. 2011)	8
	PJL	(Sudmant et al. 2015)	96
	Pulliyar	(Metspalu et al. 2011)	5
	Sakilli	(Behar et al. 2010)	4
	Santhal	(Chaubey et al. 2011)	1
	Savara	(Chaubey et al. 2011)	2
	Sindhi	(Li et al. 2008)	24
	STU	(Sudmant et al. 2015)	102
	Tharus	(Chaubey et al. 2011)	2
	Velmas	(Metspalu et al. 2011)	10
East Asia	CHB ***	(Sudmant et al. 2015)	103
**(b) Yamanaya**			
-	Yamanaya	(Haak et al. 2015)	9

References

Altshuler DM, Gibbs RA, Peltonen L, et al (2010) Integrating common and rare genetic variation in diverse human populations. Nature 467:52–8.

Behar DM, Yunusbayev B, Metspalu M, et al (2010) The genome-wide structure of the Jewish people. Nature 466:238–42.

Chaubey G, Metspalu M, Choi Y, et al (2011) Population genetic structure in Indian Austroasiatic speakers: the role of landscape barriers and sex-specific admixture. Mol Biol Evol 28:1013–24.

Di Cristofaro J, Pennarun E, Mazières S, et al (2013) Afghan Hindu Kush: where Eurasian sub-continent gene flows converge. PLoS One 8:e76748.

Haak W, Lazaridis I, Patterson N, et al (2015) Massive migration from the steppe was a source for Indo-European languages in Europe. Nature 207–211. doi: 10.1038/nature14317

Haber M, Gauguier D, Youhanna S, et al (2013) Genome-wide diversity in the levant reveals recent structuring by culture. PLoS Genet 9:e1003316.

Li JZ, Absher DM, Tang H, et al (2008) Worldwide human relationships inferred from genome-wide patterns of variation. Science 319:1100–4.

Metspalu M, Romero IG, Yunusbayev B, et al (2011) Shared and unique components of human population structure and genome-wide signals of positive selection in South Asia. Am J Hum Genet 89:731–44.

Sudmant PH, Rausch T, Gardner EJ, et al (2015) An integrated map of structural variation in 2,504 human genomes. Nature 526:75–81.

Yunusbayev B, Metspalu M, Järve M, et al (2012) The Caucasus as an asymmetric semipermeable barrier to ancient human migrations. Mol Biol Evol 29:359–65.

Table S4. Putative origin for the uniparental lineages found in the 1KGP South Asian populations.

	Source	Haplogroups
**mtDNA**	Africa	L2, L3
	East Asia	A4, D4, F1, G3, N21
	South Asia (pre-LGM)	M, M18, M18'38, M2, M3, M30, M33, M34, M35, M36, M37, M38, M39, M39'70, M4, M40, M41, M42, M43, M44, M45, M4'67, M49, M5, M50, M52, M53, M6, M64, M65, M66, N5, R, R30, R31, R5, R6, R7, R8, U2
	West Eurasia	H, H13, H2, H29, H6, H7, HV, HV12, HV13, HV14, HV2, I1, J1, K1, K2, N1, R0, R2, R2'JT, T1, T2, U1, U5, U7, U9, W1, W4, W6, X
	**Source**	**Haplogroups**
**Y-chromosome**	East Asia	C(xC5), O, N
	South Asia (pre-LGM)	C5, H, K2a1*
	West Eurasia	G, J, L1, N, Q(xQ-L53), R1, R2
